# Vision protection therapy for prevention of neovascular age-related macular degeneration

**DOI:** 10.1038/s41598-023-43605-w

**Published:** 2023-10-04

**Authors:** Jeffrey K. Luttrull, Gerry Gray, Sathy V. Bhavan

**Affiliations:** 1 Ventura County Retina Vitreous Medical Group, Ventura, CA USA; 2 Regulatory Pathways, Inc, Laguna Beach, CA USA; 3 Ventura County Retina Vitreous Medical Group, 3160 Telegraph Rd, Suite 230, Ventura, CA 93003 USA

**Keywords:** Biophysics, Neuroscience, Diseases, Health care, Medical research, Risk factors, Optics and photonics

## Abstract

To access the effect of vision protection therapy on neovascular conversion in age-related macular degeneration (AMD). Patient unidentified data aggregated by Vestrum Health, LLC (VH) from over 320 US retina specialists was analyzed to compare the conversion rate from dry to neovascular (wet) AMD in a practice employing VPT (VPT group) compared to those employing standard care alone (SCA group) between January 2017 through July 2023. 500,00 eyes were filtered then matched for neovascular conversion risk factors by propensity scoring and compared in a 10/1 ratio of 7370 SCA and 737 VPT treated eyes. SCA eyes had significantly fewer clinical encounters and shorter follow up than the VPT group. Despite this, the risk of neovascular conversion by PS was significantly lower in the VPT group compared to SCA (HR 5.73, p < 0.0001). Analysis matching the encounter frequency of both groups as a post-randomization variable produced a similar HR (HR 5.98, p < 0.0001). Because 9% of eyes in the VPT group were not treated with VPT due to bilateral early (low-risk) AMD, analysis comparing the SCA group to VPT-treated eyes was done that also showed significantly lower conversion rates in the VPT-treated eyes, with or without encounter frequency matching (HR 5.84, 5.65, p < 0.0001). Visual acuity was consistently better in VPT eyes compared to SCA eyes throughout the study time window. The advantage of VPT over SCA increased with increased SCA encounter frequency and higher conversion risk factors, including age and ICD10 coded dry AMD severity. Neovascular (wet) AMD is the main cause of irreversible visual loss worldwide. Consistent with two prior studies, the current study finds Vision Protection Therapy markedly more effective at both recognizing and preventing neovascular AMD than the current standard of care, benefiting the highest risk dry AMD eyes the most.

## Introduction

Panmacular low-intensity/high-density subthreshold diode micropulse laser (SDM™) epitomizes the twin principles of modern retinal laser therapy^1–4^. First, selective thermal photostimulation of the retinal pigment epithelium (RPE) using laser parameters identical (“fixed”) in every eye that are reliably sublethal to the retina to preclude adverse treatment effects, while still exceeding the activation threshold for RPE heat-shock proteins (HSP) to cause a hormetic “reset” effect at the cellular level by upregulating of the endoplasmic reticulum unfolded protein response. Second, this reset normalization of function of RPE cells directly exposed to laser irradiation is then amplified to maximize the clinical effects of treatment by confluent/contiguous treatment of all retina between the major vascular arcades (“panmacular” treatment) to recruit *en masse* all dysfunctional retina directly exposed to treatment^1–4^. Panmacular SDM is thus a standardized and uniform treatment using the same laser settings, treatment area, treatment density and number of laser spot applications in every eye without adjustment for individual variations such as cataract, retina thickness, or RPE pigment density^1–4^. “Vision protection therapy™” describes SDM performed on a regular basis to maintain the treatment effects over time^5–9^.

The chronic progressive retinopathies (CPRs) represent a wide range of disparate conditions which, on the surface, seem to share little in common. Examples include retinitis pigmentosa, AMD, and diabetic retinopathy. However, all share the commonality of being neurodegenerations. In the CPRs, SDM has been found to improve retinal and visual function by all measures in dry AMD, open angle glaucoma and inherited retinal degenerations; reverse progression of diabetic retinopathy; effectively treat diabetic macular edema and central serous chorioretinopathy; reverse tolerance to anti-VEGF medications in wet AMD; and slow progression of age-related geographic atrophy^1–23^. SDM is anti-inflammatory, therapeutically immunomodulating, neuroprotective, neuroenhancing, and may be neuroregenerative in in vitro, in vivo and in human clinical and proteomic studies^5–23^. By improving, and then maintaining improved retinal function over time, VPT is intended to slow disease progression and reduce the risks of visual loss in the CPRs. This includes the risk of neovascular conversion in AMD^8,9,15,23^.

In 2018, a large retrospective cohort study of “all-comers” reported that SDM in a program of VPT reduced the expected incidence of neovascular conversion in a high-risk population with dry AMD by 95–98% per year^8^. In a second study, real-world data (RWD) was used to assess the efficacy of VPT using propensity scoring (PS) to match eyes receiving SCA to those receiving VPT as well. Starting with a pool of over 400,000 eyes with dry AMD in the 4.75-year study window, VPT significantly reduced neovascular conversion compared to SCA (hazard ratio of 13.04)^9^.

A limitation of the prior propensity scored RWD study was that the study time frame required inclusion of International Classification of Diseases version 9 data (ICD9) stratifying AMD only by “dry” and “wet”, precluding identification and matching of dry AMD subtypes. The current study addresses this limitation by including only eyes categorized with ICD10 codes after January 2017, which stratify dry AMD into risk and severity categories of “early”, “intermediate”, and “advanced with extrafoveal geographic atrophy” and “advanced with subfoveal geographic atrophy”.

## Methods

### Data source

As described in a prior study, Vestrum Health, LLC (Naperville, Ill) (VH) is database that aggregates unidentified patient data from the electronic medical records systems (EMR) of over 320 geographically diverse retinal subspecialty practices across the United States^9^. All study data for both comparison groups was obtained from the VH database. Data acquisition, filtering, application of study inclusions and exclusions, and propensity scoring was performed by VH. As this study is limited to analysis of patient unidentified electronic data provided by VH and did not involve human experimentation, patient informed consent was not required or obtained, and the study was exempted from investigational review board review (Western Institutional Review Board). The study adheres to the Health Insurance Portability and Accountability Act and Helsinki Declaration for Medical Research. All data generated or analyzed during this study are included in this published article and its Supplementary Information files^9^.

### Study groups

This study compares two cohorts of eyes with dry AMD active in the VH database between January 2017 through July 2023: one, a vitreoretinal group practice employing VPT in addition to standard care (recommendation of antioxidant vitamins, blood pressure control, smoking avoidance and healthy diet and lifestyle habits) in routine management of dry AMD, and the control group consisting of the rest of the VH vitreoretinal practices that did not employ VPT for dry AMD, but instead relied on standard care alone (SCA)^24^. The study groups are thus identified as VPT vs. SC alone (SCA).

### Study endpoints

The primary endpoint of the current study was to compare the rate of conversion from dry to wet AMD between the VPT and SCA cohorts. Neovascular conversion required 2-factor identification in the VH database including both a change in ICD10 diagnostic coding from dry to wet AMD (H35.30); and initiation of anti-vascular endothelial growth factor (VEGF) therapy. The date of conversion was defined as the earliest of the two events.

Visual acuity (VA) was a secondary endpoint. VA was scored by VH using an approximation of ETDRS measurement, according to the method of Gregori et al., by converting Snellen VA of 20/20, to 85 letters; 20/40 to 70 letters; 20/80 to 55 letters; 20/160 to 40 letters, etc.^25^.

### Inclusion and exclusion criteria

Inclusion criteria were an age of 50 years or more, and ICD10 coding for the diagnosis of dry AMD. Exclusion criteria for all subjects included prior or current intra-vitreal injections of steroids or VEGF inhibitors for any indication; neovascular AMD in the fellow eye; diabetes mellitus or retinal vascular occlusion as potential confounding causes of the need for intravitreal injections; prior conventional macular photocoagulation or other macular scarring; or concurrent diagnoses of ocular histoplasmosis, high/degenerative myopia, idiopathic macular telangiectasis or central serous chorioretinopathy as potential confounding causes of macular neovascularization. The influence of race on neovascular conversion risk is reflected in the presence and severity of dry AMD, accounted for in this study with ICD10 AMD severity subgroups.

### Propensity scoring

After application of inclusion and exclusion filters, eyes from the VPT and SCA cohorts were matched using propensity score methods based on patient characteristics^26–30^. Propensity scoring is the most rigorous method for statistically analyzing large existing populations or databases because all study variables are defined prior to randomization and matching, minimizing the potential for various biases. Propensity scores provide samples that are, in aggregate, balanced on all covariates included in the model. In a randomized clinical trial (RCT), presenting patients matching the study inclusion criterion are randomly distributed between study groups. Whereas clinical trials match and randomize subjects prospectively, propensity scoring randomly matches subjects retrospectively from an existing population^26–30^. Major neovascular conversion risk factors including age, AREDS vitamin use, diagnosis of systemic hypertension, and smoking were included in the propensity score modeling as well as dry AMD severity levels indicated by ICD10 coding for early, intermediate, or advanced dry AMD with and without subfoveal involvement^31,31^. Using the resulting propensity scores, all VPT cohort eyes were nearest-neighbor matched with all SCA cohort eyes in a 1/10 ratio (VPT/SCA)^26–30^. For the SCA group, the date of study entry was the date of first diagnosis of dry AMD, and for the VPT group, the date of initial treatment. The R “Matchit” package was used to carry out the propensity score matching. After propensity score matching, subjects were divided into 5 strata based on propensity scores, and standard stratified analyses were applied. Propensity score stratification is analogous to a meta-analysis of a set of quasi-RCTs within each quintile stratum^26,29^. Treatment assignment by the propensity score stratification process can be thought of as a random assignment conditional on observed covariates, such that the potential outcomes are independent of the treatment status^28^. Use of 5 strata can remove approximately 90% of any bias that remains following propensity scoring. Additional strata do not significantly improve performance^28^. Confidence intervals were calculated using bootstrap methods to account for inter-eye correlation (Supplemental Data).

Review of the post PS cohort demographics revealed a significant difference in the frequency of patient encounters between the groups, unmatched by PS as not a risk factor for conversion. In addition, because eyes with bilateral dry AMD did not generally receive VPT, not all eyes in the VPT cohort were treated with VPT. To gain insight as to how these factors might influence the results additional analyses were performed with encounter frequency and VPT treatment as post-randomization variables. There were therefore four separate data sets analyzed, with and without non-SDM-treated patients in the VPT cohort and with and without encounter matching.

### VPT treatment

VPT was offered to all patients with intermediate or advanced dry AMD in at least one eye in the VPT treatment group. VPT consisted of regular periodic panmacular SDM treatment performed every 3–4 months in a maintenance program intended to maximize treatment benefits over time. The treatment technique and parameters follow the guidelines for treatment recommended by the International Retinal Laser Society and are discussed in detail elsewhere^2,15^.

The following SDM treatment protocol was employed throughout the current study: Neither topical anesthesia nor pupillary dilation are employed. The macula is visualized with a 90 diopter non-contact lens at the slit lamp using only the aiming beam for illumination. With the aiming beam, the optic nerve is identified to orient the surgeon to the panmacular region. Once oriented, confluent laser spots are applied throughout the retina encompassed by the major vascular arcades including the fovea (“panmacular SDM”). The SDM laser parameters are wavelength 810nm, aerial spot size 300um, power 1.7 watts, spot duration 0.30 s, duty cycle 5%, number of spot applications per panmacular treatment session 400–450. These same laser parameters, treatment area, and number of spot applications are used in all eyes without adjustment for patient-specific factors such as fundus pigmentation, macular findings, or cataract. SDM is thus a uniform and standardized approach to microsecond pulsed laser therapy that is identical in all eyes of all patients without individual variation^1–9,15^.

## Results

### Demographics

The Vestrum database of approximately 500,000 eyes with visits between 1/3/2017 and 7/31/2023 were initially filtered using study inclusion and exclusion criteria to obtain a candidate set of approx. 200,000 eyes, including 737 VPT treated eyes. After 10:1 PS matching, the analysis data sets comprise 737 VPT eyes (from 406 subjects) from the VPT group, matched with two distinct sets of subjects from the remaining VH practices: 7370 SCA eyes from 4661 subjects without encounter matching and 7370 SCA eyes from 4652 subjects with encounter matching. Demographic characteristics were well matched (Tables 1, 2).

**Table 1:** Demographics by study group, after propensity score matching.

Factor level	VPT	SCA
N (study eyes)	737	7370
N (subjects)	406	4652
Gender		
Female	249/406 (61.3%)	2738/4652 (58.9%)
Male	157/406 (38.7%)	1858/4652 (39.9%)
Other	0/406 (0.0%)	56/4652 (1.2%)
Age (years)		
Mean(SD)	77.7 (9.1)	77.8 (9.2)
Median	78.0	78.0
Min, Max	[54.0, 93.0]	[50.0, 93.0]
Age (category)		
Age: [50,65]	32/406 (7.9%)	442/4652 (9.5%)
Age: (65,70]	56/406 (13.8%)	599/4652 (12.9%)
Age: (70,75]	77/406 (19.0%)	784/4652 (16.9%)
Age: (75,80]	79/406 (19.5%)	971/4652 (20.9%)
Age: (80,85]	72/406 (17.7%)	820/4652 (17.6%)
Age: (85,90]	53/406 (13.1%)	552/4652 (11.9%)
Age: (90,110]	37/406 (9.1%)	484/4652 (10.4%)
Hypertension		
No	185/406 (45.6%)	2139/4652 (46.0%)
Yes	221/406 (54.4%)	2513/4652 (54.0%)
AREDS use		
No	206/406 (50.7%)	2463/4652 (52.9%)
Yes	200/406 (49.3%)	2189/4652 (47.1%)
Smoking		
No	393/406 (96.8%)	4512/4652 (97.0%)
Yes	13/406 (3.2%)	140/4652 (3.0%)
AMD severity		
Early	91/406 (22.4%)	988/4652 (21.2%)
Unspecied	23/406 (5.7%)	273/4652 (5.9%)
Intermedte	235/406 (57.9%)	2621/4652 (56.3%)
NonCentralGA	41/406 (10.1%)	368/4652(7.9%)
CentralGA	39/406 (9.6%)	454/4652 (9.8%)

*AMD* age-related macular degeneration, *VPT* vision protection therapy, *SCA* standard care alone, *SDM* low-intensity/high-density subthreshold diode microsecond pulsed laser, *DME* diabetic macular edema, *DR* diabetic retinopathy, *RVO* retinal vein occlusion, *PS* propensity scoring, *N* number, *SD* standard deviation, *AREDS* age related eye disease study antioxidant vitamins, *Mini* minimum, *Max* maximum.

**Table 2:** Follow-up and treatment summary by study group, after propensity score matching.

Factor level	VPT	SCA
N (study eyes)	737	7370
Total follow-up days		
Mean (SD)	677.0 (581.8)	542.6 (633.2)
Median	497.0	232.5
Min, max	[4.0, 2248.0]	[0.0, 2392.0]
Follow up years (categories)		
0 ≤ follow up years ≤ 1	295/737 (40.0%)	4834/7370 (65.6%)
1 < follow up years ≤ 2	175/737 (23.7%)	1054/7370 (14.3%)
Follow up years > 2	252/737 (34.2%)	1265/7370 (17.2%)
Number of encounters		
Mean (SD)	13.2 (11.0)	11.4 (11.9)
Median	10.0	6.0
Min, max	[2.0, 53.0]	[2.0, 84.0]
Number of anti-VEGF injections per eye		
Mean (SD)	0.138 (0.834)	2.449 (6.976)
Median	0.000	0.000
Min, max	[0.000, 11.000]	[0.000, 55.000]
Treated with SDM laser		
No	0/737 (0.0%)	N/A
Yes	737/737 (100.0%)	N/A
Number of laser treatments		
Mean (SD)	6.3 (5.0)	N/A
n	737	N/A
Min, median, max	1, 5.0, 38	N/A
Converted to wAMD		N/A
Yes	32/737 (4.3%)	1202/7370 (16.3%)
No	705/737 (95.7%)	6168/7370 (83.7%)

*VPT* vision protection therapy, *SCA* standard care alone, *SD* standard deviation, *Min* minimum, *Max* maximum, *N* number, *wAMD* wet or neovascular age-related macular degeneration.

### Outcomes

**Figure 1:**
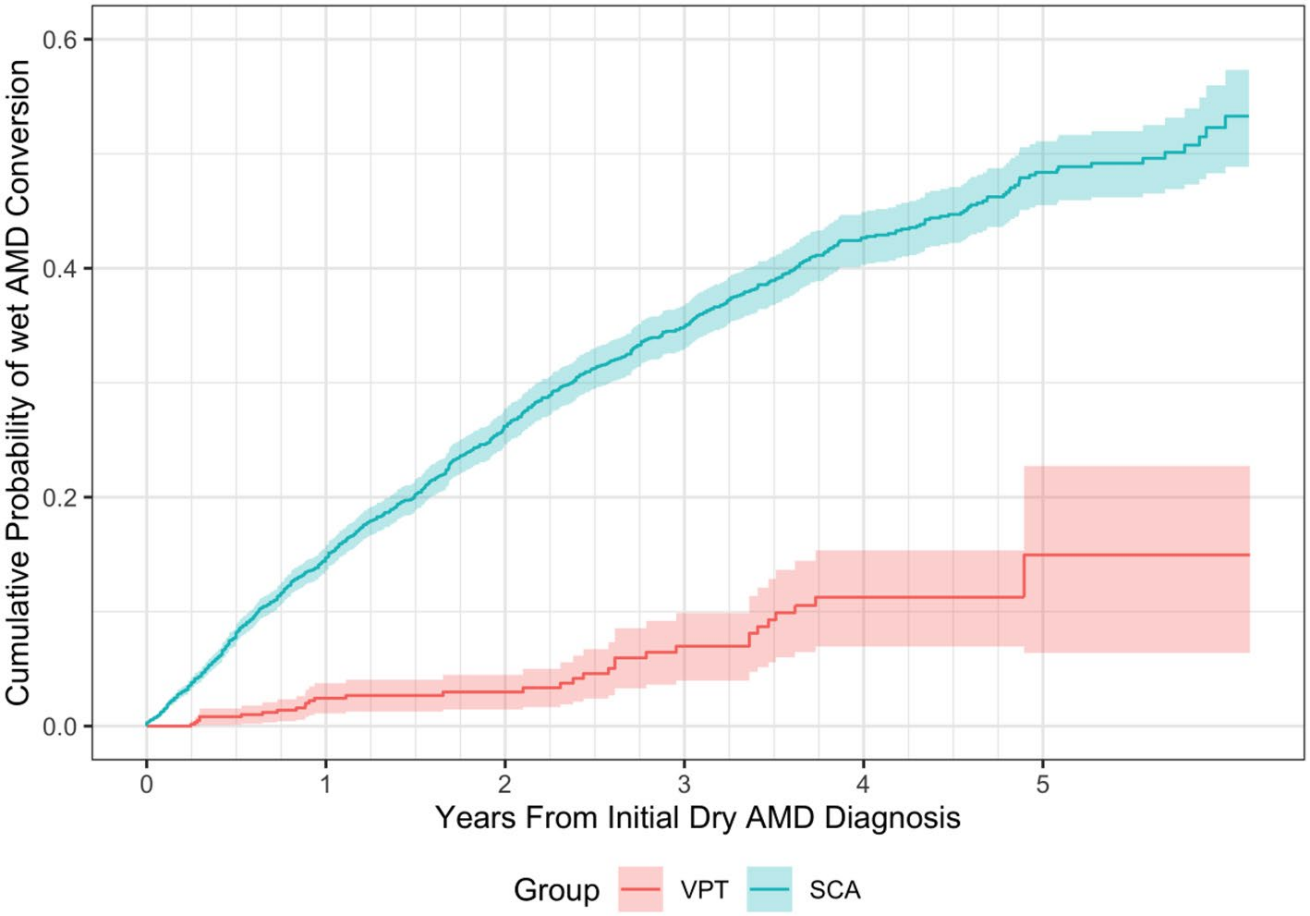
Overall Kaplan–Meier cumulative wet AMD conversion probability by group, ignoring covariates, with encounter matching. Shaded areas indicate 95% confidence intervals. *AMD* age-related macular degeneration, *SCA* standard care alone, *VPT* vision protection therapy.

Matching on the clinical encounter frequency, the rate of neovascular conversion was found to be 4.3% for the VPT-treated eyes compared (avg 13.2 encounters) to 16.3% for the SCA group (avg 11.4 encounters) (hazard ratio 5.98) (Fig. 1). Absent encounter matching the advantage to the VPT-treated eyes remained highly significant compared to the SCA group (avg 8.8 encounters, 12.5% conversion rate, HR 5.73) (Supplemental Data).

**Figure 2:**
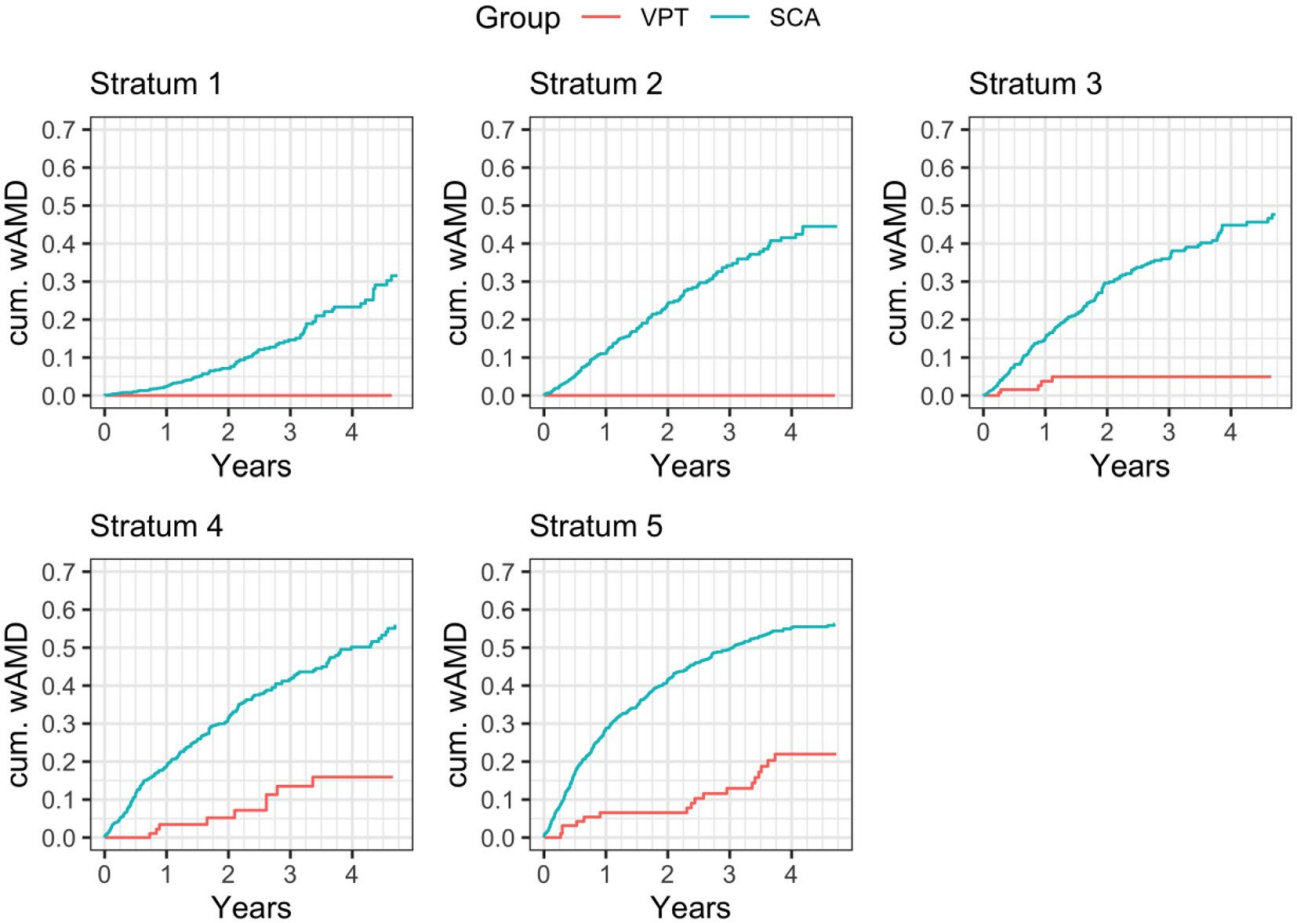
Cumulative probability of neovascular AMD conversion by propensity score strata 1–5. The survival analysis was stratified by propensity score quintiles. That is, eyes were divided into five (nearly equal size) groups using the quintiles of the propensity scores, from lowest (1st quintile) to highest (5th quintile) risk. The plots show the survival curves by propensity score stratum. Note that for every risk factor matched quintile, VPT appears to significantly reduce the rate of neovascular conversion compared to standard care alone. *VPT* vision protection therapy, *SCA* standard care alone, *AMD* age-related macular degeneration, *Cum* cumulative.

A survival analysis was stratified by propensity score quintiles. That is, eyes were divided into five (nearly equal size) groups using the quintiles of the propensity scores. For every quintile, the risk of conversion was significantly lower in the VPT treated eyes compared to SCA (Fig. 2). A test for equality of the Kaplan–Meier survival curves (a stratified log-rank test) shows a very significant difference in survival between the VPT-treated and SCA groups (Table 3).

**Table 3:** A test for equality of the Kaplan-Meier survival curves (a stratified log-rank test) shows a very significant difference in survival between the VPT and SCA groups.

	N	Observed	Expected	(O-E)^2^/E	(O-E)^2^/V
Analysis.Group = VPT	737	32	170.1	112.1	131.4
Analysis.Group = SCA	7370	1202	1064	17.93	131.4

Kaplan–Meier test between Groups, stratified by propensity score quintiles. Chisq = 107.736351 on 1 degrees of freedom, p = 0.000000.

*VPT* vision protection therapy, *SCA* standard care alone, *O* observed, *E* expected, *N* number, *V* variance.

For severity, the “early” group (used as the reference group in the Cox Proportional Hazards (CPH) model) had the lowest hazard, with all the other groups producing a hazard ratio > 1. The “Intermediate”, “Non-Central GA”, and “Central GA” groups all showed significant increase in hazard over the baseline (with HRs ranging from 1.66 to 1.99). The “Unspecified” group showed a non-significant increase in hazard (HR 1.22).

**Figure 3:**
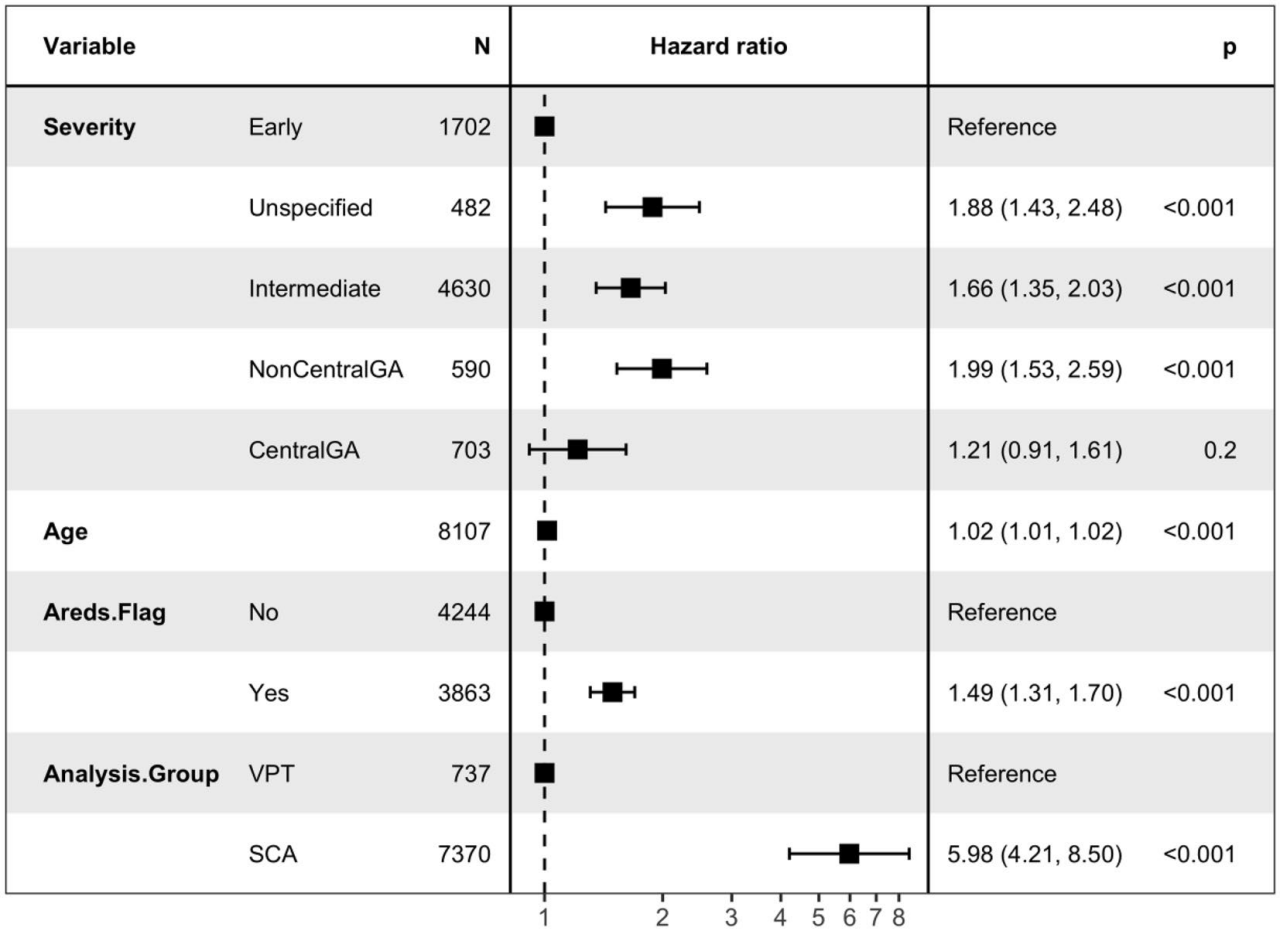
Forest plots of Cox PH results. *PH* proportional hazards, *N* number, *SCA* standard care alone, *VPT* vision protection therapy, *AREDS* age-related eye disease vitamin use, *P* probability, *GA* geographic atrophy.

There was a significant 2% increase in hazard for each increased year of age (HR 1.02, p ≤ 1e−04). Subjects with AREDS use showed an approximate 50% increase in hazard (HR 1.49, p ≤ 1e−04). There was a significant increase in hazard for the SCA group, after adjusting for severity, age, and AREDS use (HR 5.98, p ≤ 1e−04) (Table 4) (Fig. 3). The overall test, and tests for all included factors, showed high levels of significance (Table 5). A test for the proportional hazards assumption shows strong evidence of non-proportionality (cox.zph), p < 0.001). However various diagnostic plots do not indicate strong non-proportionality in the Cox PH model Supplemental Data). Small p-values could be due to the large sample size and not a meaningful departure from proportional hazards (Table 6). Table 7 shows the difference in conversion rates over time.

**Table 4:** Cox PH summary of survival difference.

	Coef	Exp(coef)	Se(coef)	z	p
SeverityUnspecified	0.6335	1.884	0.1404	4.513	6.405e−06
SeverityIntermediate	0.5057	1.658	0.1036	4.881	1.057e−06
SeverityNonCentralGA	0.6886	1.991	0.1348	5.109	3.23e−07
SeverityCentralGA	0.1946	1.215	0.1448	1.343	0.1791
Age	0.01495	1.015	0.003706	4.035	5.457e−05
Areds.FlagYes	0.3989	1.49	0.06654	5.994	2.045e−09
Analysis.GroupSCA	1.788	5.978	0.1794	9.968	0

Estimated hazard ratios are in the column labeled exp(coef). Likelihood ratio test = 304.64 on 7 df, p = 0, n = 7425, number of events = 1102. Likelihood ratio test = 304.64 on 7 df, p = 0, n = 7425, number of events = 1102.

*GA* geographic atrophy, *SCA* standard care alone, *Coef* coefficient, *P* probability value, *Z* z score, *SE* standard error of the mean, *PH* proportional-hazards regression model.

**Table 5:** ANOVA for Cox PH model, VPT non-laser excluded, with encounter matching.

	loglik	Chisq	df	Pr( >|Chi|)
NULL	− 7745	NA	NA	NA
Severity	− 7717	57.11	4	1.174e−11
Age	− 7709	15.14	1	9.958e−05
Areds.Flag	− 7689	39.87	1	2.714e−10
Analysis.Group	− 7598	183.5	1	8.524e−42

*ANOVA* one-way analysis of variance, *PH* proportional-hazards regression model, *AREDS* age related eye disease vitamin use, *Loglik* logical likelihood, *Chisq* Chi-square, *Df* degrees of freedom, *PR* probability.

**Table 6:** Tests for proportional hazards violations, VPT non-laser excluded, with encounter matching.

	Chisq	df	p
Severity	21.91	4	0.0002086
Age	0.1243	1	0.7244
Areds.Flag	3.604	1	0.05766
Analysis.Group	7.135	1	0.007559
GLOBAL	33.4	7	2.232e-05

*Chisq* Chi-square, *Df* degrees of freedom, *P* probability.

**Table 7:** Summary of overall survival by group (unstratified Kaplan–Meier estimates), VPT non-laser excluded, with encounter matching.

Analysis group	Years from DAMD diagnosis	N at risk	N events	Cumulative probability of wet AMD (%)	95% CI
VPT	1	442	13	2.4	[1.1%, 3.7%]
	2	267	2	3.0	[1.5%, 4.5%]
	3	175	9	7.0	[4.0%, 9.9%]
	4	105	7	11.3	[7.0%, 15.4%]
SCA	1	2536	641	14.7	[13.6%, 15.8%]
	2	1482	283	26.2	[24.6%, 27.8%]
	3	884	148	34.9	[32.9%, 36.8%]
	4	483	87	42.7	[40.3%, 44.9%]

*DAMD* dry age-related macular degeneration, *VPT* vision protection therapy, *SCA* standard care alone, *N* number, *CI* confidence interval.

The hazard ratio between the VPT-treated eyes and SCA groups is summarized in Table 8. To check for inter-eye correlation because there are multiple eyes per person, a clustered bootstrap (clustered by subject) was used to provide a robust check on the confidence interval. The lower bound on the 95% confidence interval for the hazard ratio is above 5 using either method, again providing strong evidence for a hazard ratio greater than 1.

**Table 8:** Cox proportional hazards estimated hazard ratio and associated confidence intervals, VPT non-laser excluded, with encounter matching.

Estimated hazard ratio	95% CI (asymptotic)	95% CI (bootstrap^1^)
6.0	[4.2, 8.5]	[4.3, 9.3]

Cox PH model is stratified by propensity score quartiles. 1-Bootstrap confidence interval is based on 10,000 cluster (subject level) bootstrap samples.

*CI* confidence interval.

In all analyses, VA trended better in the VPT group compared to the SCA group throughout the 6.5 year study window (Table 9) (Supplemental Data).

**Table 9:** Mean visual acuity (ETDRS letters or equivalent) by group and year, VPT non-laser excluded, with encounter matching.

Analysis group	Mean VA 2017	Mean VA 2018	Mean VA 2019	Mean VA 2020	Mean VA 2021	Mean VA 2022
VPT	71.2	67.0	68.1	70.4	67.9	66.9
SCA	66.7	66.9	65.5	65.1	63.9	63.5

Cox PH model is stratified by propensity score quartiles.

*VA* visual acuity, *ETDRS* early treatment of diabetic retinopathy study.

Additional analyses were performed to test the effect of retention of some SDM-untreated eyes in the VPT comparison group in the primary analysis prior to propensity score matching, thus avoiding any bias due to “post-randomization exclusion” of VPT subjects. This increased the number of eyes in the VPT group to 814 eyes in 441 patients and the SCA group to 8140 eyes of 5115 patients (without encounter matching) and 8140 eyes in 5114 patients (with encounter matching) in the SCA groups. These analyses produced hazard ratios of 5.65 and 5.84, both highly significant (p < 0.0001). Thus, in both the most statistically robust PC analysis that considered all eyes coded for dry AMD in both groups, and in the post hoc analyses that excluded VPT-untreated eyes from the VPT group, the risk of neovascular conversion was similarly and markedly reduced in the VPT group by every comparison to the SCA group (HR 5.65–5.98; all p < 0.0001) (Supplemental Data).

Although not a study endpoint, the number of anti-VEGF injections required in eyes after conversion within the study window was lower in the VPT group compared to the SCA group, with SCA eyes receiving between 3.9× (non-encounter matched) and 4.5× (encounter matched) more injections than VPT group or VPT-treated eyes after neovascular conversion (Supplemental Data).

## Discussion

Standard care for dry AMD currently consists of antioxidant nutritional supplements including AREDS (age-related eye disease study) vitamins; a diet rich in green, leafy vegetables; lifestyle measures such as smoking cessation and exercise; and healthcare measures such as treatment of systemic hypertension^24,31^. Implementation of such measures has been shown to reduce the risk of developing wet, neovascular AMD by about 4% per year^31^. As the main cause of irreversible visual loss and visual disability worldwide, more effective treatment to prevent wet AMD is a major public health care priority^32,33^.

This is the third in a series of studies examining the effect of VPT on neovascular conversion in AMD^8,9^. While there is some overlap at the margins, each used different methods, different inclusion and exclusion criteria, different patients, and different time frames for study. Only the treatment and study endpoint were the same. Despite these differences, all three studies find that VPT markedly reduces the risk of neovascular conversion in dry AMD compared to SCA.

SDM, serialized in VPT, epitomizes the concepts of modern retinal laser therapy, defined by treatment that is sublethal to the retinal pigment epithelium (RPE) affording reliable clinical safety; then optimized by maximization of the treatment effect at the cellular level by *en masse* functional transformation via confluent treatment and recruitment of large areas of dysfunctional retina (such as “panmacular” treatment)^1–16^. That SDM is without known adverse treatment effects is attributable to the very wide therapeutic range achievable using specific laser parameters^1–16^. These parameters have been developed with the aid of mathematical modeling informed by over 20 years of clinical data (Chang DB, Scaling Law Analysis, proprietary data Ojai Retinal Technologies, LLC, Los Angeles, California). Because reliable treatment safety is paramount, and recognizing that panmacular SDM by definition includes treatment of the fovea, any deviation from published SDM parameters known to be safe and effective in all eyes in extensive long-term clinical use, including the laser parameters used in the current study, is strongly discouraged^2,14,15^.

Morphologic analysis was not part of the current study. Gradual resolution has been noted in the course of VPT for dry AMD^15^. However, it is important to note that, because SDM is sublethal to the RPE, SDM does not affect or reduce drusen in the short-term, nor is it the intent of treatment, as acute drusen reduction results from debridement precipitated by laser-induced retinal damage (LIRD). Such LIRD is associated with disease acceleration and an increased, rather than decreased, risk of choroidal neovascularization^5,6,15,22,34–36^. Instead, the benefits of SDM arise entirely from normalization of retinal function by laser thermohormesis sublethal to the RPE. VPT then maintains these improvements over time to slow disease progression and reduce the risks of visual loss, such the reduction in neovascular conversion in AMD reported in the current study^2–11,14,15,22,23^.

The current study was limited to subjects for whom ICD-10 coding was used to allow matching of dry AMD severity (early, intermediate, or advanced with either subfoveal or extrafoveal geographic atrophy). Site-level assessment of AMD severity assumes a reasonably consistent diagnostic coding judgement across all VH sites. As a RWD study, there was no reading center to verify coding accuracy as would be implemented in a clinical trial. However, while some variation is likely, such variations would be expected to be offset by the large sample size and PS matching of all conversion risk factors^25–30^.

Propensity scoring is the gold-standard of statistical analysis for large pre-existing populations, typically the subject of real-world data studies such as the current study^26–30^. Potential biases are minimized by identifying all key study variables prior to randomization and matching. Quintile analysis can then be performed to identify unrecognized biases. In the current study, there is no evidence by quintile analysis of unrecognized bias (Fig. 2) (Supplemental Data).

Propensity score methods can balance the two groups with respect to the variables used in the propensity score model^28–30^. However, they do not balance for unmeasured covariates. Thus, if there are other important predictors of group membership or outcome that are missing the results could be misleading. The only major risk factors for neovascular conversion not specifically matched in the current study are the presence or absence of reticular pseudodrusen (RPD), and genetic predisposition, information about which is unavailable in the VH database^8,37,38^. However, as both are associated with patient age and AMD severity, PS matching of age and AMD severity by ICD10 mitigate the absence of genetic and RPD data.

Review of the PS analysis revealed that patients in the SCA group were seen significantly less often than the VPT group, and that not all (9%) patients in the VPT were VPT treated (Supplemental Data). Because a patient encounter is required for the diagnosis of conversion, the lower frequency of examinations might lead to fewer identified neovascular conversions. Patients with bilateral early AMD were generally not treated with VPT unless an increased risk was indicated, usually by abnormal dark adaptometry testing^15^. Thus, not all eyes in the VPT group were VPT-treated^15^. Therefore, post hoc analyses were performed matching the encounter frequencies between groups, and eliminating VPT untreated eyes from the VPT group, to gain insight into how these asymmetries might have influenced the PS results, for a total of 4 separate analyses. Thus, in addition to the wholly randomized and matched PS comparison of the VPT and SCA groups, the post hoc analyses used encounter frequency and VPT treatment as post randomization variables^26–30^. While use of post randomization variables weakens the analysis by increasing the potential for biases, these post hoc analyses preserved PS randomization and matching of the other pre-randomization variables to facilitate group comparisons.

A patient encounter, typically including a clinical examination and macular imaging, is usually required to identify neovascular conversion in AMD. Thus, the more often a patient is encountered, the more likely (earlier) a conversion event will be identified if one has occurred in the interim between encounters. Thus, while a patient encounter is not a risk factor for wet AMD, it is for identification of conversion. VPT is a program of regular periodic SDM treatment designed to maintain the treatment benefits over time in chronic progressive retinopathies to slow progression and reduce the associated risks of visual loss^4–9^. Thus, in the current study, VPT patients were examined with optical coherence tomography much more frequently than SCA eyes (Table 1) (Supplemental Data). The mean number of encounters for the VPT group was 13.2 compared to 8.8 for the SCA group, a 50% difference, with conversion rates of 4.3% for the VPT group and 12.5% for the SCA group. PS matching could not equalize the exam frequencies between the groups due to the infrequency of encounters in the SCA group, but did increase the SCA exam frequency to a mean 11.4 visits, still 16% lower than the VPT group. However, it is notable that this 30% increase in exam frequency for the SCA group resulted in a 30% increase in diagnosed conversions from 12.5 to 16.3%, or a 1% increase in documented conversions in the SCA group for every 1% increase in encounters. This suggests that had the encounter frequency in the SCA group equaled the VPT group, an even higher conversion rate in the SCA group would have been observed. Because conversion risk factors remained matched by PS, the higher rate of conversions identified with increased encounters in the SCA group appears to be the result of the increased exam frequency alone. This suggests that the majority of neovascular conversions may not be identified by SCA, including in the current study, due to the infrequency of clinical examination. If so, the current study underestimates the benefit of VPT for preventing neovascular conversion in dry AMD.

In addition to differences in encounter frequency, the VPT group had longer follow-up compared to the SCA group, increasing the chances of neovascular conversion in the VPT group. (Tables 1, 2) Thus, both the asymmetries in the study populations, consisting of fewer encounters and shorter follow up in the SCA group, favor the SCA group by reducing the likelihood of both occurrence and identification of a conversion event compared to the VPT group (Tables 1, 2) (Supplemental Data).

Due to the greater data volatility in the smaller VPT group, VA data was not statistically analyzed. However, at every point in each analysis, VPT treated eyes demonstrated better VA than SCA eyes. This is consistent with the VA results of prior studies of SDM for AMD and other indications^1–23^.

Finally, although the post-conversion course was not an outcome measure of the current study, the number of anti-VEGF injections employed after neovascular conversion within the 6.5-year study window was recorded. It is interesting to note that VPT eyes required 3.9× to 4.5× fewer anti-VEGF injections than SCA eyes after neovascular conversion (Supplemental Data). This may be an artifact of the much smaller number of conversions in the VPT group. In a prior report, SDM was shown to reverse tolerance to anti-VEGF drugs in neovascular AMD^5^. In that study it was suggested that including SDM (as VPT) in the management of wet AMD might improve the performance of anti-VEGF medications administered over the long-term by helping to maintain drug sensitivity and avoid drug tolerance, and that this might manifest in the requirement for fewer injections^5^. A prior VH data analysis found that over a 5 years, period, inclusion VPT in wet AMD reduced anti-VEGF injections by an avg. 69% per eye without sacrificing VA compared to eyes managed with anti-VEGF injections alone^15^. Thus, in the current study VPT was continued after neovascular conversion (the primary study endpoint)^5,7^. The much lower frequency of post conversion anti-VEGF injections in the VPT group compared to the SCA group suggests this strategy may have been effective.

The current study employs RWD^26–30,39^. It is not an RCT. As a RWD study it is large, over a long time window (6.5 years), and compares eyes of patients across the United States cared for in a large proportion of US retinal practices matched for all available known neovascular conversion risk factors. Compared to RWD studies, RCTs are generally smaller, shorter, far more expensive, and more time consuming^39^. Because of the expense, few have the resources to perform RCTs. Thus, well over 95% of all RCTs in medicine, including ophthalmology, are funded by pharmaceutical companies who have a priori interests in their outcomes^39–41^. To address this issue and the concerns it raises, the Cochrane collaborative performed a meta-analysis comparing the results of RCTs and RWD studies^42^. The Cochrane study found no significant difference in the results or reliability between RCTs and RWD studies, provided the RWD studies were large and had robust results, like the current study^42^. We note that RWD studies are often used to confirm the results of RCTs, illustrated by the recent approvals of various COVID vaccines^43^. For the same reasons, RCTs are seldom used to verify RWD studies. In large part this is because RWD studies tend to represent a worst-case scenario for a given intervention. When there is variance between RCTs and RWD studies, the results of RWD tend to be worse, not better, than the relevant RCT. The post-FDA approval histories of recent drugs for AMD are illustrative in this regard^44–47^. This is because RCTs tend to be smaller, idealized, and performed under favorable and highly controlled conditions, unlike real-world clinical medicine. In the current study, key residual imbalances remaining after statistical matching between the study groups favor the SCA group. Finally, the results of the four distinct statistical analyses performed in the current study yielded virtually identical results. All of these considerations suggest that the results of the current study are reliable and likely underestimate the marked advantage of VPT for prevention of wet AMD, preventing and identifying neovascular conversion, and preventing visual loss, compared to the current standard of care for dry AMD^42–45^.

### Supplementary Information

Supplementary Information.

## Data Availability

All data generated or analyzed during this study are included in this published article and its Supplementary Information files.

## References

[1] Kozak I, Luttrull JK (2015). Modern retinal laser therapy. Saudi J. Ophthalmol..

[2] Keunen JEE, Battaglia-Parodi M, Vujosevic S, Luttrull JK (2020). International retinal laser society guidelines for subthreshold laser treatment. Transl. Vis. Sci. Technol..

[3] Chhablani J, Roh YJ, Jobling AI (2018). Restorative retinal laser therapy: Present state and future directions. Surv. Ophthalmol..

[4] Chang DB, Luttrull JK (2020). Comparison of subthreshold 577 nm and 810 nm micropulse laser effects on heat-shock protein activation kinetics: Implications for treatment efficacy and safety. Transl. Vis. Sci. Technol..

[5] Luttrull JK, Chang DB, Margolis BWL, Dorin G, Luttrull DK (2015). Laser re-sensitization of medically unresponsive neovascular age-related macular degeneration: Efficacy and implications. Retina.

[6] Luttrull JK, Margolis BWL (2016). Functionally guided retinal protective therapy as prophylaxis for age-related and inherited retinal degenerations. A pilot study. Investig. Ophthalmol. Vis. Sci..

[7] Luttrull JK, Sinclair SH, Elmann S, Chang DB, Kent D (2020). Slowed progression of age-related geographic atrophy following subthreshold laser. Clin. Ophthalmol..

[8] Luttrull JK, Sinclair SH, Elmann S, Glaser BM (2018). Low incidence of choroidal neovascularization following subthreshold diode micropulse laser (SDM) for high-risk AMD. PLoS ONE.

[9] Luttrull JK, Gray G (2022). Real world data comparison of standard care vs SDM laser vision protection therapy for prevention of neovascular AMD. Clin. Ophthalmol..

[10] Luttrull JK, Samples JR, Kent D, Lum BJ, Samples JR, Knepper PA (2018). Panmacular subthreshold diode micropulse laser (SDM) as neuroprotective therapy in primary open-angle glaucoma. Glaucoma Research 2018–2020.

[11] Luttrull JK (2018). Improved retinal and visual function following subthreshold diode micropulse laser (SDM) for retinitis pigmentosa. Eye (London)..

[12] Luttrull JK, Musch MC, Mainster MA (2005). Subthreshold diode micropulse photocoagulation for the treatment of clinically significant diabetic macular edema. Br. J. Ophthalmol..

[13] Luttrull JK, Spink CJ, Musch DA (2008). Subthreshold diode micropulse panretinal photocoagulation for proliferative diabetic retinopathy. Eye.

[14] Luttrull JK, Sramek C, Palanker D, Spink CJ, Musch DC (2012). Long-term safety, high-resolution imaging, and tissue temperature modeling of subvisible diode micropulse photocoagulation for retinovascular macular edema. Retina.

[15] Luttrull JK (2023). Modern Retinal Laser Therapy.

[16] Lavinsky D, Cardillo JA, Melo LA (2011). Randomized clinical trial evaluating mETDRS versus normal or high-density micropulse photocoagulation for diabetic macular edema. Investig. Ophthalmol. Vis. Sci..

[17] Chen G, Tzekov R, Li W (2016). Subthreshold micropulse diode laser versus conventional laser photocoagulation for diabetic macular edema. A meta-analysis of randomized controlled trials. Retina.

[18] Al-Barki A, Al-Hijji L, High R, Schatz P, Do D, Nguyen QD, Luttrull JK, Kozak I (2021). Comparison of short-pulse subthreshold (532 nm) and infrared micropulse (810 nm) macular laser for diabetic macular edema. Sci. Rep..

[19] Frizziero L, Calciati A, Torresin T, Midena G, Parrozzani R, Pilotto E, Midena E (2021). Diabetic macular edema treated with 577-nm subthreshold micropulse laser: A real-life, long-term study. J. Pers. Med..

[20] Jhingan M, Goud A, Peguda HK, Tyagi M, Luttrull JK, Chhablani J (2018). Subthreshold microsecond laser for proliferative diabetic retinopathy: A randomized pilot study. Clin. Ophthalmol..

[21] Luttrull JK (2016). Low-intensity/high-density subthreshold diode micropulse laser (SDM) for central serous chorioretinopathy. Retina.

[22] Luttrull JK, Kent D (2020). Laser Therapy to Prevent Choroidal Neovascularization.

[23] Luttrull JK, Kent D, Samples JR, Ahmed IIK (2019). Modern retinal laser for neuroprotection in open-angle glaucoma. New Concepts in Glaucoma Surgery.

[24] *American Academy of Ophthalmology Preferred Practice Pattern—Update 2015*. https://www.aao.org/preferred-practice-pattern/age-related-macular-degeneration-ppp-2015.

[25] Gregori NZ, Feuer W, Rosenfeld PJ (2010). Novel method for analyzing snellen visual acuity measurements. Retina.

[26] Kim SC, Schneeweiss S (2019). When randomized clinical trials and real-world evidence say the same: Tocilizumab and its cardiovascular safety. Arthritis Rheumatol..

[27] Jupiter DC (2017). Propensity score matching: Retrospective randomization?. J. Foot Ankle Surg..

[28] Rosenbaum PR, Rubin DB (1983). The central role of the propensity score in observational studies for causal effects. Biometrika.

[29] Austin PC (2011). An introduction to propensity score methods for reducing the effects of confounding in observational studies. Multivar. Behav. Res..

[30] Greenhouse JB (2009). Commentary: Cornfield, epidemiology and causality. Int. J. Epidemiol..

[31] Age-Related Eye Disease Study Research Group (2001). A randomized, placebo-controlled, clinical trial of high-dose supplementation with vitamins C and E, beta carotene, and zinc for age-related macular degeneration and vision loss: AREDS report number 8. Arch. Ophthalmol..

[32] Wan LW, Xinyi S, Xiang L (2014). Global prevalence of age-related macular degeneration and disease burden projection for 2020 and 2040: A systemic review and meta-analysis. Lancet Glob. Health.

[33] Ferris FL, Fine SL, Hyman L (1984). Age-related macular degeneration and blindness due to neovascular maculopathy. Arch. Ophthalmol..

[34] Virgili G, Michelessi M, Parodi MB, Bacherini D, Evans JR (2015). Laser treatment of drusen to prevent progression to advanced age-related macular degeneration. Cochrane Database Syst. Rev..

[35] Guymer RH, Wu Z, Hodgson LAB, Caruso E (2018). Laser intervention in early stages of age-related macular degeneration study Group. Subthreshold nanosecond laser intervention for age-related macular degeneration: The LEAD randomized controlled clinical trial. Ophthalmology..

[36] Roider J, Brinkmann R, Wirbelauer C, Laqua H, Birngruber R (2000). Subthreshold (retinal pigment epithelium) photocoagulation in macular diseases: A pilot study. Br. J. Ophthalmol..

[37] Armento A, Ueffing M, Clark SJ (2021). The complement system in age-related macular degeneration. Cell Mol. Life Sci..

[38] Mitchell P, Liew G, Gopinath B, Wong TY (2018). Age-related macular degeneration. Lancet.

[39] Schilsky RL (2017). Finding the evidence in real-world evidence: Moving from data to information to knowledge. JACS.

[40] Bhandari M, Busse JW, Jackowski D (2004). Association between industry funding and statistically significant pro-industry findings in medical and surgical randomized trials. CMAJ.

[41] Chalmers TC, Celano P, Sacks HS, Smith H (1983). Bias in treatment assignment in controlled clinical trials. Review. New Eng. J. Med..

[42] Anglemyer A, Horvath HT, Bero L (2014). Healthcare outcomes assessed with observational study designs compared with those assessed in randomized trials. Cochrane Database Syst. Rev..

[43] Dooling K, Gargano JW, Moulia D, Wallace M, Rosenblum HG, Blain AE, Hadler SC, Plumb ID, Moline H, Gerstein J, Collins JP, Godfrey M, Campos-Outcalt D, Morgan RL, Brooks O, Talbot HK, Lee GM, Daley MF, Oliver SE (2021). Use of pfizer-BioNTech COVID-19 vaccine in persons aged ≥16 years: Recommendations of the Advisory Committee on Immunization Practices—United States, September 2021. Morb. Mortal. Wkly. Rep..

[44] Ciulla TA, Huang F, Westby K (2018). Real-world outcomes of anti-vascular endothelial growth factor therapy in neovascular age-related macular degeneration in the United States. Ophthalmol. Retina.

[45] Ciulla TA, Hussain RM, Pollack JS, Williams DF (2019). Visual acuity outcomes and anti-vascular endothelial growth factor therapy intensity in neovascular age-related macular degeneration patients. A real-world analysis of 49,485 eyes. Ophthalmol. Retina.

[46] Witkin AJ, Hahn P, Murray TG (2020). Occlusive retinal vasculitis following intravitreal brolucizumab. J. Vitreoretin. Dis..

[47] *ASRS Research and Safety in Therapeutics (REST) Committee Update on Adverse Events Reports Associated with Syfovre*. https://www.asrs.org/clinical/clinical-updates/9327/ASRS-Research-and-Safety-in-Therapeutics-REST-Committee-Update-on-Adverse-Events.

