# Supplementary material for: Vision protection therapy for prevention of neovascular age-related macular degeneration
Source: Sci Rep. 2023 Oct 4;13:16710. doi: 10.1038/s41598-023-43605-w (PMC10550910; doi:10.1038/s41598-023-43605-w)
Supplement: Supplementary file 1 — Supplementary Information. [file 41598_2023_43605_MOESM1_ESM.pdf]

Retinal Protection Sciences

Comparison of SDM treatment to standard of care
Propensity matched Vestrum data,
with AMD severity coding
v6.0

[without non-laser-treated VPT subjects]
[with encounter matching]

Gerry Gray, Regulatory Pathways, Inc.

9/20/2023

Table of Contents

INTRODUCTION **2**
BASELINE TABULATIONS, VPT NON-LASER EXCLUDED, WITH ENCOUNTER MATCHING **4**
TREATMENT AND RAW OUTCOMES, VPT NON-LASER EXCLUDED, WITH ENCOUNTER MATCHING **5**
SURVIVAL ANALYSIS BY PS STRATUM, VPT NON-LASER EXCLUDED, WITH ENCOUNTER MATCHING **7**
SUMMARY OF SURVIVAL FITS, VPT NON-LASER EXCLUDED, WITH ENCOUNTER MATCHING **16**
VISUAL ACUITY, VPT NON-LASER EXCLUDED, WITH ENCOUNTER MATCHING **17**
CAVEATS FOR PROPENSITY SCORE ANALYSIS **21**
APPENDIX/SUPPLEMENTAL **22**
DISTRIBUTION OF FOLLOW-UP TIME 22
DIAGNOSTIC PLOTS FOR PROPENSITY SCORES 23
INCIDENCE RATES VIA POISSON REGRESSION 30
DIAGNOSTIC PLOTS FOR COX PH MODEL 31

Introduction

This report contains analyses of propensity score matched data from the Vestrum database.

This is the second propensity score analysis from this database. One of the major perceived shortcomings of the previous analysis was that AMD severity at diagnosis was not available in the then-current ICD-9 coding. This issue was addressed in the new ICD-10 codes, which indicate AMD severity at diagnosis. This new information on initial AMD severity was used in the revised propensity score matching.

This analysis includes severity of the initial AMD diagnosis, classified as "Early", "Unspecified", "Intermediate", "Non-Central GA" or "Central GA".

There are four separate versions of this report, created to satisfy a reviewer's request, to adhere to a principle of "no post-randomization exclusions" of subjects, and to evaluate the sensitivity of the analyses to differential follow-up intensity. The four versions are the combinations of the following:

SDM Laser Treatment

1. Including all subjects from the VPT group who met the initial filtering (I/E) criteria.
2. Including only VPT subjects who were treated with the SDM laser (a "post-randomization" event) in addition to meeting the I/E criteria.

Follow-up Intensity

1. Propensity score matching that includes follow-up intensity, using using the mean time between visits.
2. Propensity score matching that does not include follow-up intensity.

Throughout, we use the labels "VPT" to indicate Luttrull subjects, treated with standard of care and SDM laser as appropriate, and "SCA" to indicate the matched eyes from the Vestrum database.

The Vestrum database of ~500,00 eyes with visits between 1/2/2017 and 7/31/2023 were initially filtered using study inclusion/exclusion criteria to obtain a candidate set of ~200,000 eyes, including 814 VPT eyes (737 who were treated with SDM Laser). Although exclusion of VPT eyes based on treatment recieved during the study violates a statisical principle of "no post-randomization exclusions", at the insistence of reviewers we analyzed data both with and without VPT SDM laser treated eyes.

After completion of the initial filtering, and for both 1) all VPT eyes and 2) only SDM laser-treated VPT eyes, nearest-neighbor propensity score matching was used to obtain a matched set of control eyes from the SCA group. The R Matchit package was used for the matching (R version 4.0.2, Matchit version 3.0.2).

Propensity scores were based on the following covariates:

Table 1. Variables used to perform propensity score matching

Variable
Age
Smoking status
AREDS vitamin use status
Hypertension status
AMD Severity

To obtain similar follow-up intensity between the groups, a second propensity score matching used all of the above variables plus the mean time-between-visits. These analyses are labeled “with encounter matching” in the headings.

An earlier analysis of data from the Vestrum database was reported in REF. At the time of that analysis, the Vestrum system used the then-current ICD-9 codes for AMD.

With the transition to ICD-10, the codes for AMD now include separate categories for disease severity.

Table 2. ICD 10 AMD Severity codings.

AMD Severity	ICD Code	Description
Early	H35.31X1	early dry AMD—a combination of multiple small drusen (≤ 63 µm), few intermediate drusen (> 63 µm and ≤ 124 µm), or retinal pigment epithelium (RPE) abnormalities
Intermediate	H35.31X2	intermediate dry AMD—extensive intermediate drusen (> 63 µm and ≤ 124 µm) or at least 1 large drusen (≥ 125 µm
NonCentral GA	H35.31X3	advanced atrophic dry AMD without subfoveal involvement—geographic atrophy (GA) not involving the center of the fovea
Central GA	H35.31X4	advanced atrophic dry AMD with subfoveal involvement—GA involving the center of the fovea
Unspecified	H35.31X0, H35.31,H35.312,H35.311,H35.313	

Diagnostics from the matching indicated a good overlap of propensity scores between the two groups (see appendix).

Eyes were considered to have “converted” to wet AMD during the follow-up period if both of the following occurred:

- an ICD code for wet AMD was entered into the database
- anti-VEGF injections were initiated

The time of wet AMD conversion was the earliest of the date where the ICD code was entered or the date of the first anti-VEGF injection.

Baseline Tabulations, VPT Non-Laser Excluded, with Encounter Matching

The following Table summarizes the demographics of the two Groups in this analysis.

Table 3. Demographics by study group, after propensity score matching.

**Factor**<br>Level	VPT	SCA
**N (study eyes)**	737	7370
**N (subjects)**	406	4652
**Gender**		
Female	249/406 (61.3%)	2738/4652 (58.9%)
Male	157/406 (38.7%)	1858/4652 (39.9%)
Other	0/406 (0.0%)	56/4652 (1.2%)
**Age (years)**		
Mean(SD)	77.7 (9.1)	77.8 (9.2)
Median	78.0	78.0
Min, Max	[54.0, 93.0]	[50.0, 93.0]
**Age (category)**		
Age: [50,65]	32/406 (7.9%)	442/4652 (9.5%)
Age: (65,70]	56/406 (13.8%)	599/4652 (12.9%)
Age: (70,75]	77/406 (19.0%)	784/4652 (16.9%)
Age: (75,80]	79/406 (19.5%)	971/4652 (20.9%)
Age: (80,85]	72/406 (17.7%)	820/4652 (17.6%)
Age: (85,90]	53/406 (13.1%)	552/4652 (11.9%)
Age: (90,110]	37/406 (9.1%)	484/4652 (10.4%)
**Hypertension**		
No	185/406 (45.6%)	2139/4652 (46.0%)
Yes	221/406 (54.4%)	2513/4652 (54.0%)
**AREDS use**		
No	206/406 (50.7%)	2463/4652 (52.9%)
Yes	200/406 (49.3%)	2189/4652 (47.1%)
**Smoking**		
No	393/406 (96.8%)	4512/4652 (97.0%)
Yes	13/406 (3.2%)	140/4652 (3.0%)
**AMD Severity**		
Early	91/406 (22.4%)	988/4652 (21.2%)
Unspecified	23/406 (5.7%)	273/4652 (5.9%)
Intermediate	235/406 (57.9%)	2621/4652 (56.3%)
NonCentralGA	41/406 (10.1%)	368/4652 (7.9%)
CentralGA	39/406 (9.6%)	454/4652 (9.8%)

Treatment and Raw Outcomes, VPT Non-Laser Excluded, with Encounter Matching

The following Table summarizes followup and AMD treatments received.

Table 4. Follow-up and treatment summary by study group, after propensity score matching.

**Factor**<br>Level	VPT	SCA
**N (study eyes)**	737	7370
**Total Follow-up Days**		
Mean(SD)	677.0 (581.8)	542.6 (633.2)
Median	497.0	232.5
Min, Max	[4.0, 2248.0]	[0.0, 2392.0]
**Follow Up Years (categories)**		
0 ≤ Follow Up Yrs ≤ 1	295/737 (40.0%)	4834/7370 (65.6%)
1 < Follow Up Yrs ≤ 2	175/737 (23.7%)	1054/7370 (14.3%)
Follow Up Yrs > 2	252/737 (34.2%)	1265/7370 (17.2%)
**Number of Encounters**		
Mean(SD)	13.2 (11.0)	11.4 (11.9)
Median	10.0	6.0
Min, Max	[2.0, 53.0]	[2.0, 84.0]
**Number of anti-VEGF injections per eye**		
Mean(SD)	0.138 (0.834)	2.449 (6.976)
Median	0.000	0.000
Min, Max	[0.000, 11.000]	[0.000, 55.000]
**Treated with SDM Laser**		
No	0/737 (0.0%)	N/A
Yes	737/737 (100.0%)	N/A
**Number of Laser Treatments**		
Mean(SD)	6.3 (5.0)	N/A
n	737	N/A
Min, Median, Max	1, 5.0, 38	N/A
**Converted to wAMD**		N/A
Yes	32/737 (4.3%)	1202/7370 (16.3%)
No	705/737 (95.7%)	6168/7370 (83.7%)

There were a total of 32 eyes (4.3%) in 26 subjects in the VPT group, and 1202 eyes (16.3%) in 976 subjects in the SCA group that converted to wet AMD. The following Table summarizes the anti-VEGF treatments received by those eyes after conversion.

Table 5. Follow-up and treatment summary after wet AMD conversion, by study group.

**Factor**<br>Level	VPT	SCA
**N (eyes converted to wAMD)**	32	1202
**wAMD Follow up Days per Eye**		
Mean(SD)	413.8 (376.1)	809.0 (539.8)
Median	298.5	743.5
Min, Max	[0.0, 1270.0]	[0.0, 2334.0]
**Number of anti-VEGF Injections per wAMD Eye**		
Mean(SD)	3.2 (2.5)	14.7 (10.7)
Median	2.0	12.0
Min, Max	[1.0, 11.0]	[1.0, 55.0]

Survival analysis by PS Stratum, VPT Non-Laser Excluded, with Encounter Matching

For conversion to wet AMD, the most appropriate method of analysis appears to be survival analysis using the initial diagnosis of dry AMD as time 0, and conversion to wet AMD as the outcome.

Note that other analysis methods were also carried out, but are not reported here (see the appendix for Poisson regression results). These alternative simpler methods produced the same general conclusions as the survival analysis.

The survival analysis was stratified by propensity score quintiles. That is, eyes were divided into five (nearly equal size) groups using the quintiles of the propensity scores.

The following plots show the cumulative wet AMD conversion by propensity score stratum.

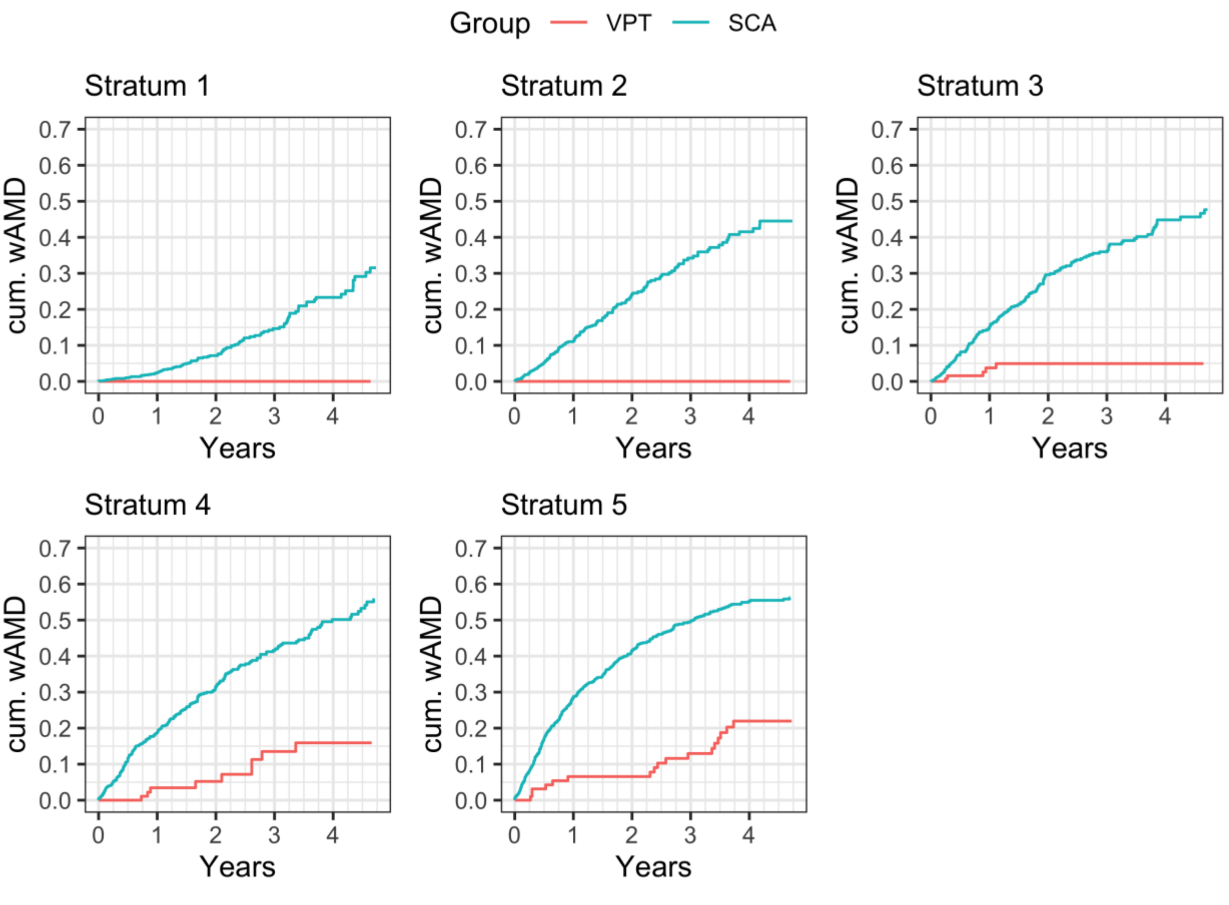

Figure 1. Cumulative probability of wAMD conversion by propensity score strata, VPT Non-Laser Excluded, with Encounter Matching.

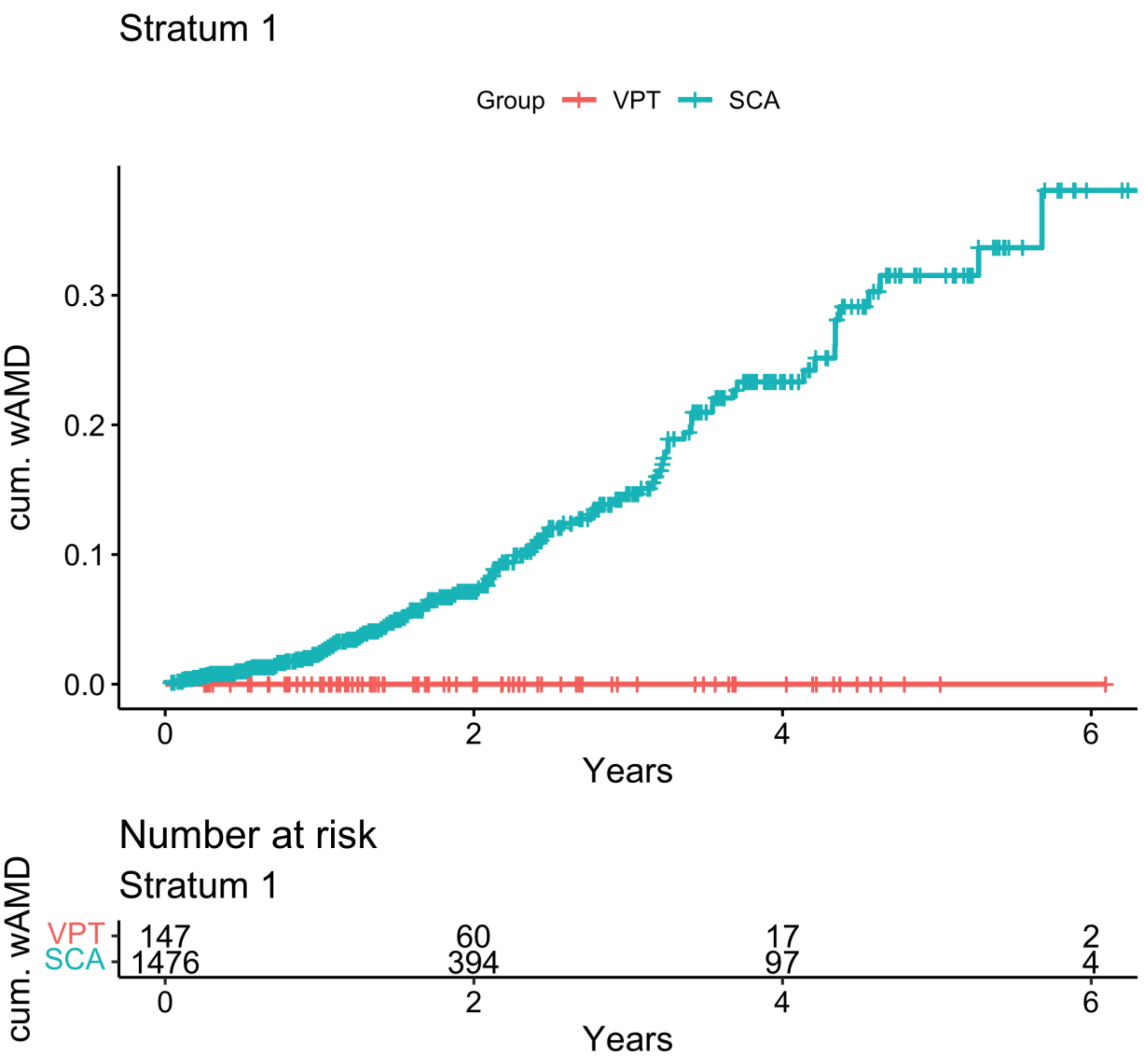
Stratum 1
Group VPT SCA
cum. wAMD
0.0
0.1
0.2
0.3
0
2
4
6
Years
Number at risk
Stratum 1
cum. wAMD
VPT 147 60 17 2
SCA 1476 394 97 4
0
2
4
6
Years

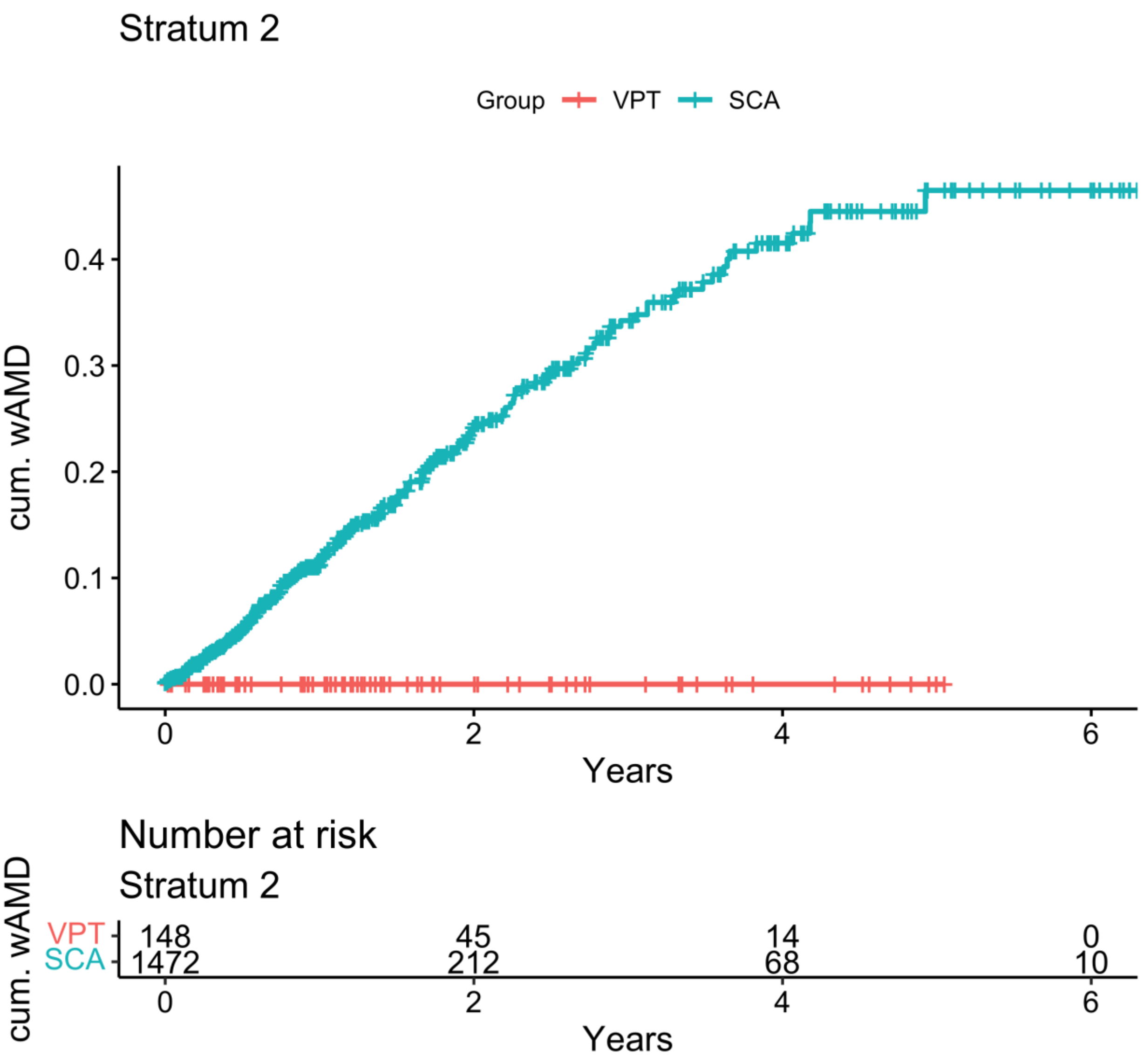
Stratum 2
Group VPT SCA
0.4
0.3
0.2
0.1
0.0
cum. wAMD
0
2
4
6
Years
Number at risk
Stratum 2
cum. wAMD
VPT 148 45 14 0
SCA 1472 212 68 10
0
2
4
6
Years

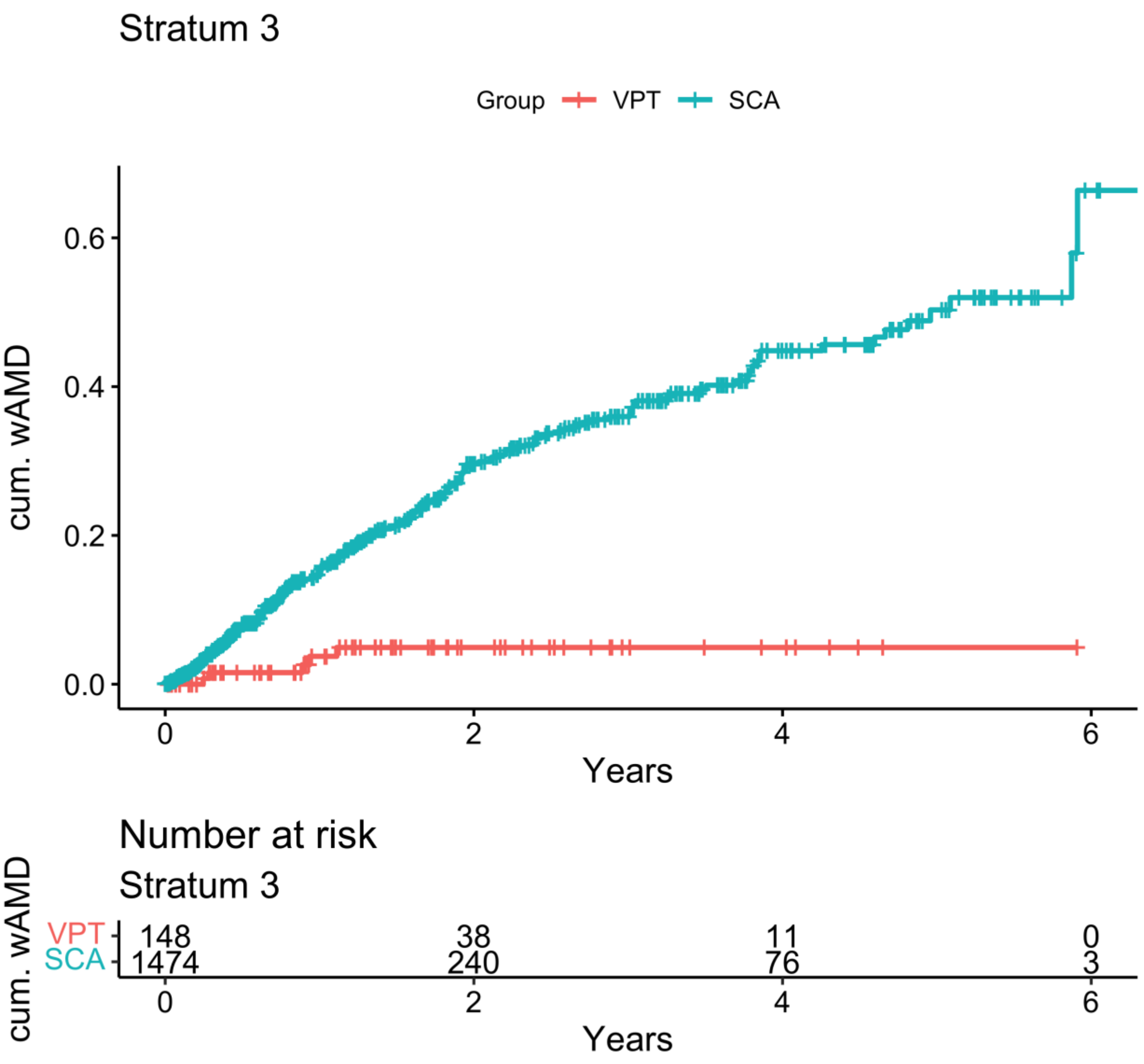
Stratum 3
Group VPT SCA
0.6
0.4
0.2
0.0
cum. wAMD
0
2
4
6
Years
Number at risk
Stratum 3
cum. wAMD
VPT 148 38 11 0
SCA 1474 240 76 3
0
2
4
6
Years

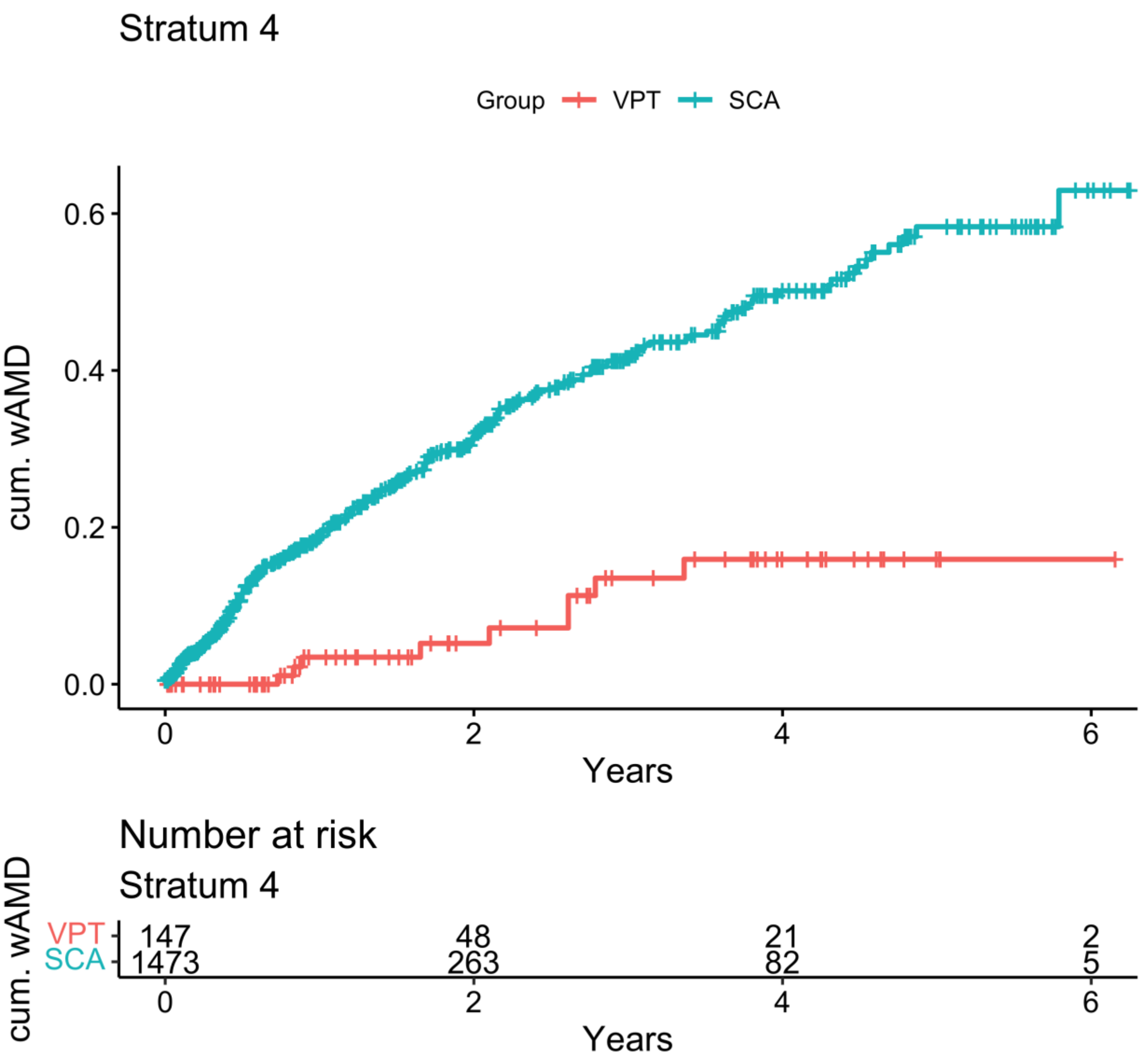
Stratum 4
Group VPT SCA
cum. wAMD
0.0
0.2
0.4
0.6
0
2
4
6
Years
Number at risk
Stratum 4
cum. wAMD
VPT 147 48 21 2
SCA 1473 263 82 5
0
2
4
6
Years

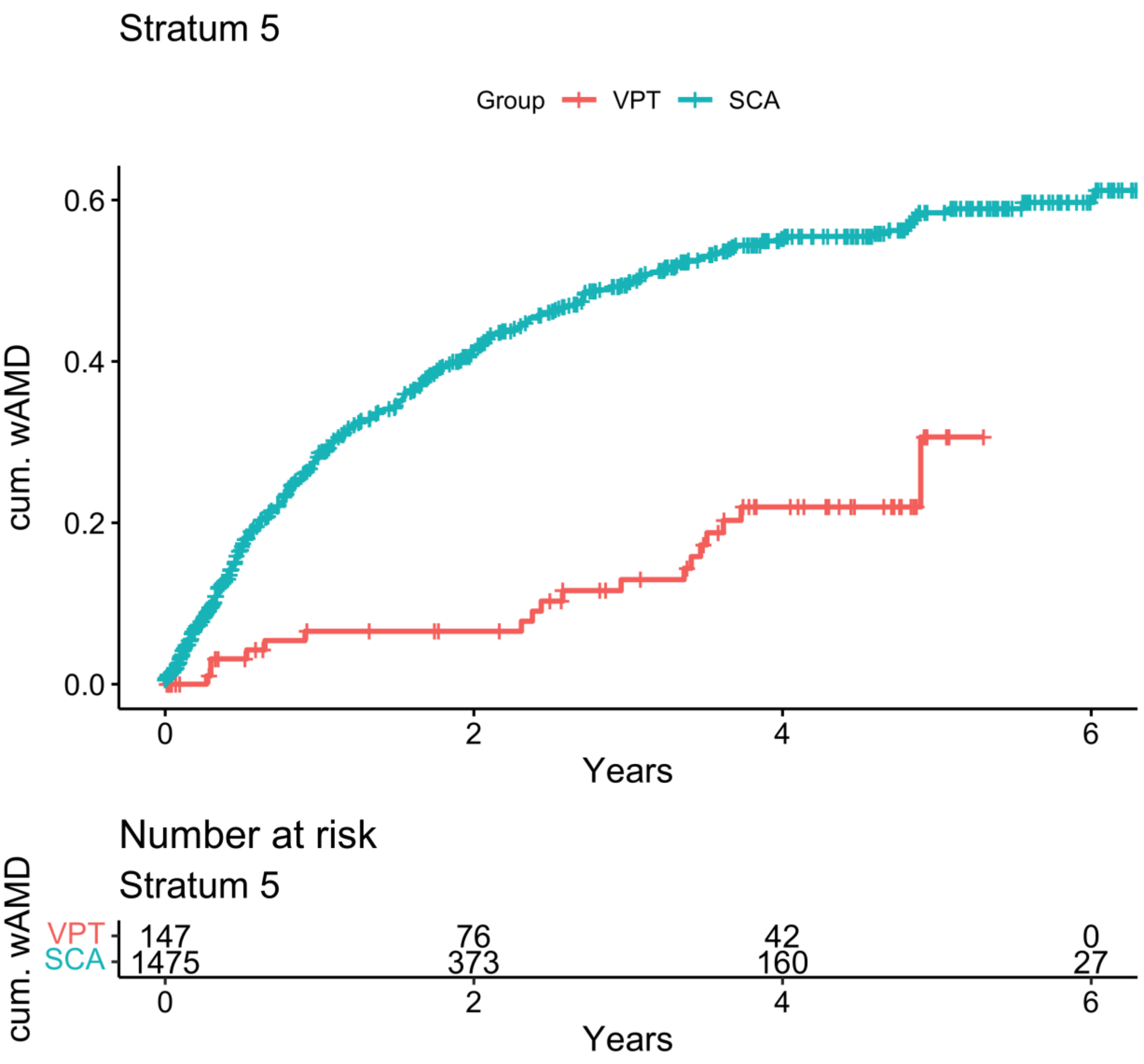
Stratum 5
Group VPT SCA
cum. wAMD
0.0
0.2
0.4
0.6
0
2
4
6
Years
Number at risk
Stratum 5
cum. wAMD
VPT 147 76 42 0
SCA 1475 373 160 27
0
2
4
6
Years

A test for equality of the Kaplan-Meier survival curves (a stratified log-rank test) shows a very significant difference in survival between the VPT and SCA groups.

Table 6. Kaplan-Meier test between Groups, stratified by propensity score quintiles. Chisq = 131.397286 on 1 degrees of freedom, p = 0.000000

	N	Observed	Expected	(O-E)^2/E	(O-E)^2/V
**Analysis.Group=VPT**	737	32	170.1	112.1	131.4
**Analysis.Group=SCA**	7370	1202	1064	17.93	131.4

To allow for inclusion of covariates, and to provide an overall summary of the results, we carried out a Cox proportional hazards regression, again stratified by propensity score quintiles.

Cox PH modelinig initially included all of the covariates included in the propensity score calculation. Non-significant variables were dropped, until the final model included the following important covariates:

- Severity
- Age
- AREDS use
- Analysis Group

Results from the Cox proportional hazards fit are shown in the following Table.

Table 7. Cox PH summary of survival difference, VPT Non-Laser Excluded, with Encounter Matching. Estimated hazard ratios are in the column labeled exp(coef).

	coef	exp(coef)	se(coef)	z	p
**SeverityUnspecified**	0.6335	1.884	0.1404	4.513	6.405e-06
**SeverityIntermediate**	0.5057	1.658	0.1036	4.881	1.057e-06
**SeverityNonCentralGA**	0.6886	1.991	0.1348	5.109	3.23e-07
**SeverityCentralGA**	0.1946	1.215	0.1448	1.343	0.1791
**Age**	0.01495	1.015	0.003706	4.035	5.457e-05
**Areds.FlagYes**	0.3989	1.49	0.06654	5.994	2.045e-09
**Analysis.GroupSCA**	1.788	5.978	0.1794	9.968	0

Likelihood ratio test=295.58 on 7 df, p=0 n= 8107, number of events= 1234

For severity, the "Early" group (used as the reference group in the CPH model) had the highest hazard, with all of the other severity levels producing a hazard ratio < 1. Depending on which of the 4 analysis data sets were used, several other severity levels showed significant increase in hazard over the reference group.

Subjects with AREDS use showed a significant increase in hazard (30%-50%, depending on analysis data set).

For all analysis data sets, there was a significant increase in hazard for the SCA group, of about 5.75 after adjusting for severity, age, and AREDS use (here we have (HR = 5.98, p = <1e-04)).

The following forest plot shows the parameter estimates and confidence intervals from the Cox PH regression.

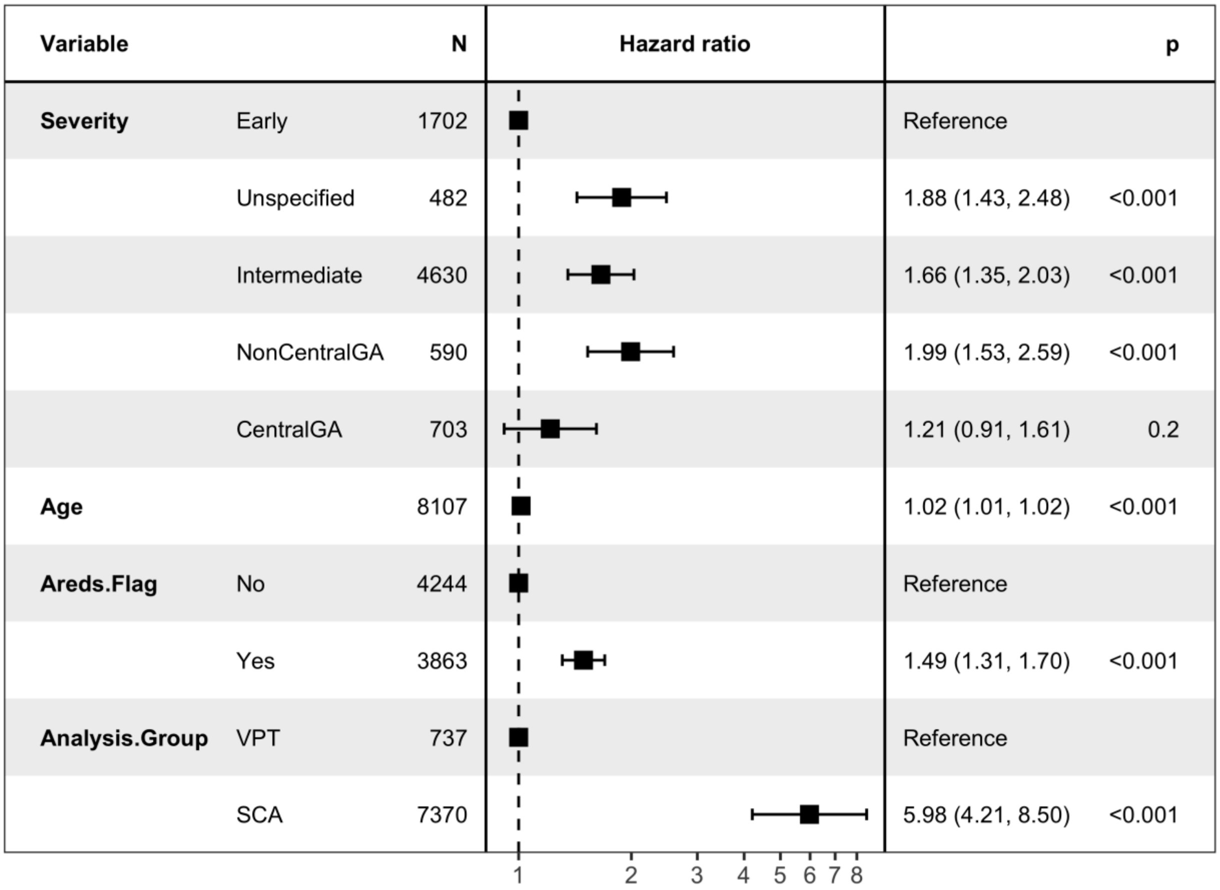

Figure 2. Forest plot of Cox PH results, VPT Non-Laser Excluded, with Encounter Matching

The test for Analysis Group shows a high level of significance.

Table 8. ANOVA for Cox PH model, VPT Non-Laser Excluded, with Encounter Matching.

	loglik	Chisq	Df	Pr(>|Chi|)
**NULL**	-7745	NA	NA	NA
**Severity**	-7717	57.11	4	1.174e-11
**Age**	-7709	15.14	1	9.958e-05
**Areds.Flag**	-7689	39.87	1	2.714e-10
**Analysis.Group**	-7598	183.5	1	8.524e-42

A test for the proportional hazards assumption shows strong evidence of non-proportionality (cox.zph(), p = 2.23e-05). However (see appendix), various diagnostic plots do not indicate strong non-proportionality in the Cox PH model.

Table 9. Tests for proportional hazards violations., VPT Non-Laser Excluded, with Encounter Matching.

	chisq	df	p
**Severity**	21.91	4	0.0002086
**Age**	0.1243	1	0.7244
**Areds.Flag**	3.604	1	0.05766
**Analysis.Group**	7.135	1	0.007559
**GLOBAL**	33.4	7	2.232e-05

Summary of Survival Fits, VPT Non-Laser Excluded, with Encounter Matching

The following plot shows the overall cumulative wet AMD conversion probabilities by group, ignoring covariates.

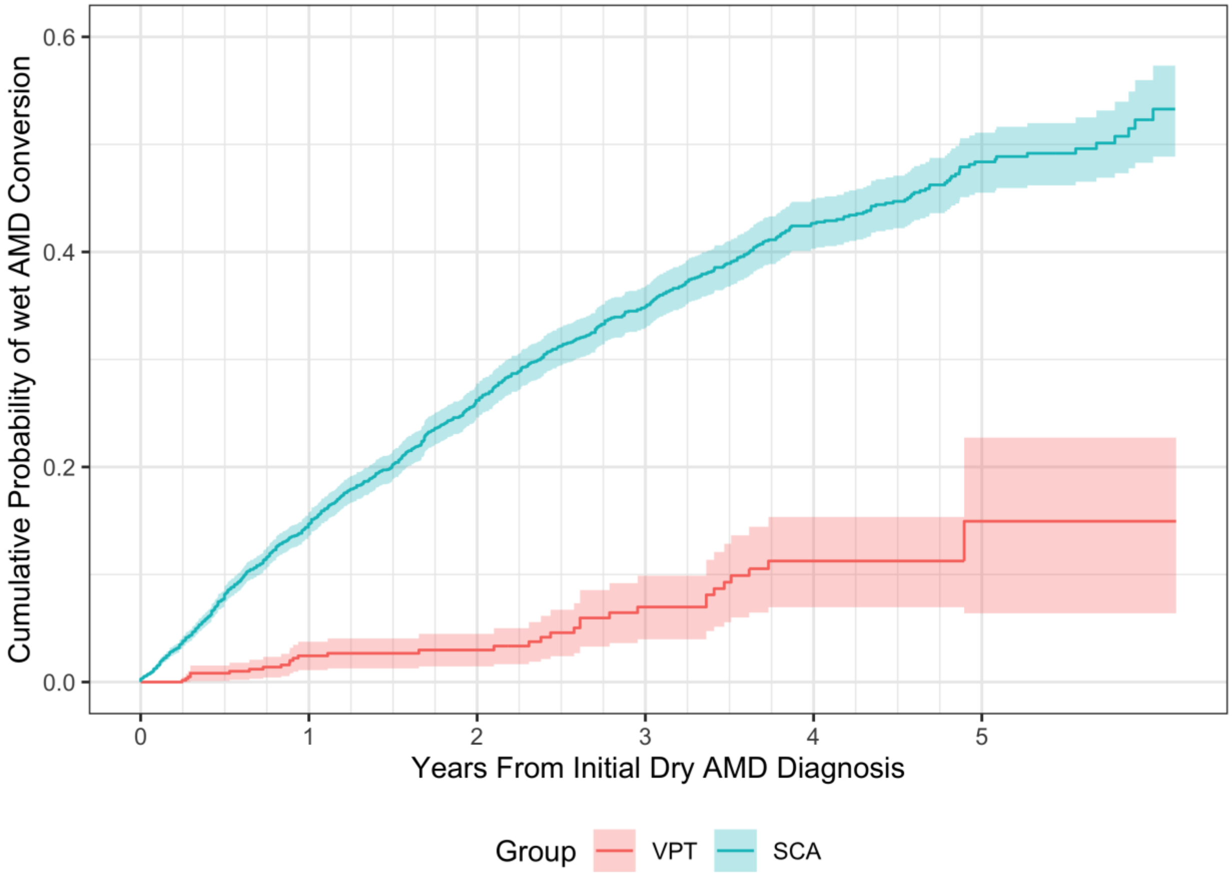

Figure 3. Overall Kaplan-Meier cumulative wet AMD conversion probability by group, VPT Non-Laser Excluded, with Encounter Matching. Shaded areas indicate 95% confidence intervals.

The following Table shows the cumulative probability of progressing to wet AMD, by year and group.

Table 10. Summary of overall survival by group (unstratified Kaplan-Meier estimates), VPT Non-Laser Excluded, with Encounter Matching.

Analysis Group	Years From DAMD Diagnosis	n at risk	n events	Cumulative Probability of wet AMD	95% CI
VPT	1	442	13	2.4%	[1.1%, 3.7%]
	2	267	2	3.0%	[1.5%, 4.5%]
	3	175	9	7.0%	[4.0%, 9.9%]
	4	105	7	11.3%	[7.0%, 15.4%]
SCA	1	2536	641	14.7%	[13.6%, 15.8%]
	2	1482	283	26.2%	[24.6%, 27.8%]
	3	884	148	34.9%	[32.9%, 36.8%]
	4	483	87	42.7%	[40.3%, 44.9%]

The hazard ratio between the two groups is summarized in the following Table. Since there are multiple eyes per person, a clustered bootstrap (clustered by subject) was used to provide a robust check on the confidence interval. The lower bound on the 95% confidence interval for the hazard ratio is above 4 using either method, again providing strong evidence for a hazard ratio greater than 1.

Table 11. Cox proportional hazards estimated hazard ratio and associated confidence intervals, VPT Non-Laser Excluded, with Encounter Matching. Cox PH model is stratified by propensity score quartiles.

Estimated Hazard Ratio	95% CI (asymptotic)	95% CI (bootstrap[1])
6.0	[4.2, 8.5]	[4.3, 9.3]

1-Bootstrap confidence interval is based on 10000 cluster (subject level) bootstrap samples.

Visual Acuity, VPT Non-Laser Excluded, with Encounter Matching

Visual acuity (ETDRS letters or the equivalent) was measured for a subset of subject visits (usually non-treatment visits). The SCA group averaged about 619.1 VA measurements per month, the VPT group averaged 50.8.

A tabulation of the mean VA by year shows a slight downward trend for the SCA group (perhaps due to aging?) but no obvious differences between the two groups.

Table 12. Mean visual acuity (ETDRS letters or equivalent) by Group and Year, VPT Non-Laser Excluded, with Encounter Matching. Cox PH model is stratified by propensity score quartiles.

Analysis Group	Mean VA 2017	Mean VA 2018	Mean VA 2019	Mean VA 2020	Mean VA 2021	Mean VA 2022
VPT	71.2	67.0	68.1	70.4	67.9	66.9
SCA	66.7	66.9	65.5	65.1	63.9	63.5

The following plot shows the mean VA per month for the SCA and VPT subjects. As expected there is more noise in the much smaller VPT group. Given the amount of noise it is difficult to assess whether there are any differences in VA through time.

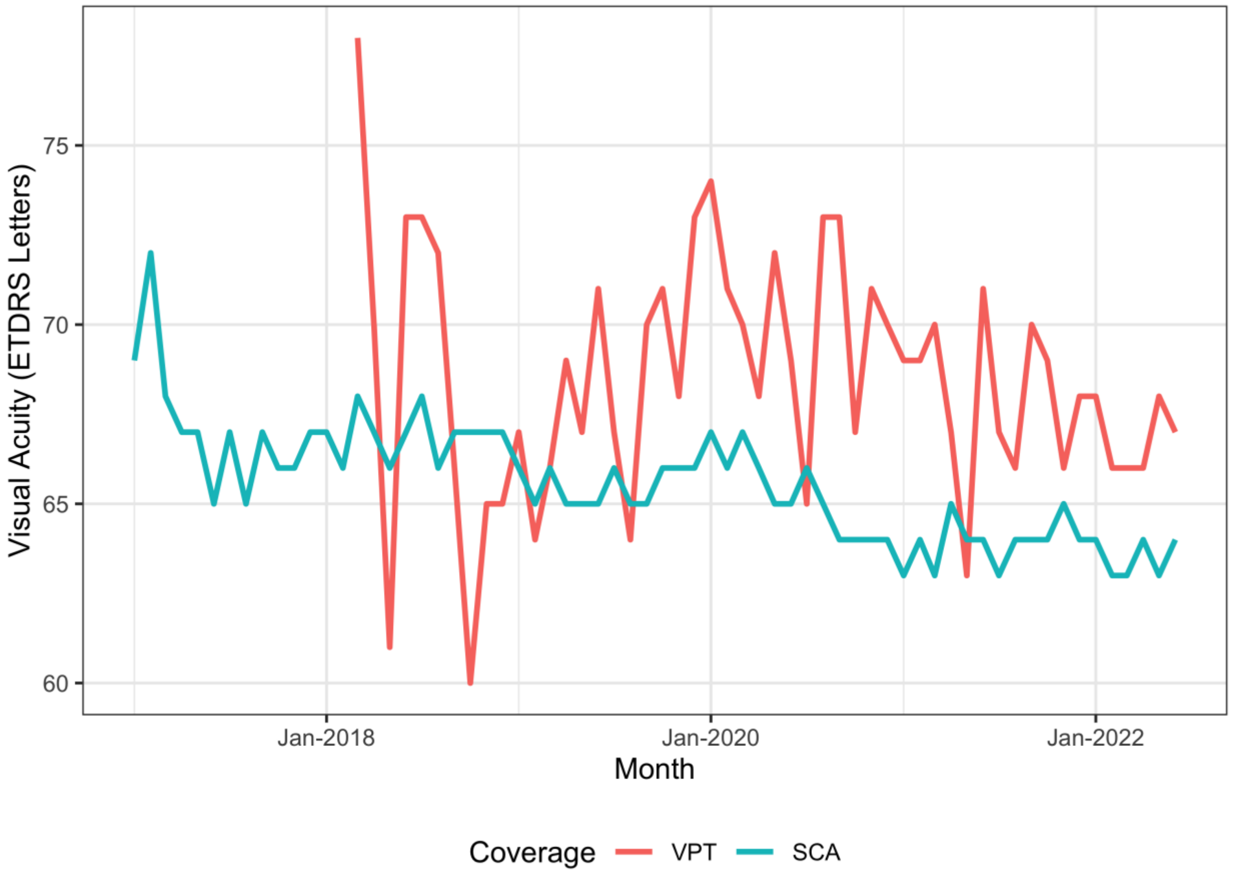

Figure 4. Visual Acuity by month, SCA, VPT Groups, VPT Non-Laser Excluded, with Encounter Matching.

A loess smoother shows a difference between the two groups during 2020-2021, perhaps due to the COVID pandemic?

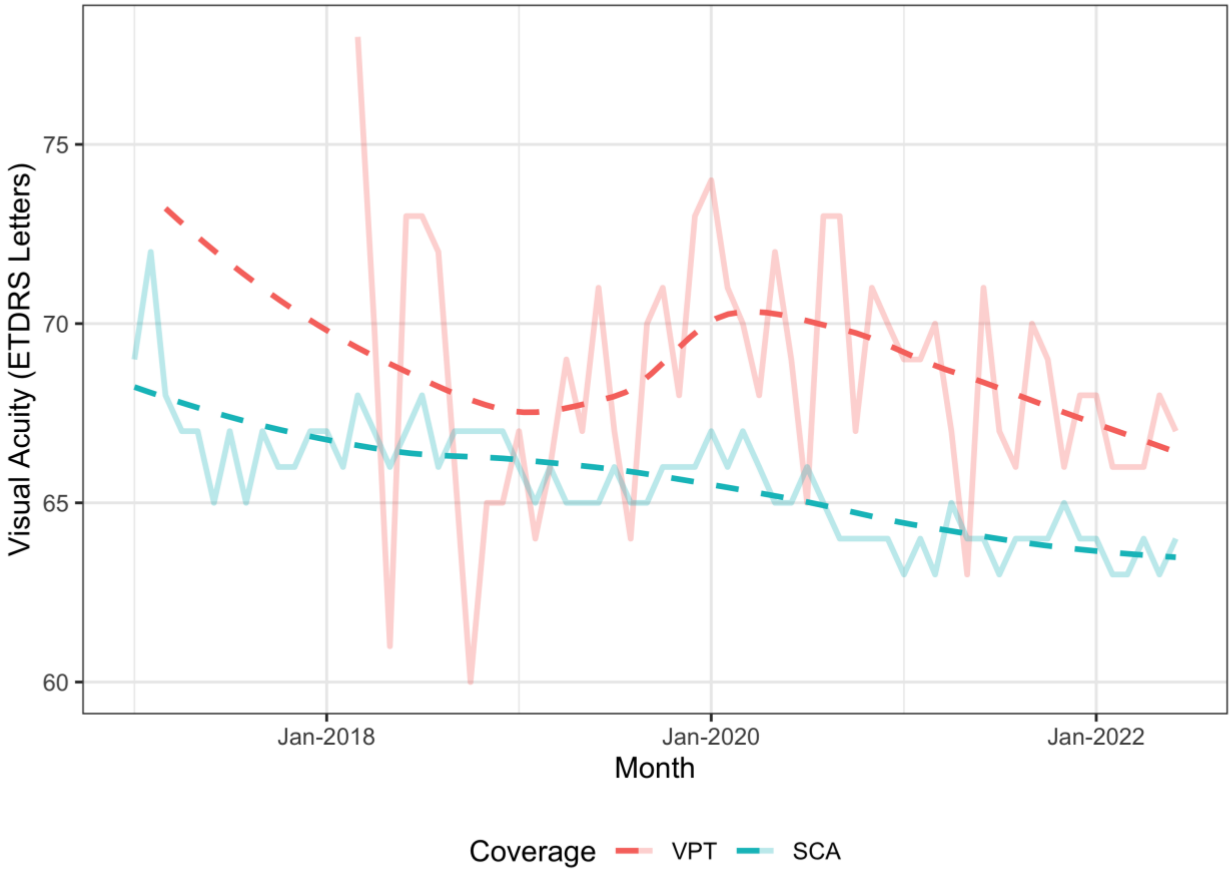

Figure 5. Visual Acuity by month, SCA, VPT Groups, with loess smooths., VPT Non-Laser Excluded, with Encounter Matching.

Caveats for Propensity Score Analysis

Potential shortcomings of this propensity score analysis include:

- The VPT group only includes subjects from a single practice, thus the generalizability of the results may be in question.
- Propensity score methods can "balance" the two groups with respect to the variables used in the propensity score model. However, they do not balance for unmeasured covariates, thus if there are other important predictors of group membership or outcome that are missing the results could be misleading.
- This analysis used subjects for whom the latest ICD-10 coding was used, which added Dry AMD severity codes not present in the earlier ICD-9. We are thus using the site-level assessment of AMD severity and assume a reasonably consistent diagnostic judgement across sites. There is no reading center or verification of the coding as would be implemented in a clinical trial.
- The ICD-10 coding used in the Vestrum database may not capture all subject outcomes, including potentially some adverse events.
- There are inconsistencies in ICD coding, for example some subjects who had a series of anti-VEGF injections were never coded as having converted to wet AMD. Thus an eye was considered converted to wet AMD if the appropriate ICD code was entered **and** at least one anti-VEGF injection was administered. The conversion date was set to earliest of the ICD coding date or the date of the first anti-VEGF injection. Alternative definitions of conversion do not change the basic conclusions.
- There are differences in follow-up time and number of encounters (reported subject-physician interactions) between the two groups, with the VPT group having more encounters and longer follow-up. As these are "post-randomization" outcomes they were not used in the initial propensity score matching. The longer follow-up in the VPT group most likely produced more events, whereas the effect of increased encounters, if any, is unknown. Thus if there is any bias due to follow-up it is likely to produce a smaller hazard ratio for SCA versus VPT.
- To match follow-up intensity between the two groups a second propensity score analysis used the mean time-between-visits for each subject as an additonal matching variable. This produces a better correspondence in follow-up time and number of visits between the two groups at the expense of potential bias issues with using post-diagnosis information in the matching.

Appendix/Supplemental

This appendix includes diagnostic plots and alternative analyses.

Distribution of Follow-Up Time

The following plot shows the follow-up time by group and wet AMD status.

Note the spikes in the SCA group at approximately 3 months, 6 months, 1 year, 2 years, and 3 years of follow-up.

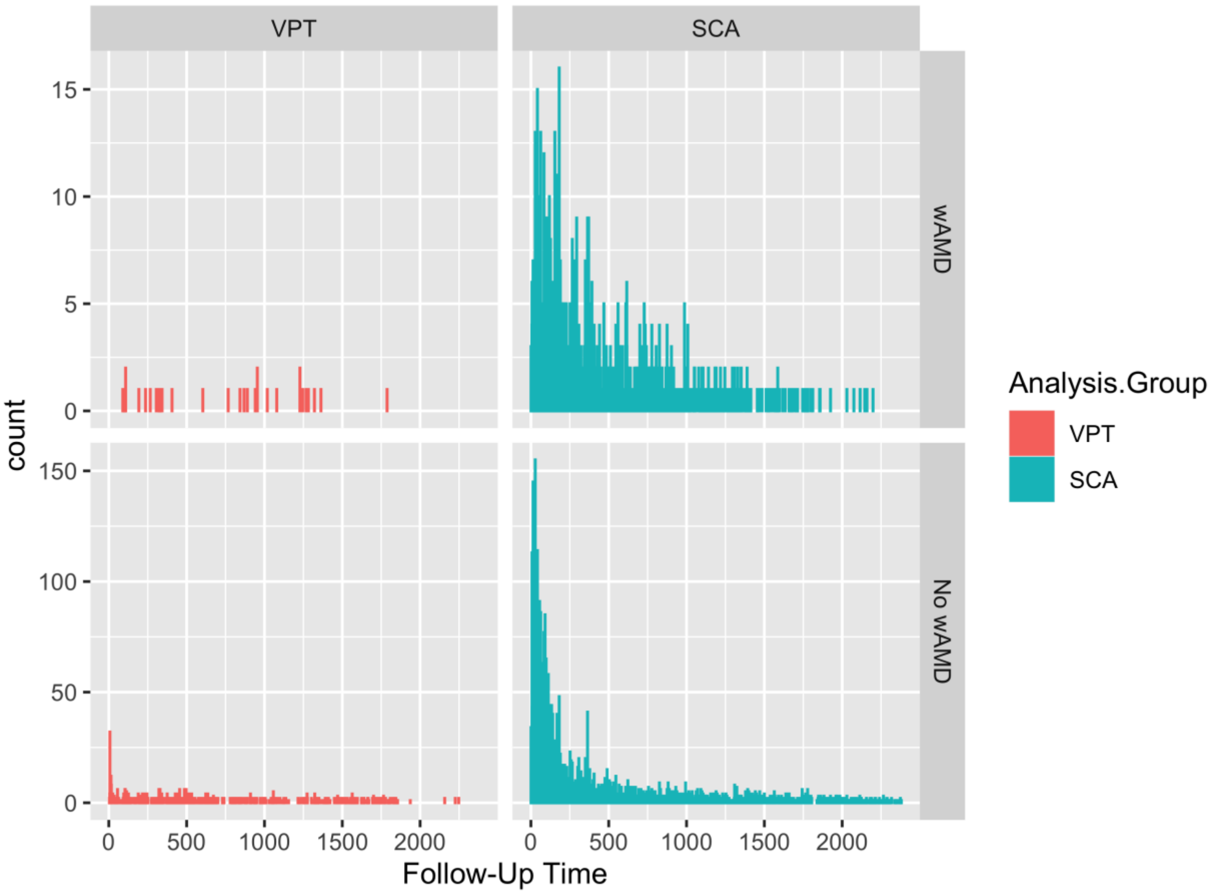

Figure 6. Follow up time by study group and wAMD conversion status. Subjects with 0 days follow-up are excluded.

Diagnostic Plots for Propensity Scores

Propensity score diagnostic plots look good overall. The overall distributions for the propensity scores are quite similar.

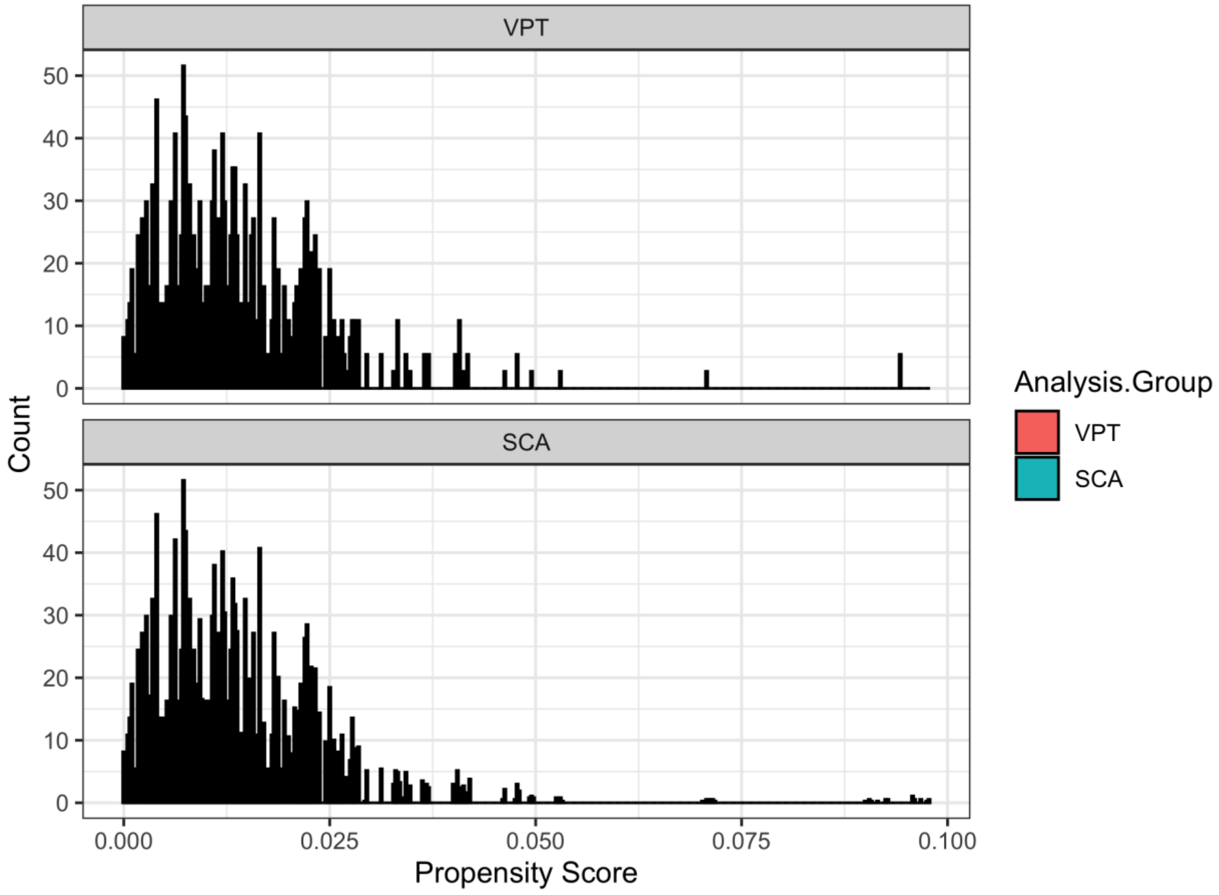

Figure 7. Diagnostic plots for propensity scores, overall distribution.

Propensity scores are also similar within each PS stratum.

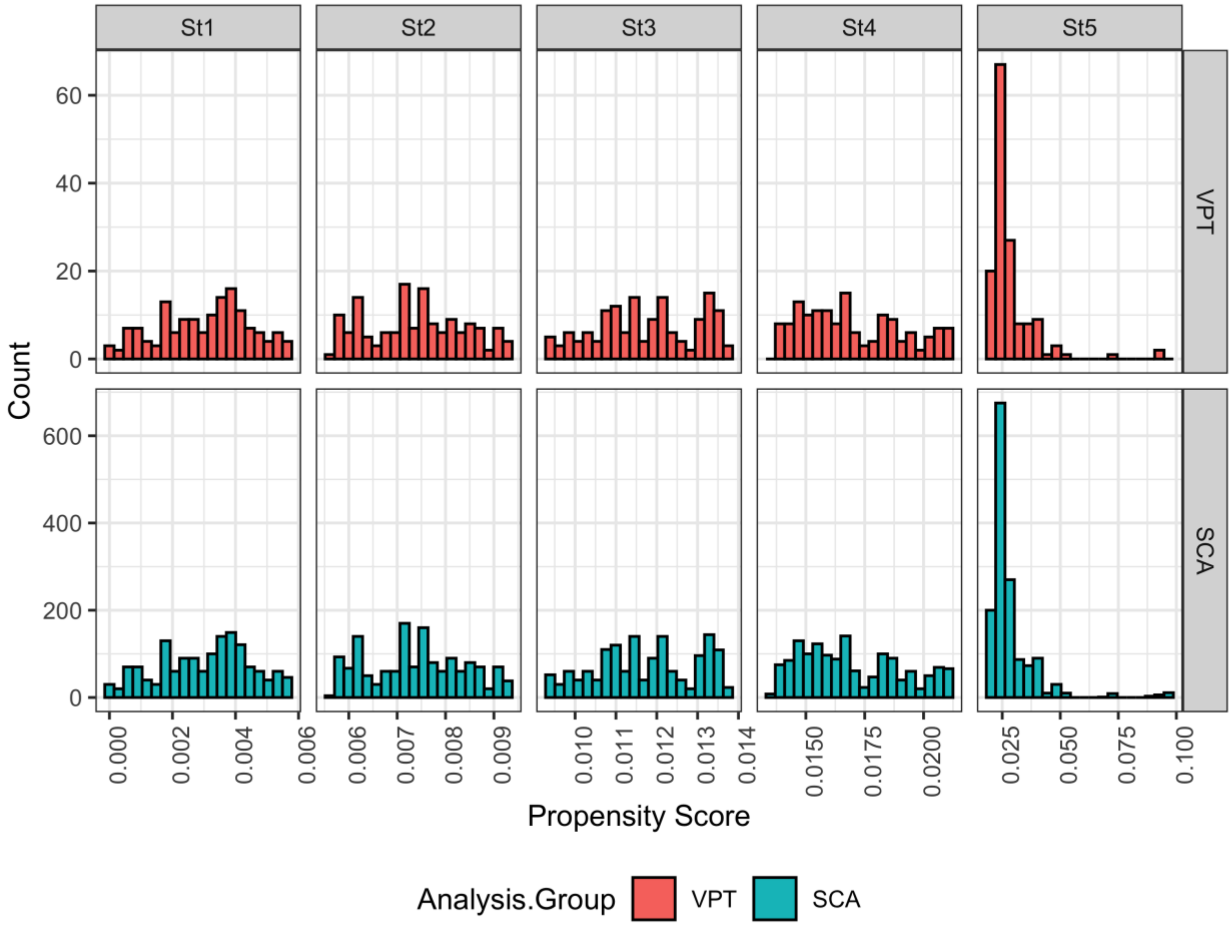

Figure 8. Diagnostic plots for propensity scores, distribution by PS stratum.

Each individual component also looks good. What we want to see is that within each stratum the two groups are similar (e.g. the pairs of orange and yellow bars have good overlap, the pairs of green bars are similar height).

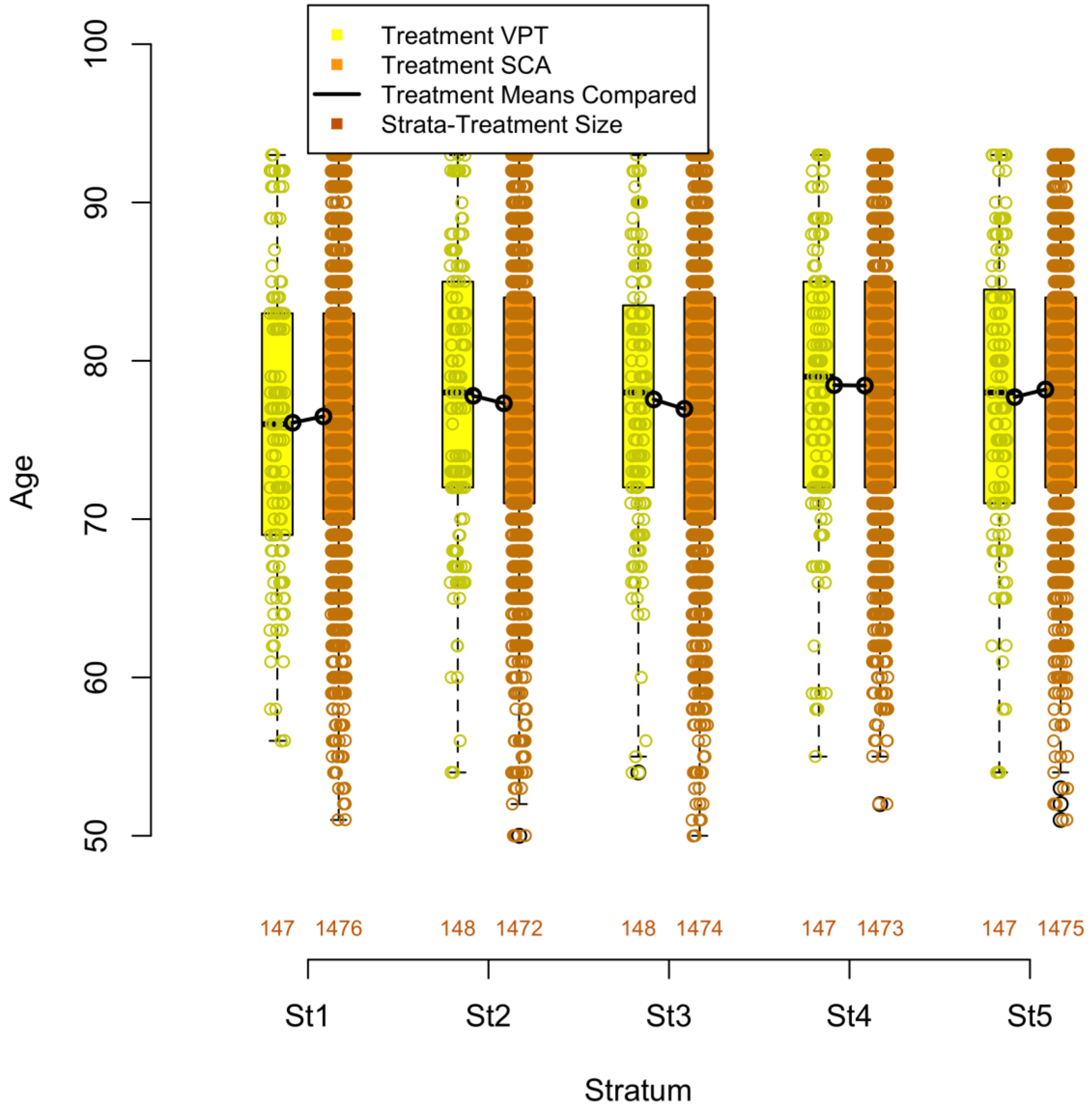

Figure 9. Diagnostic plots for propensity scores, age.

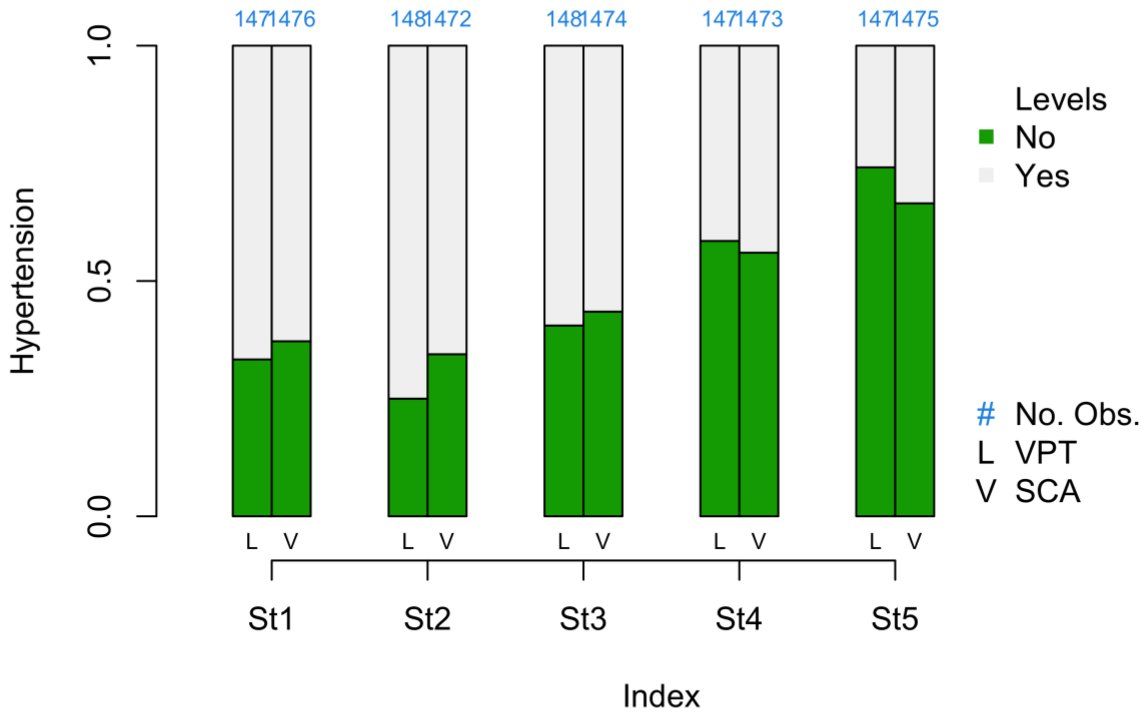

Figure 10. Diagnostic plots for propensity scores, hypertension.

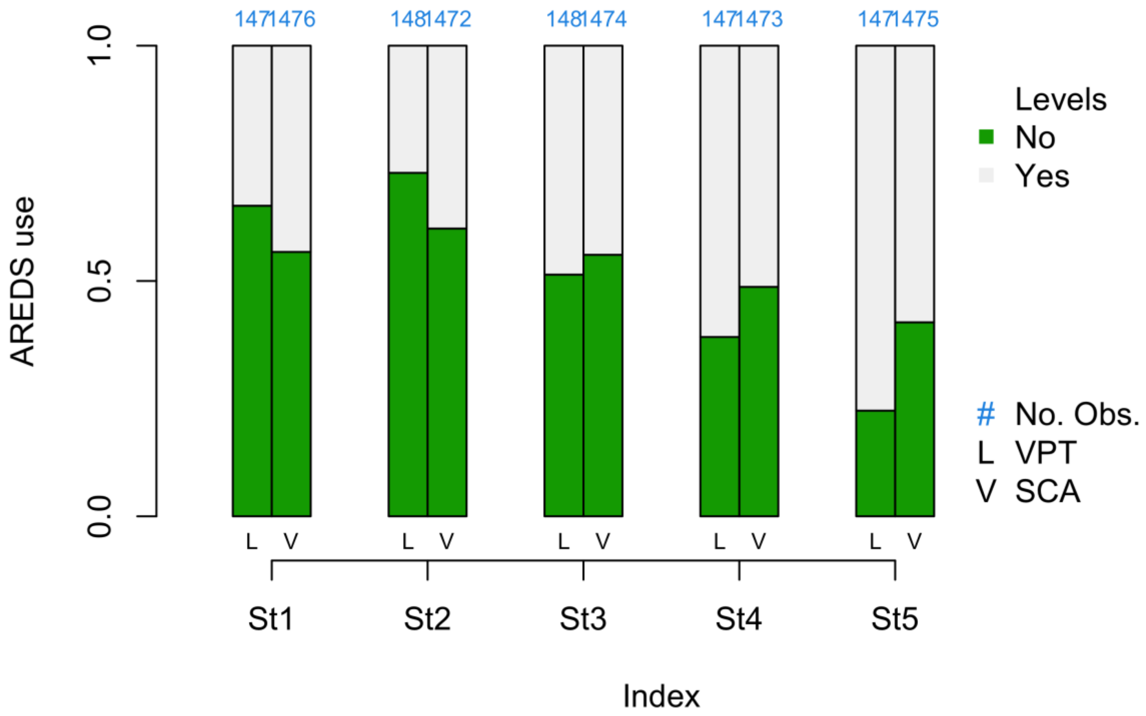

Figure 11. Diagnostic plots for propensity scores, AREDS use.

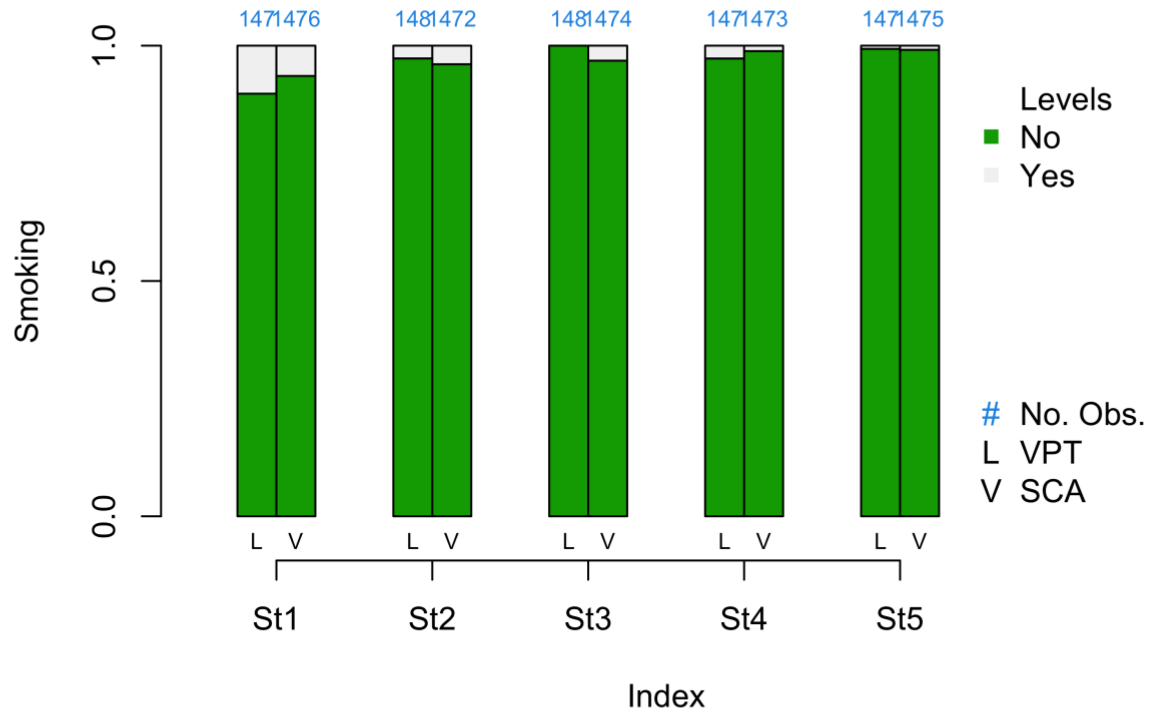

Figure 12. Diagnostic plots for propensity scores, smoking.

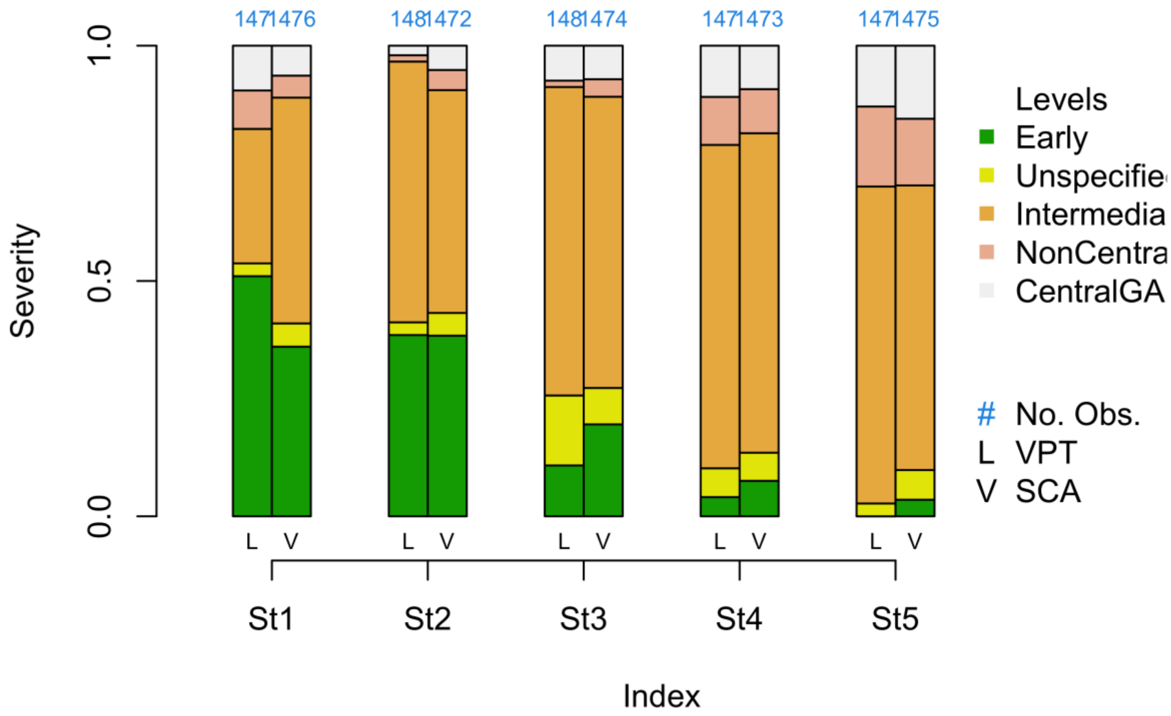

Figure 13. Diagnostic plots for propensity scores, severity

Incidence Rates via Poisson Regression

As a simpler alternative to the survival analysis, we can fit a poisson regression model. Poission regression adjusts for the follow-up on each eye individually, so corrects the bias in incidence rates somewhat. We can also easily account for the propensity score strata.

Fitting generalized (poisson/log) linear model: Converted.to.wAMD.n ~ Analysis.Group + PSStratum + offset(Follow.up.Years)

	Estimate	Std. Error	z value	Pr(>|z|)
**(Intercept)**	-7.174	0.2005	-35.79	1.641e-280
**Analysis.GroupSCA**	1.689	0.1792	9.427	4.232e-21
**PSStratumSt2**	0.87	0.1233	7.056	1.713e-12
**PSStratumSt3**	1.009	0.119	8.483	2.197e-17
**PSStratumSt4**	1.189	0.1149	10.35	4.406e-25
**PSStratumSt5**	0.9029	0.1088	8.302	1.025e-16

Table 13. Incidence rates from poisson regression (correcting for unequal follow-up in data, and adjusting for stratum differences.)

Analysis.Group	incidence rate	std.error	df	null	statistic	p.value
VPT	0.004609	0.0008179	Inf	1	-30.32	7.018e-202
SCA	0.02496	0.0008062	Inf	1	-114.3	0

The incidence rate ratio from this fit is 5.4.

Diagnostic Plots for Cox PH Model

The following plots are diagnostics from the Cox proportional hazards fit.

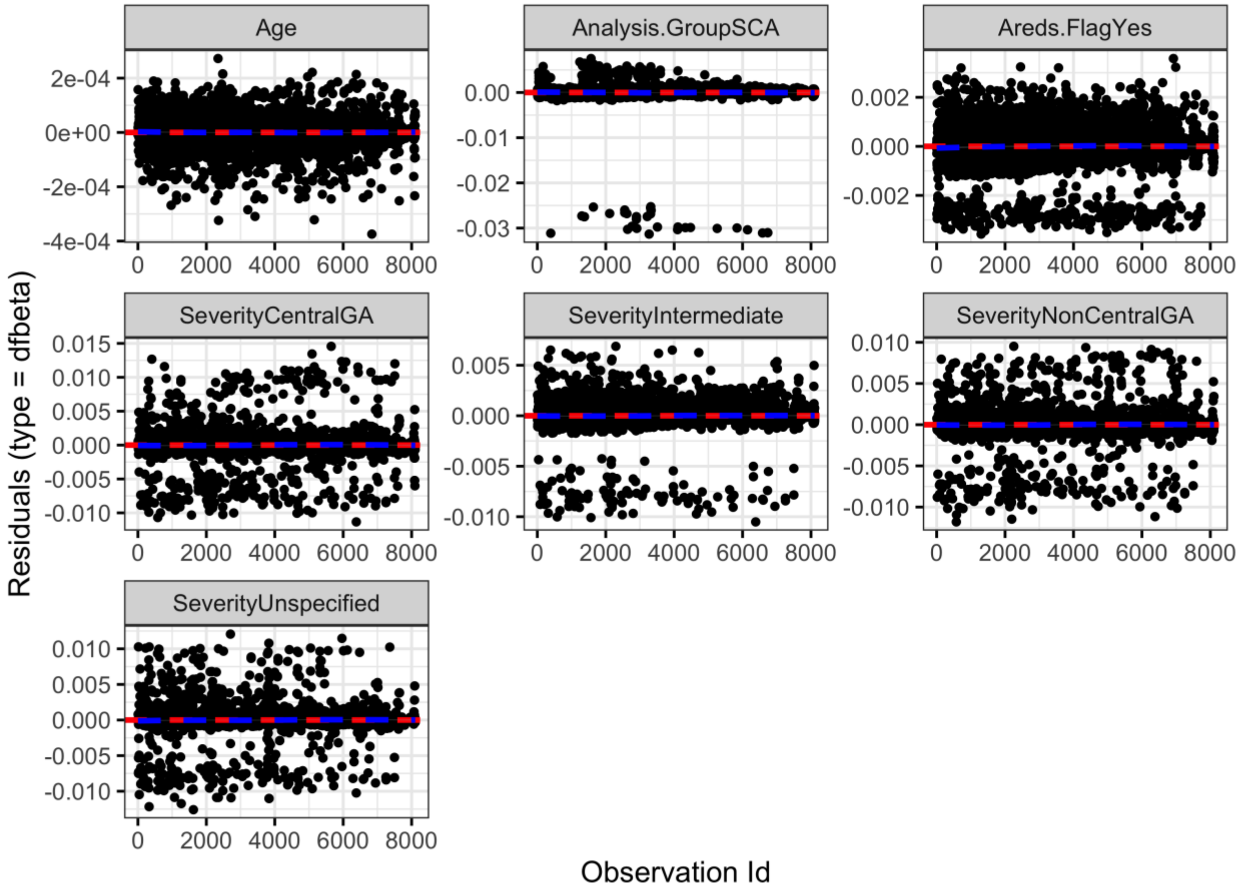

Figure 14. Dfbeta residuals from Cox PH fit.

```
## `geom_smooth()` using formula = 'y ~ x'
```

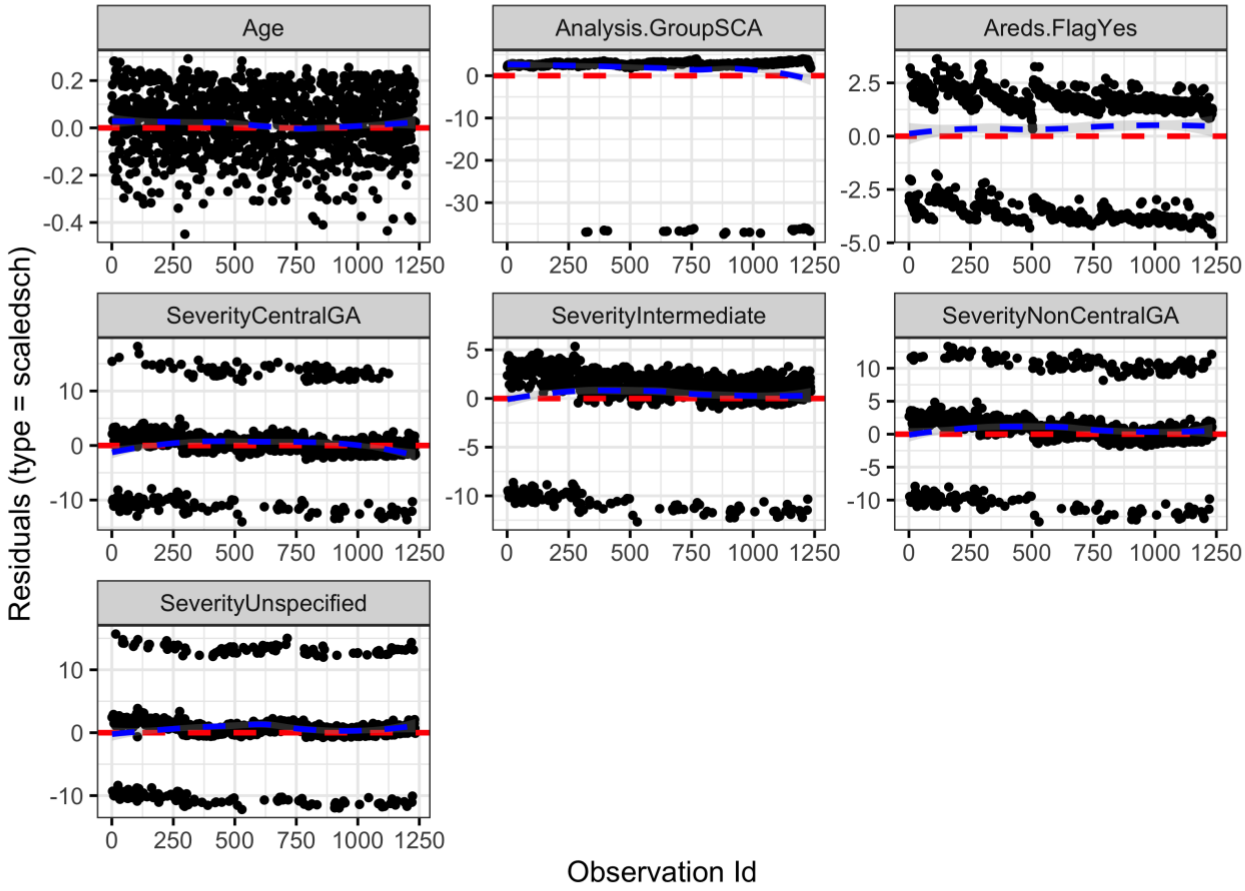

Figure 15. Scaled Schoenfeld residuals vs Time.

Retinal Protection Sciences

Comparison of SDM treatment to standard of care
Propensity matched Vestrum data,
with AMD severity coding
v6.0

[without non-laser-treated VPT subjects]
[without encounter matching]

Gerry Gray, Regulatory Pathways, Inc.

9/20/2023

Table of Contents

INTRODUCTION 34
BASELINE TABULATIONS, VPT NON-LASER EXCLUDED, NO ENCOUNTER MATCHING 36
TREATMENT AND RAW OUTCOMES, VPT NON-LASER EXCLUDED, NO ENCOUNTER MATCHING 37
SURVIVAL ANALYSIS BY PS STRATUM, VPT NON-LASER EXCLUDED, NO ENCOUNTER MATCHING 39
SUMMARY OF SURVIVAL FITS, VPT NON-LASER EXCLUDED, NO ENCOUNTER MATCHING 48
VISUAL ACUITY, VPT NON-LASER EXCLUDED, NO ENCOUNTER MATCHING 49
CAVEATS FOR PROPENSITY SCORE ANALYSIS 53
APPENDIX/SUPPLEMENTAL 54
DISTRIBUTION OF FOLLOW-UP TIME 54
DIAGNOSTIC PLOTS FOR PROPENSITY SCORES 55
INCIDENCE RATES VIA POISSON REGRESSION 62
DIAGNOSTIC PLOTS FOR COX PH MODEL 63

Introduction

This report contains analyses of propensity score matched data from the Vestrum database.

This is the second propensity score analysis from this database. One of the major perceived shortcomings of the previous analysis was that AMD severity at diagnosis was not available in the then-current ICD-9 coding. This issue was addressed in the new ICD-10 codes, which indicate AMD severity at diagnosis. This new information on initial AMD severity was used in the revised propensity score matching.

This analysis includes severity of the initial AMD diagnosis, classified as "Early", "Unspecified", "Intermediate", "Non-Central GA" or "Central GA".

There are four separate versions of this report, created to satisfy a reviewer's request, to adhere to a principle of "no post-randomization exclusions" of subjects, and to evaluate the sensitivity of the analyses to differential follow-up intensity. The four versions are the combinations of the following:

SDM Laser Treatment

3. Including all subjects from the VPT group who met the initial filtering (I/E) criteria.
4. Including only VPT subjects who were treated with the SDM laser (a "post-randomization" event) in addition to meeting the I/E criteria.

Follow-up Intensity

5. Propensity score matching that includes follow-up intensity, using using the mean time between visits.
6. Propensity score matching that does not include follow-up intensity.

Throughout, we use the labels "VPT" to indicate Luttrull subjects, treated with standard of care and SDM laser as appropriate, and "SCA" to indicate the matched eyes from the Vestrum database.

The Vestrum database of ~500,00 eyes with visits between 1/2/2017 and 7/31/2023 were initially filtered using study inclusion/exclusion criteria to obtain a candidate set of ~200,000 eyes, including 814 VPT eyes (737 who were treated with SDM Laser). Although exclusion of VPT eyes based on treatment recieved during the study violates a statisical principle of "no post-randomization exclusions", at the insistence of reviewers we analyzed data both with and without VPT SDM laser treated eyes.

After completion of the initial filtering, and for both 1) all VPT eyes and 2) only SDM laser-treated VPT eyes, nearest-neighbor propensity score matching was used to obtain a matched set of control eyes from the SCA group. The R Matchit package was used for the matching (R version 4.0.2, Matchit version 3.0.2).

Propensity scores were based on the following covariates:

Table 1. Variables used to perform propensity score matching

Variable
Age
Smoking status
AREDS vitamin use status
Hypertension status
AMD Severity

To obtain similar follow-up intensity between the groups, a second propensity score matching used all of the above variables plus the mean time-between-visits. These analyses are labeled “with encounter matching” in the headings.

An earlier analysis of data from the Vestrum database was reported in REF. At the time of that analysis, the Vestrum system used the then-current ICD-9 codes for AMD.

With the transition to ICD-10, the codes for AMD now include separate categories for disease severity.

Table 2. ICD 10 AMD Severity codings.

AMD Severity	ICD Code	Description
Early	H35.31X1	early dry AMD—a combination of multiple small drusen (≤ 63 μm), few intermediate drusen (> 63 μm and ≤ 124 μm), or retinal pigment epithelium (RPE) abnormalities
Intermediate	H35.31X2	intermediate dry AMD—extensive intermediate drusen (> 63 μm and ≤ 124 μm) or at least 1 large drusen (≥ 125 μm
NonCentral GA	H35.31X3	advanced atrophic dry AMD without subfoveal involvement—geographic atrophy (GA) not involving the center of the fovea
Central GA	H35.31X4	advanced atrophic dry AMD with subfoveal involvement—GA involving the center of the fovea
Unspecified	H35.31X0, H35.31,H35.312,H35.311,H35.313	

Diagnostics from the matching indicated a good overlap of propensity scores between the two groups (see appendix).

Eyes were considered to have “converted” to wet AMD during the follow-up period if both of the following occurred:

- an ICD code for wet AMD was entered into the database
- anti-VEGF injections were initiated

The time of wet AMD conversion was the earliest of the date where the ICD code was entered or the date of the first anti-VEGF injection.

Baseline Tabulations, VPT Non-Laser Excluded, no Encounter Matching

The following Table summarizes the demographics of the two Groups in this analysis.

Table 3. Demographics by study group, after propensity score matching.

**Factor**<br>Level	VPT	SCA
**N (study eyes)**	737	7370
**N (subjects)**	406	4661
**Gender**		
Female	249/406 (61.3%)	2793/4661 (59.9%)
Male	157/406 (38.7%)	1825/4661 (39.2%)
Other	0/406 (0.0%)	43/4661 (0.9%)
**Age (years)**		
Mean(SD)	77.7 (9.1)	78.1 (9.1)
Median	78.0	78.0
Min, Max	[54.0, 93.0]	[50.0, 93.0]
**Age (category)**		
Age: [50,65]	32/406 (7.9%)	427/4661 (9.2%)
Age: (65,70]	56/406 (13.8%)	538/4661 (11.5%)
Age: (70,75]	77/406 (19.0%)	796/4661 (17.1%)
Age: (75,80]	79/406 (19.5%)	986/4661 (21.2%)
Age: (80,85]	72/406 (17.7%)	848/4661 (18.2%)
Age: (85,90]	53/406 (13.1%)	562/4661 (12.1%)
Age: (90,110]	37/406 (9.1%)	504/4661 (10.8%)
**Hypertension**		
No	185/406 (45.6%)	2011/4661 (43.1%)
Yes	221/406 (54.4%)	2650/4661 (56.9%)
**AREDS use**		
No	206/406 (50.7%)	2403/4661 (51.6%)
Yes	200/406 (49.3%)	2258/4661 (48.4%)
**Smoking**		
No	393/406 (96.8%)	4525/4661 (97.1%)
Yes	13/406 (3.2%)	136/4661 (2.9%)
**AMD Severity**		
Early	91/406 (22.4%)	933/4661 (20.0%)
Unspecified	23/406 (5.7%)	270/4661 (5.8%)
Intermediate	235/406 (57.9%)	2734/4661 (58.7%)
NonCentralGA	41/406 (10.1%)	350/4661 (7.5%)
CentralGA	39/406 (9.6%)	414/4661 (8.9%)

Treatment and Raw Outcomes, VPT Non-Laser Excluded, no Encounter Matching

The following Table summarizes followup and AMD treatments received.

Table 4. Follow-up and treatment summary by study group, after propensity score matching.

**Factor**<br>Level	VPT	SCA
**N (study eyes)**	737	7370
**Total Follow-up Days**		
Mean(SD)	677.0 (581.8)	429.2 (539.1)
Median	497.0	181.0
Min, Max	[4.0, 2248.0]	[0.0, 2392.0]
**Follow Up Years (categories)**		
0 ≤ Follow Up Yrs ≤ 1	295/737 (40.0%)	5157/7370 (70.0%)
1 < Follow Up Yrs ≤ 2	175/737 (23.7%)	1095/7370 (14.9%)
Follow Up Yrs > 2	252/737 (34.2%)	1008/7370 (13.7%)
**Number of Encounters**		
Mean(SD)	13.2 (11.0)	8.8 (9.7)
Median	10.0	5.0
Min, Max	[2.0, 53.0]	[2.0, 68.0]
**Number of anti-VEGF injections per eye**		
Mean(SD)	0.138 (0.834)	1.613 (5.459)
Median	0.000	0.000
Min, Max	[0.000, 11.000]	[0.000, 56.000]
**Treated with SDM Laser**		
No	0/737 (0.0%)	N/A
Yes	737/737 (100.0%)	N/A
**Number of Laser Treatments**		
Mean(SD)	6.3 (5.0)	N/A
n	737	N/A
Min, Median, Max	1, 5.0, 38	N/A
**Converted to wAMD**		N/A
Yes	32/737 (4.3%)	919/7370 (12.5%)
No	705/737 (95.7%)	6451/7370 (87.5%)

There were a total of 32 eyes (4.3%) in 26 subjects in the VPT group, and 919 eyes (12.5%) in 778 subjects in the SCA group that converted to wet AMD. The following Table summarizes the anti-VEGF treatments received by those eyes after conversion.

Table 5. Follow-up and treatment summary after wet AMD conversion, by study group.

**Factor**<br>Level	VPT	SCA
**N (eyes converted to wAMD)**	32	919
**wAMD Follow up Days per Eye**		
Mean(SD)	413.8 (376.1)	684.6 (497.8)
Median	298.5	604.0
Min, Max	[0.0, 1270.0]	[0.0, 2281.0]
**Number of anti-VEGF Injections per wAMD Eye**		
Mean(SD)	3.2 (2.5)	12.6 (9.9)
Median	2.0	10.0
Min, Max	[1.0, 11.0]	[1.0, 56.0]

Survival analysis by PS Stratum, VPT Non-Laser Excluded, no Encounter Matching

For conversion to wet AMD, the most appropriate method of analysis appears to be survival analysis using the initial diagnosis of dry AMD as time 0, and conversion to wet AMD as the outcome.

Note that other analysis methods were also carried out, but are not reported here (see the appendix for Poisson regression results). These alternative simpler methods produced the same general conclusions as the survival analysis.

The survival analysis was stratified by propensity score quintiles. That is, eyes were divided into five (nearly equal size) groups using the quintiles of the propensity scores.

The following plots show the cumulative wet AMD conversion by propensity score stratum.

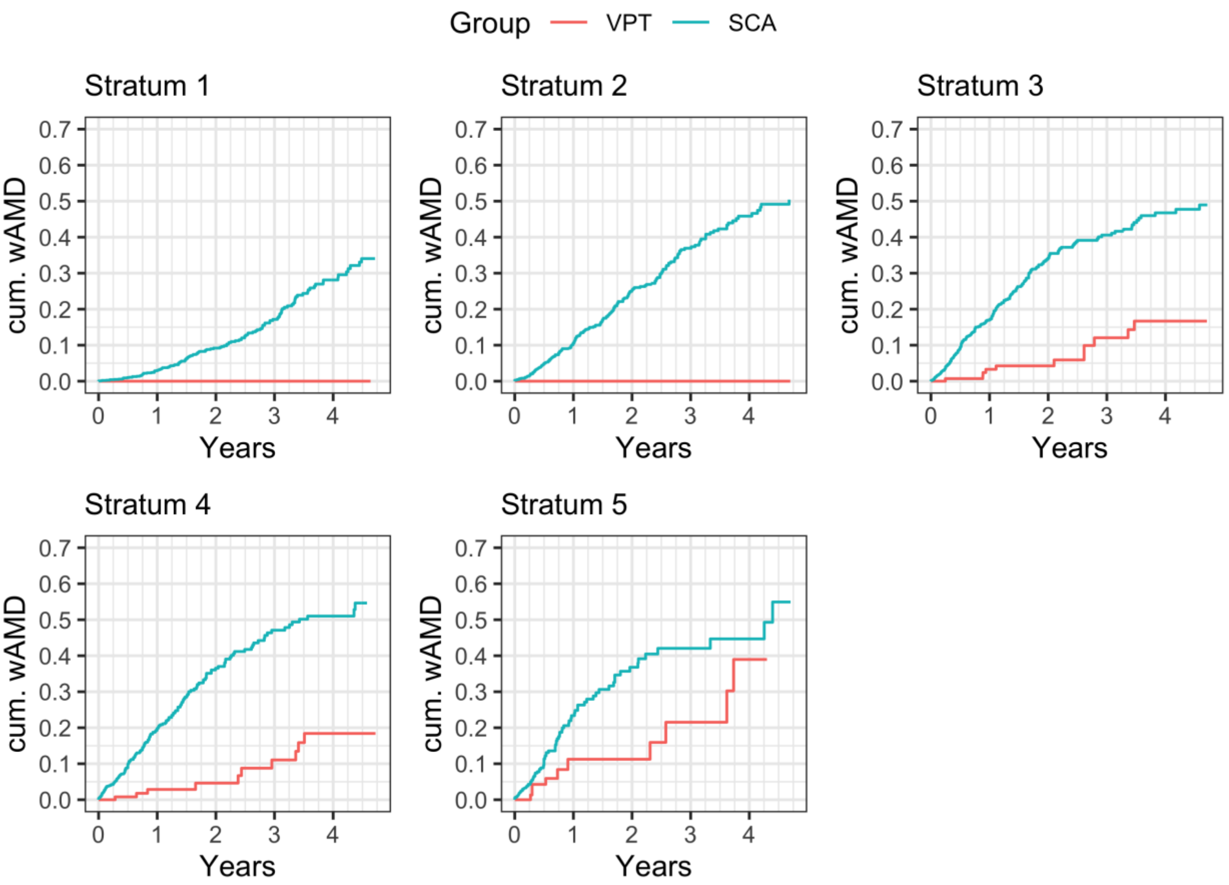

Figure 1. Cumulative probability of wAMD conversion by propensity score strata, VPT Non-Laser Excluded, no Encounter Matching.

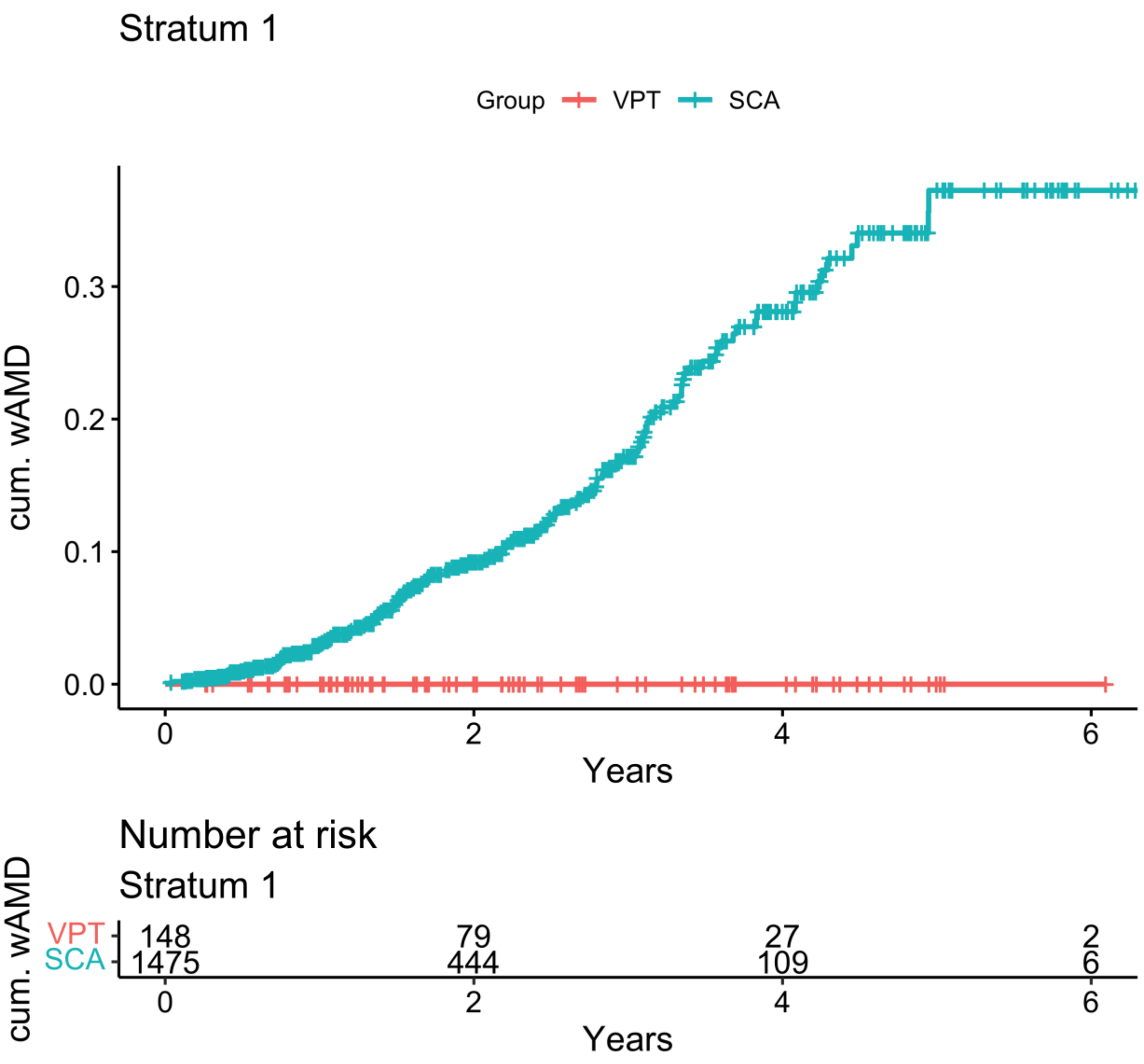
Stratum 1
Group VPT SCA
cum. wAMD
0.0
0.1
0.2
0.3
0
2
4
6
Years
Number at risk
Stratum 1
cum. wAMD
VPT 148 79 27 2
SCA 1475 444 109 6
0
2
4
6
Years

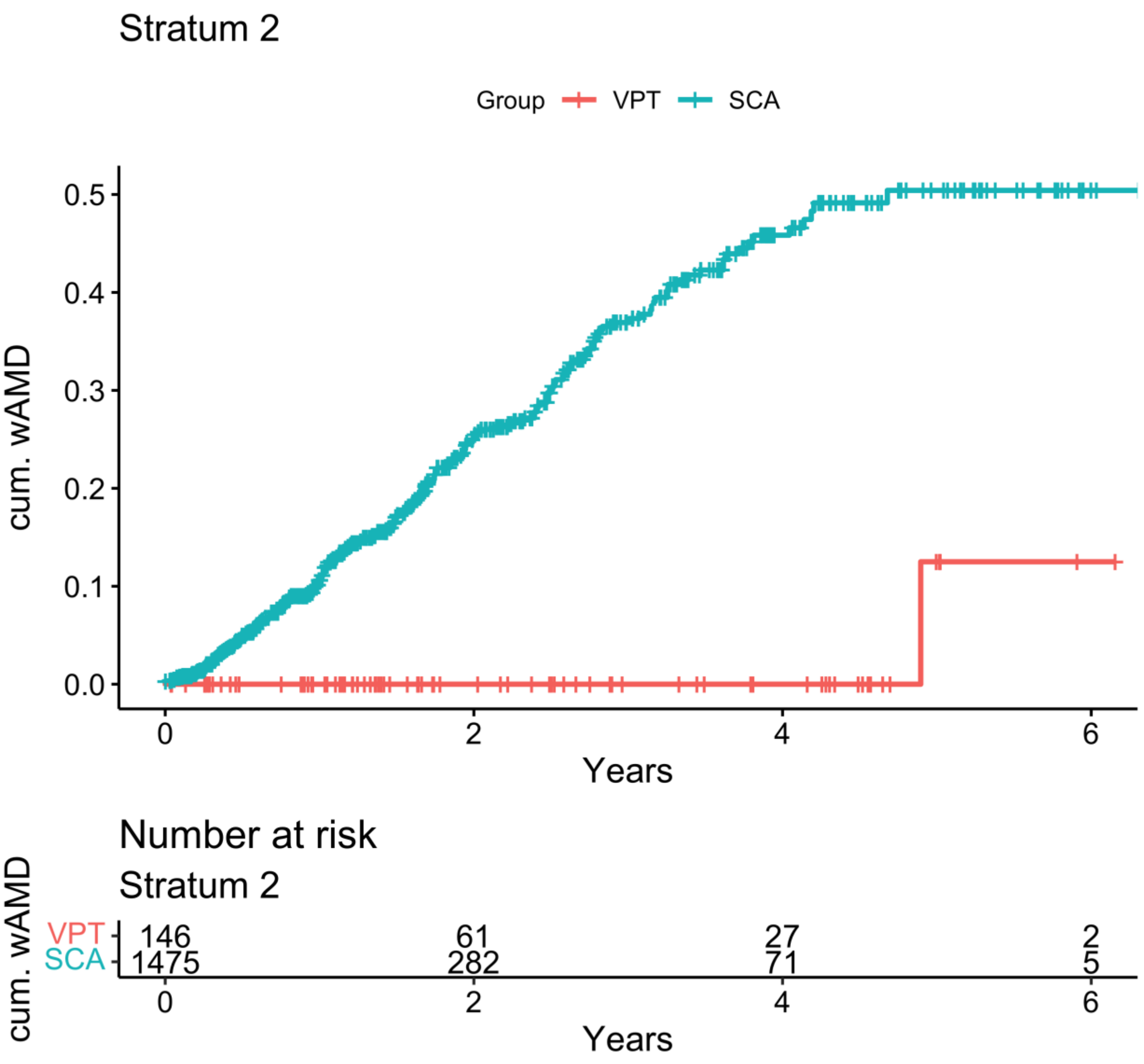

Stratum 2
Group VPT SCA
0.5
0.4
0.3
0.2
0.1
0.0
cum. wAMD
0
2
4
6
Years
Number at risk
Stratum 2
cum. wAMD
VPT 146 61 27 2
SCA 1475 282 71 5
0
2
4
6
Years

Stratum 3

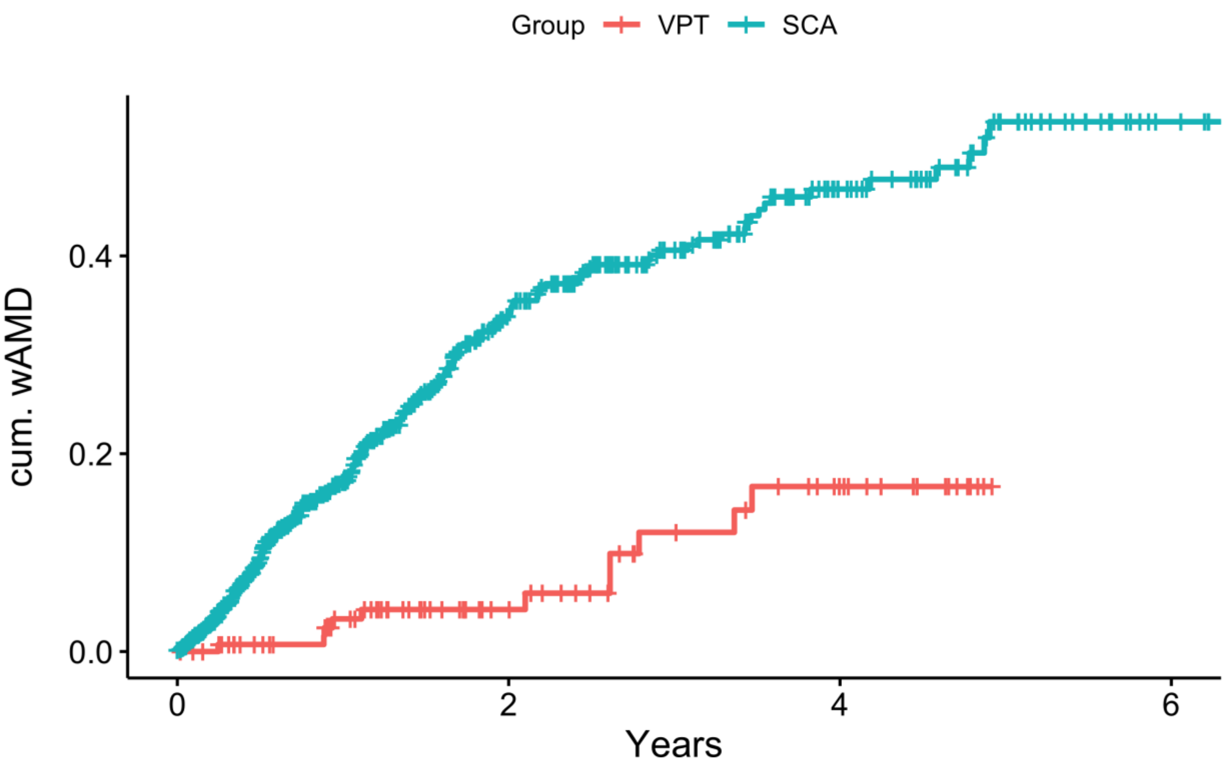

Number at risk

Stratum 3

cum. wAMD	0	2	4	6
VPT	148	60	25	0
SCA	1472	204	59	5

Years

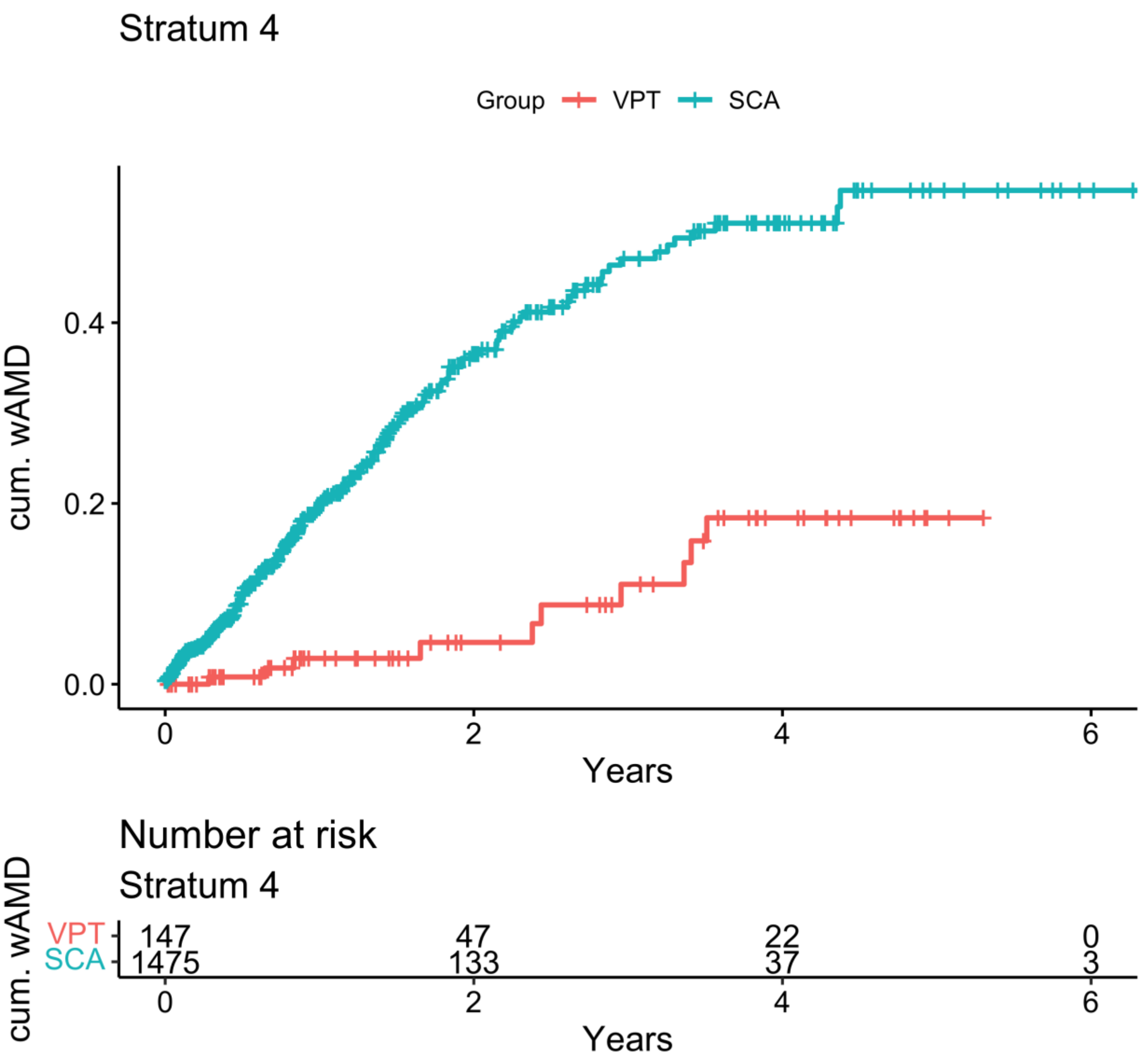
Stratum 4
Group VPT SCA
0.4
0.2
0.0
cum. wAMD
0 2 4 6
Years
Number at risk
Stratum 4
cum. wAMD
VPT 147 47 22 0
SCA 1475 133 37 3
0 2 4 6
Years

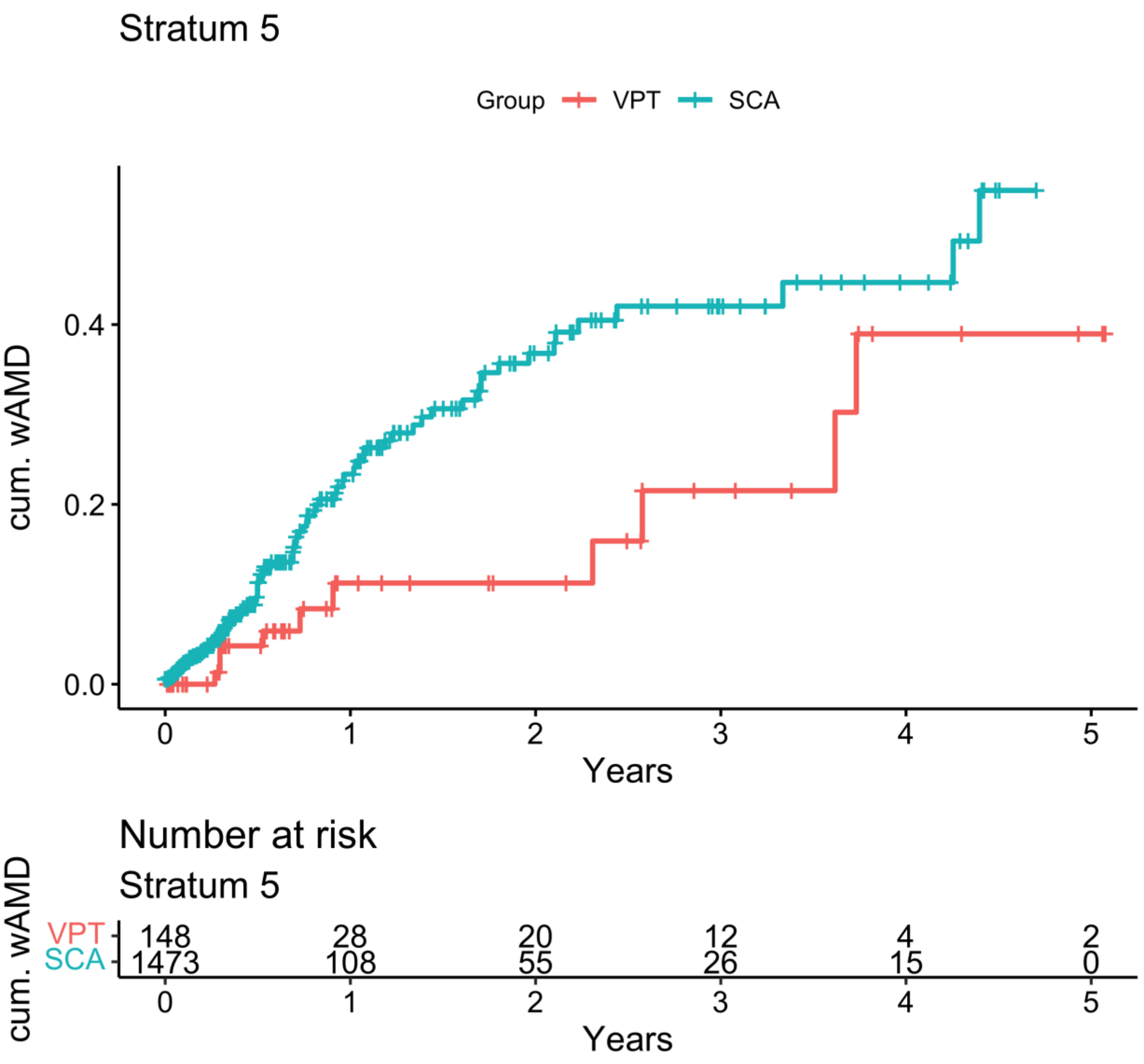

Stratum 5
Group VPT SCA
cum. wAMD
0.0
0.2
0.4
0 1 2 3 4 5
Years
Number at risk
Stratum 5
cum. wAMD
VPT 148 28 20 12 4 2
SCA 1473 108 55 26 15 0
0 1 2 3 4 5
Years

A test for equality of the Kaplan-Meier survival curves (a stratified log-rank test) shows a very significant difference in survival between the VPT and SCA groups.

Table 6. Kaplan-Meier test between Groups, stratified by propensity score quintiles. Chisq = 128.598617 on 1 degrees of freedom, p = 0.000000

	N	Observed	Expected	(O-E)^2/E	(O-E)^2/V
**Analysis.Group=VPT**	737	32	161.9	104.2	128.6
**Analysis.Group=SCA**	7370	919	789.1	21.38	128.6

To allow for inclusion of covariates, and to provide an overall summary of the results, we carried out a Cox proportional hazards regression, again stratified by propensity score quintiles.

Cox PH modelinig initially included all of the covariates included in the propensity score calculation. Non-significant variables were dropped, until the final model included the following important covariates:

- Severity
- Age
- AREDS use
- Analysis Group

Results from the Cox proportional hazards fit are shown in the following Table.

Table 7. Cox PH summary of survival difference, VPT Non-Laser Excluded, no Encounter Matching. Estimated hazard ratios are in the column labeled exp(coef).

	coef	exp(coef)	se(coef)	z	p
**SeverityUnspecified**	0.2338	1.263	0.187	1.25	0.2113
**SeverityIntermediate**	0.459	1.582	0.1278	3.592	0.000328
**SeverityNonCentralGA**	0.5841	1.793	0.164	3.561	0.0003691
**SeverityCentralGA**	0.3622	1.437	0.169	2.143	0.03213
**Age**	0.01833	1.018	0.004089	4.483	7.361e-06
**Areds.FlagYes**	0.3942	1.483	0.07869	5.009	5.473e-07
**Analysis.GroupSCA**	1.745	5.726	0.1806	9.66	0

Likelihood ratio test=250.17 on 7 df, p=0 n= 8107, number of events= 951

For severity, the "Early" group (used as the reference group in the CPH model) had the highest hazard, with all of the other severity levels producing a hazard ratio < 1. Depending on which of the 4 analysis data sets were used, several other severity levels showed significant increase in hazard over the reference group.

Subjects with AREDS use showed a significant increase in hazard (30%-50%, depending on analysis data set).

For all analysis data sets, there was a significant increase in hazard for the SCA group, of about 5.75 after adjusting for severity, age, and AREDS use (here we have (HR = 5.73, p = <1e-04)).

The following forest plot shows the parameter estimates and confidence intervals from the Cox PH regression.

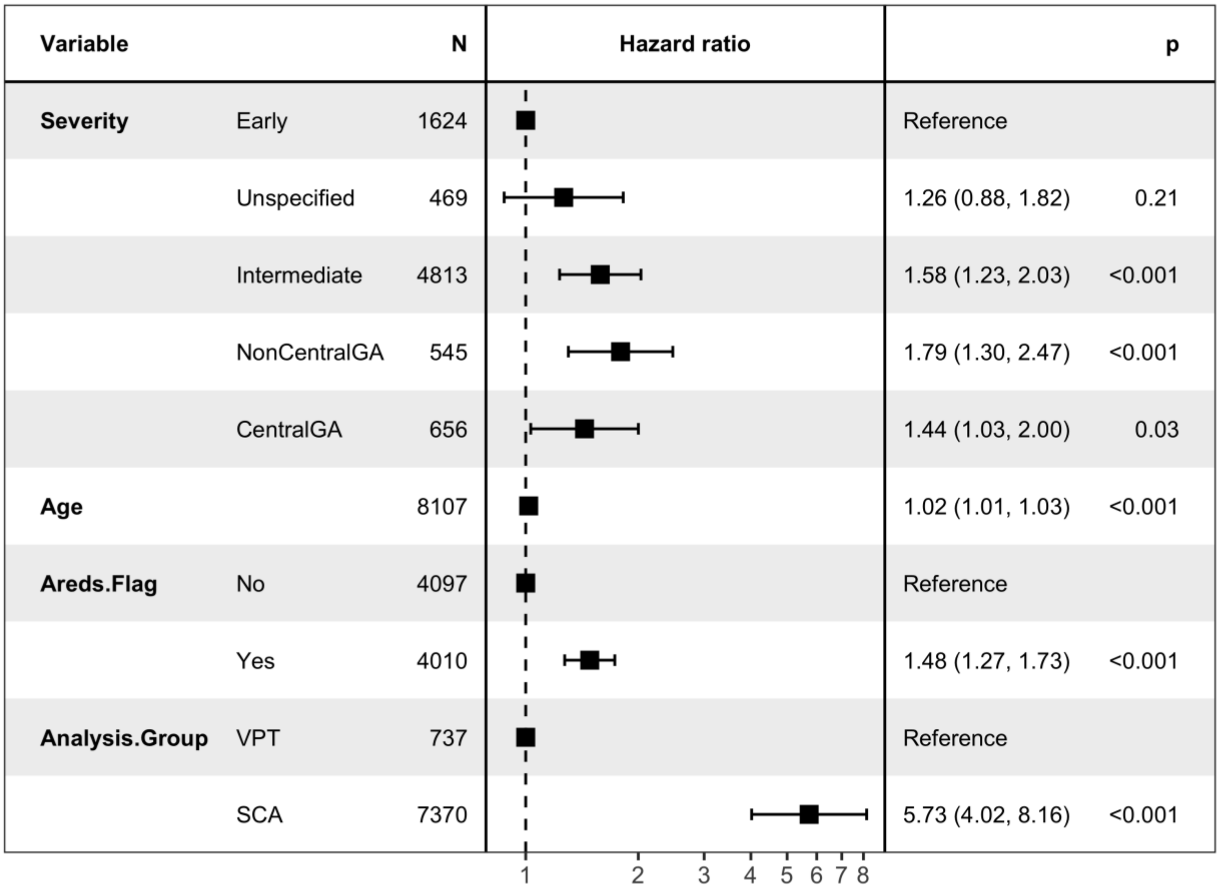

Figure 2. Forest plot of Cox PH results, VPT Non-Laser Excluded, no Encounter Matching

The test for Analysis Group shows a high level of significance.

Table 8. ANOVA for Cox PH model, VPT Non-Laser Excluded, no Encounter Matching.

	loglik	Chisq	Df	Pr(>|Chi|)
**NULL**	-5889	NA	NA	NA
**Severity**	-5873	32.91	4	1.244e-06
**Age**	-5864	18.25	1	1.937e-05
**Areds.Flag**	-5846	34.49	1	4.289e-09
**Analysis.Group**	-5764	164.5	1	1.169e-37

A test for the proportional hazards assumption shows strong evidence of non-proportionality (cox.zph(), p = 0.000461). However (see appendix), various diagnostic plots do not indicate strong non-proportionality in the Cox PH model.

Table 9. Tests for proportional hazards violations., VPT Non-Laser Excluded, no Encounter Matching.

	chisq	df	p
**Severity**	16.09	4	0.002905
**Age**	2.325	1	0.1273
**Areds.Flag**	2.244	1	0.1342
**Analysis.Group**	5.743	1	0.01655
**GLOBAL**	26.22	7	0.0004606

Summary of Survival Fits, VPT Non-Laser Excluded, no Encounter Matching

The following plot shows the overall cumulative wet AMD conversion probabilities by group, ignoring covariates.

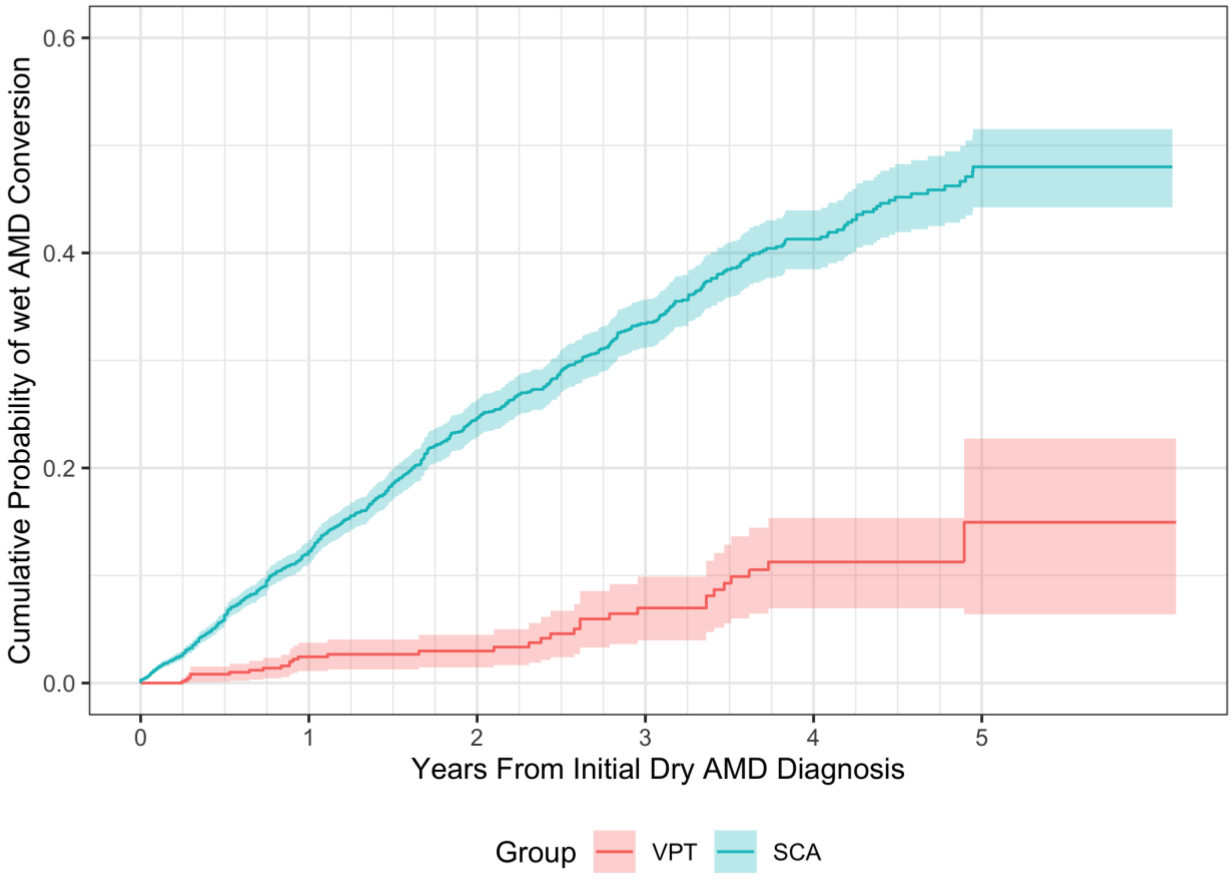

Figure 3. Overall Kaplan-Meier cumulative wet AMD conversion probability by group, VPT Non-Laser Excluded, no Encounter Matching. Shaded areas indicate 95% confidence intervals.

The following Table shows the cumulative probability of progressing to wet AMD, by year and group.

Table 10. Summary of overall survival by group (unstratified Kaplan-Meier estimates), VPT Non-Laser Excluded, no Encounter Matching.

Analysis Group	Years From DAMD Diagnosis	n at risk	n events	Cumulative Probability of wet AMD	95% CI
VPT	1	442	13	2.4%	[1.1%, 3.7%]
	2	267	2	3.0%	[1.5%, 4.5%]
	3	175	9	7.0%	[4.0%, 9.9%]
	4	105	7	11.3%	[7.0%, 15.4%]
SCA	1	2213	492	12.2%	[11.2%, 13.3%]
	2	1118	242	24.6%	[22.9%, 26.3%]
	3	608	103	33.4%	[31.2%, 35.6%]
	4	291	59	41.3%	[38.5%, 44.0%]

The hazard ratio between the two groups is summarized in the following Table. Since there are multiple eyes per person, a clustered bootstrap (clustered by subject) was used to provide a robust check on the confidence interval. The lower bound on the 95% confidence interval for the hazard ratio is above 4 using either method, again providing strong evidence for a hazard ratio greater than 1.

Table 11. Cox proportional hazards estimated hazard ratio and associated confidence intervals, VPT Non-Laser Excluded, no Encounter Matching. Cox PH model is stratified by propensity score quartiles.

Estimated Hazard Ratio	95% CI (asymptotic)	95% CI (bootstrap[1])
5.7	[4.0, 8.2]	[4.1, 9.1]

1-Bootstrap confidence interval is based on 10000 cluster (subject level) bootstrap samples.

Visual Acuity, VPT Non-Laser Excluded, no Encounter Matching

Visual acuity (ETDRS letters or the equivalent) was measured for a subset of subject visits (usually non-treatment visits). The SCA group averaged about 467.1 VA measurements per month, the VPT group averaged 50.8.

A tabulation of the mean VA by year shows a slight downward trend for the SCA group (perhaps due to aging?) but no obvious differences between the two groups.

Table 12. Mean visual acuity (ETDRS letters or equivalent) by Group and Year, VPT Non-Laser Excluded, no Encounter Matching. Cox PH model is stratified by propensity score quartiles.

Analysis Group	Mean VA 2017	Mean VA 2018	Mean VA 2019	Mean VA 2020	Mean VA 2021	Mean VA 2022
VPT	71.2	67.0	68.1	70.4	67.9	66.9
SCA	65.7	66.8	66.3	64.7	63.6	63.8

The following plot shows the mean VA per month for the SCA and VPT subjects. As expected there is more noise in the much smaller VPT group. Given the amount of noise it is difficult to assess whether there are any differences in VA through time.

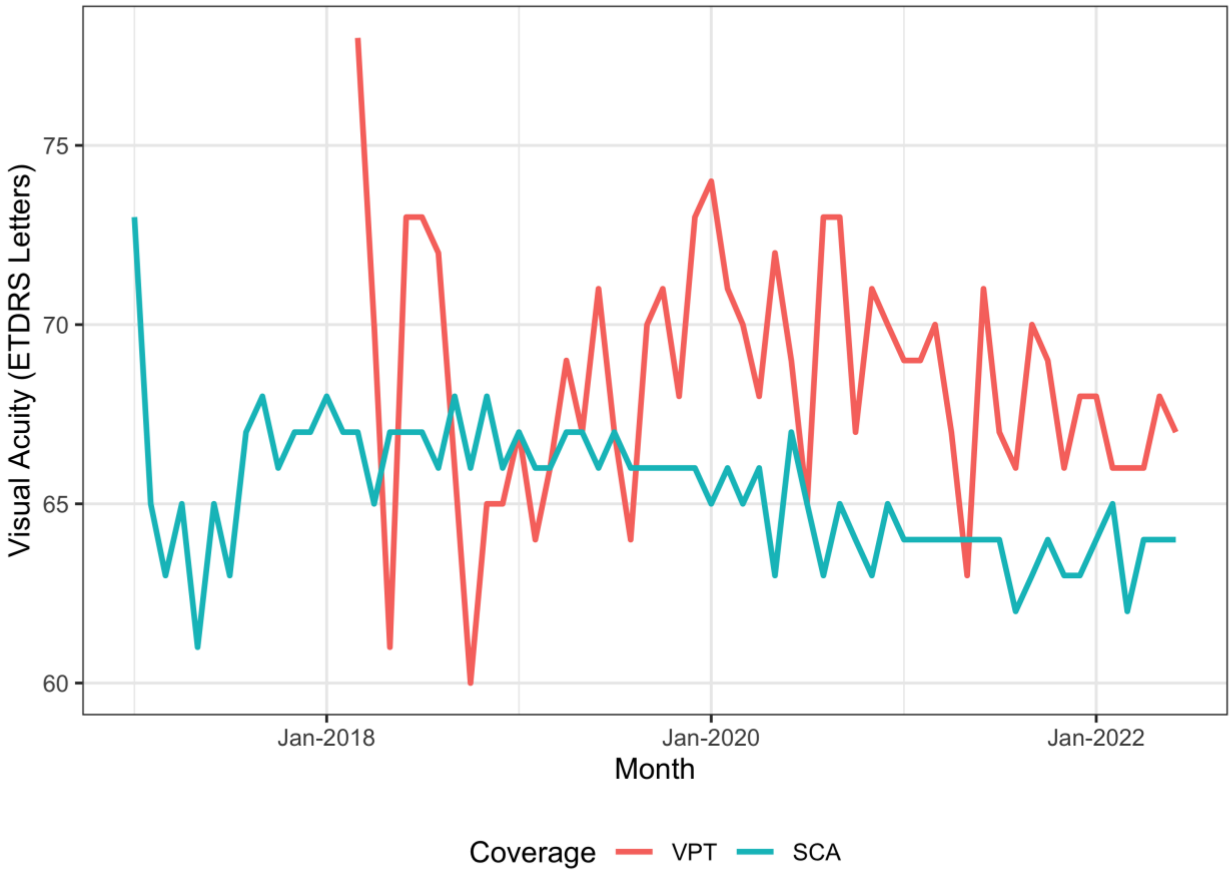

Figure 4. Visual Acuity by month, SCA, VPT Groups, VPT Non-Laser Excluded, no Encounter Matching.

A loess smoother shows a difference between the two groups during 2020-2021, perhaps due to the COVID pandemic?

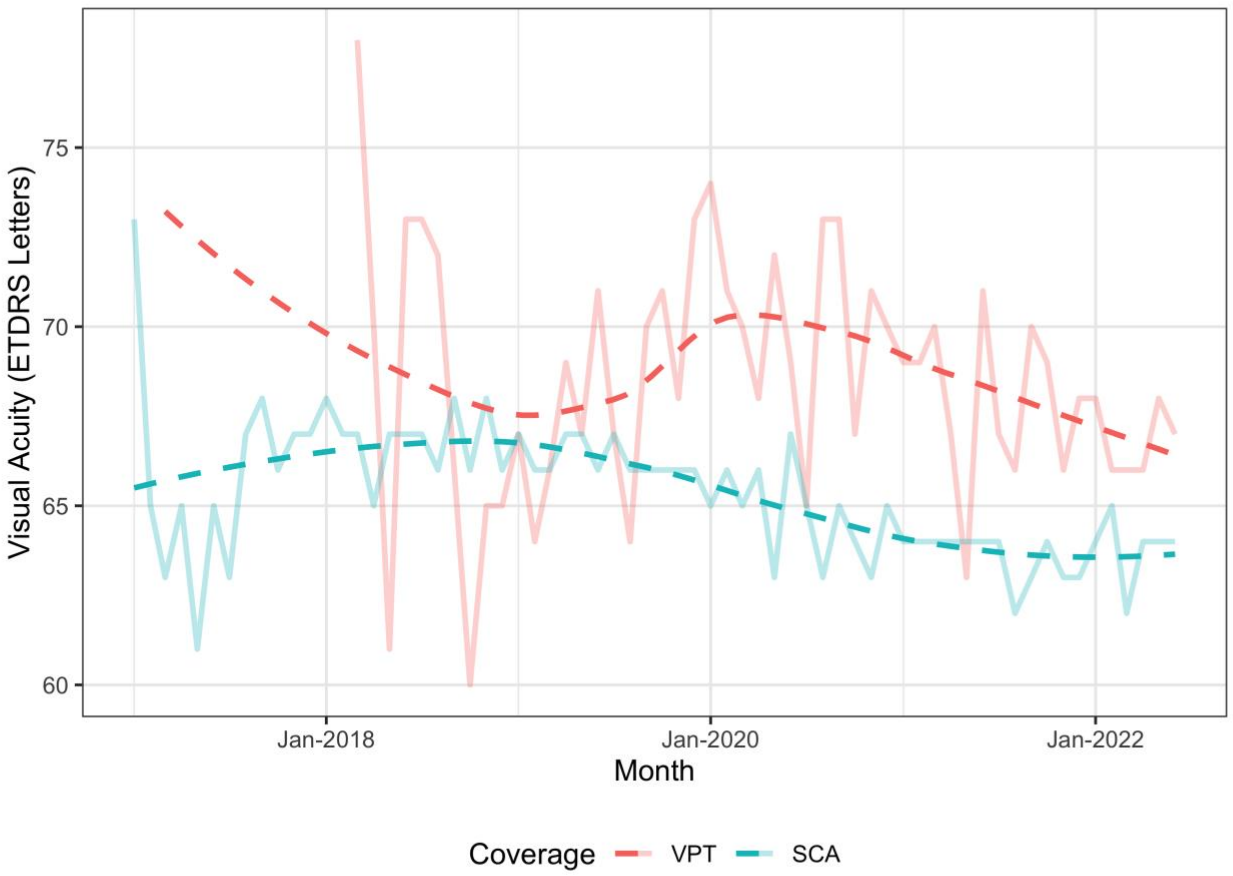

Figure 5. Visual Acuity by month, SCA, VPT Groups, with loess smooths., VPT Non-Laser Excluded, no Encounter Matching.

Caveats for Propensity Score Analysis

Potential shortcomings of this propensity score analysis include:

- The VPT group only includes subjects from a single practice, thus the generalizability of the results may be in question.
- Propensity score methods can "balance" the two groups with respect to the variables used in the propensity score model. However, they do not balance for unmeasured covariates, thus if there are other important predictors of group membership or outcome that are missing the results could be misleading.
- This analysis used subjects for whom the latest ICD-10 coding was used, which added Dry AMD severity codes not present in the earlier ICD-9. We are thus using the site-level assessment of AMD severity and assume a reasonably consistent diagnostic judgement across sites. There is no reading center or verification of the coding as would be implemented in a clinical trial.
- The ICD-10 coding used in the Vestrum database may not capture all subject outcomes, including potentially some adverse events.
- There are inconsistencies in ICD coding, for example some subjects who had a series of anti-VEGF injections were never coded as having converted to wet AMD. Thus an eye was considered converted to wet AMD if the appropriate ICD code was entered **and** at least one anti-VEGF injection was administered. The conversion date was set to earliest of the ICD coding date or the date of the first anti-VEGF injection. Alternative definitions of conversion do not change the basic conclusions.
- There are differences in follow-up time and number of encounters (reported subject-physician interactions) between the two groups, with the VPT group having more encounters and longer follow-up. As these are "post-randomization" outcomes they were not used in the initial propensity score matching. The longer follow-up in the VPT group most likely produced more events, whereas the effect of increased encounters, if any, is unknown. Thus if there is any bias due to follow-up it is likely to produce a smaller hazard ratio for SCA versus VPT.
- To match follow-up intensity between the two groups a second propensity score analysis used the mean time-between-visits for each subject as an additonal matching variable. This produces a better correspondence in follow-up time and number of visits between the two groups at the expense of potential bias issues with using post-diagnosis information in the matching.

Appendix/Supplemental

This appendix includes diagnostic plots and alternative analyses.

Distribution of Follow-Up Time

The following plot shows the follow-up time by group and wet AMD status.

Note the spikes in the SCA group at approximately 3 months, 6 months, 1 year, 2 years, and 3 years of follow-up.

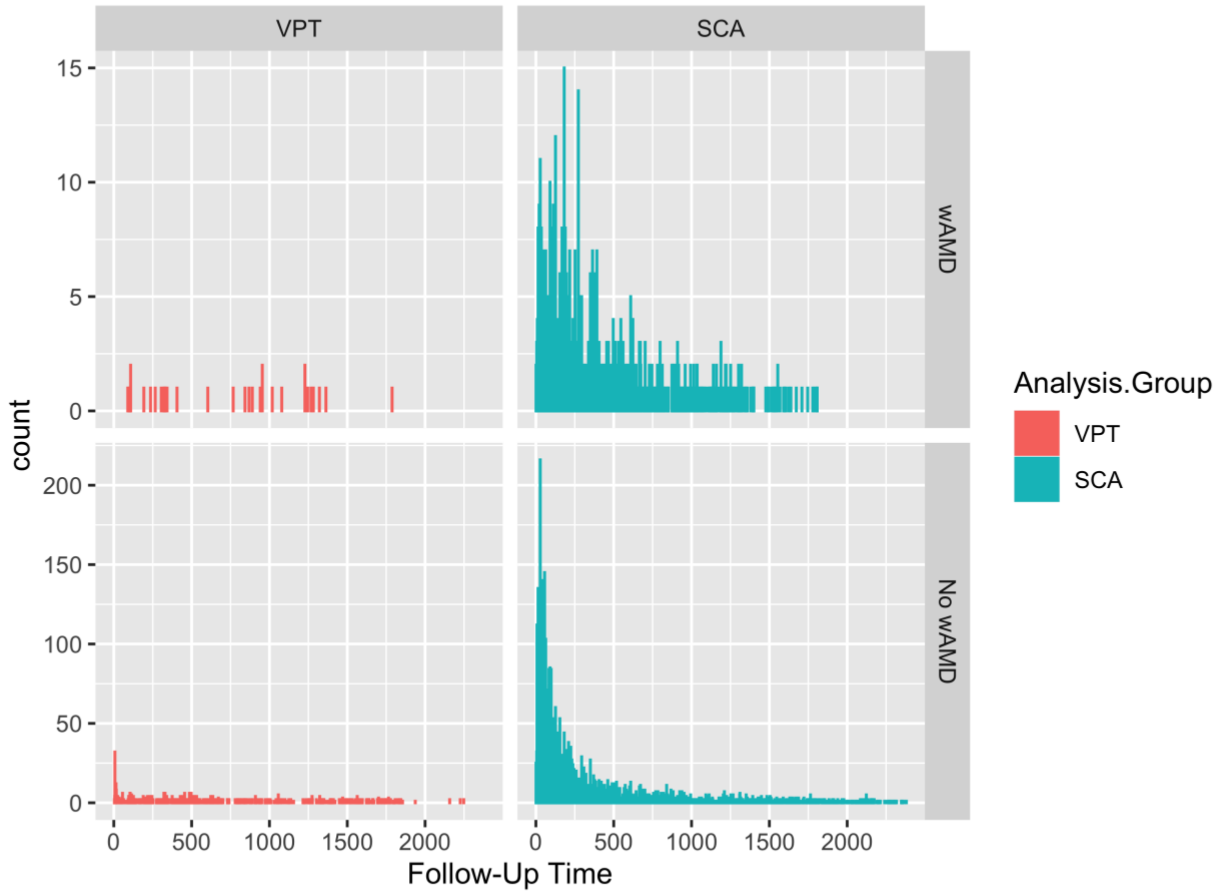

Figure 6. Follow up time by study group and wAMD conversion status. Subjects with 0 days follow-up are excluded.

Diagnostic Plots for Propensity Scores

Propensity score diagnostic plots look good overall. The overall distributions for the propensity scores are quite similar.

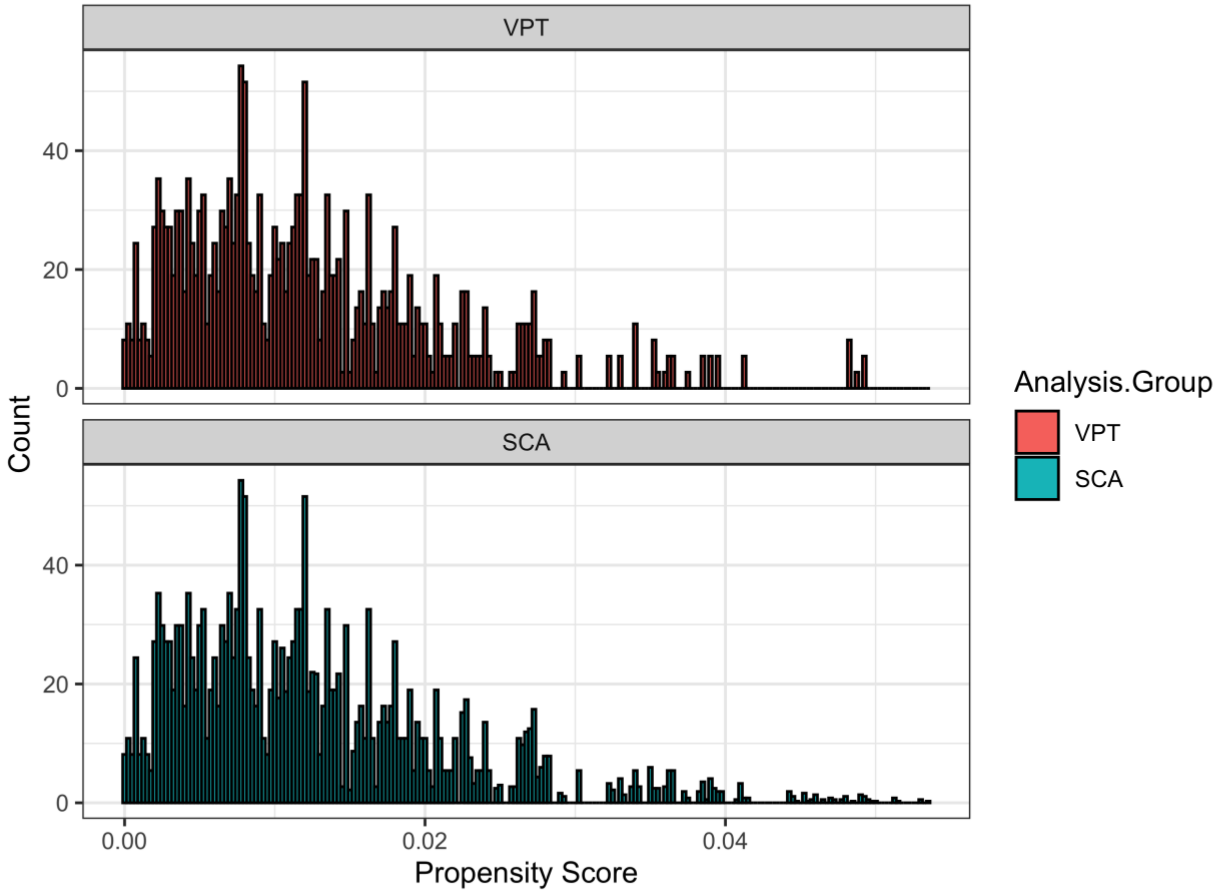

Figure 7. Diagnostic plots for propensity scores, overall distribution.

Propensity scores are also similar within each PS stratum.

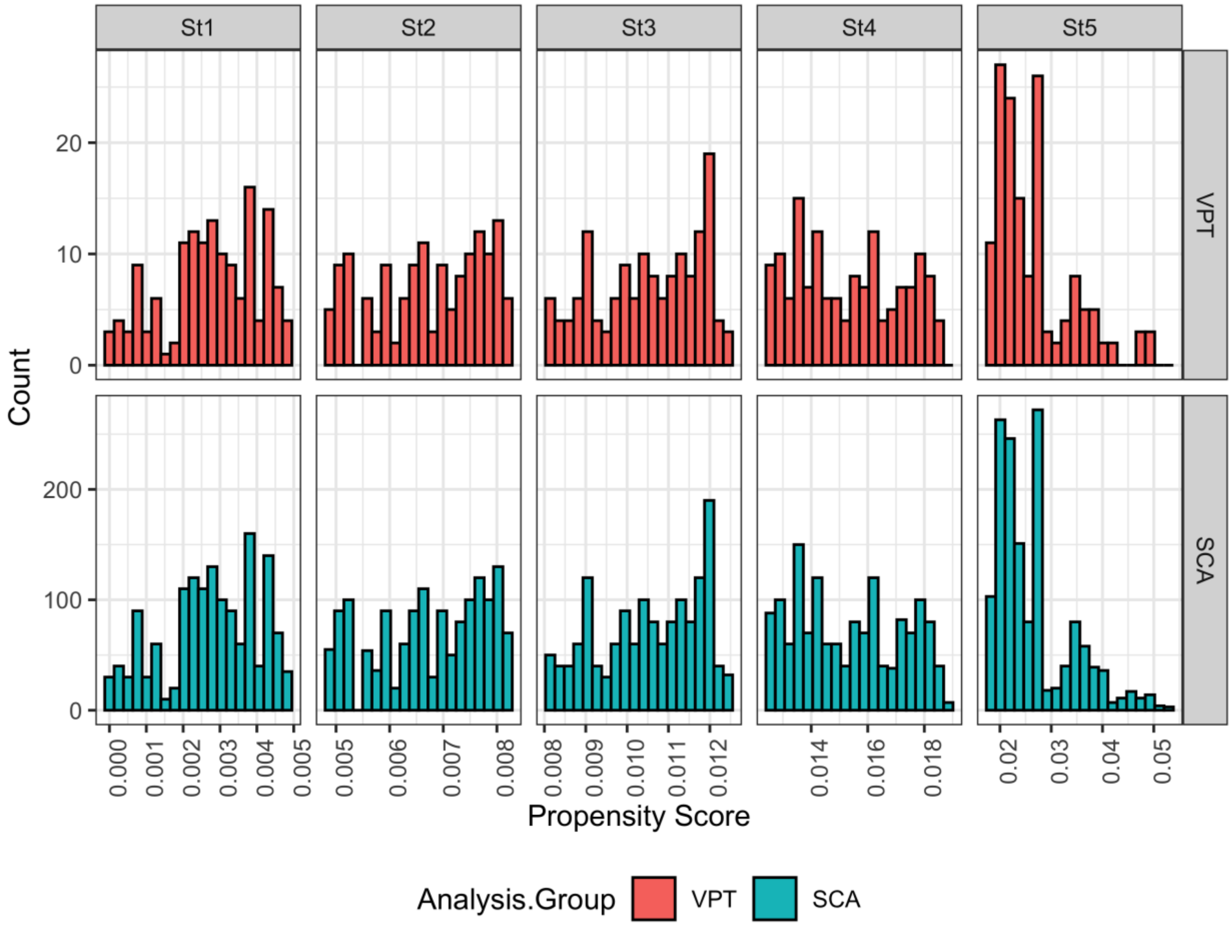

Figure 8. Diagnostic plots for propensity scores, distribution by PS stratum.

Each individual component also looks good. What we want to see is that within each stratum the two groups are similar (e.g. the pairs of orange and yellow bars have good overlap, the pairs of green bars are similar height).

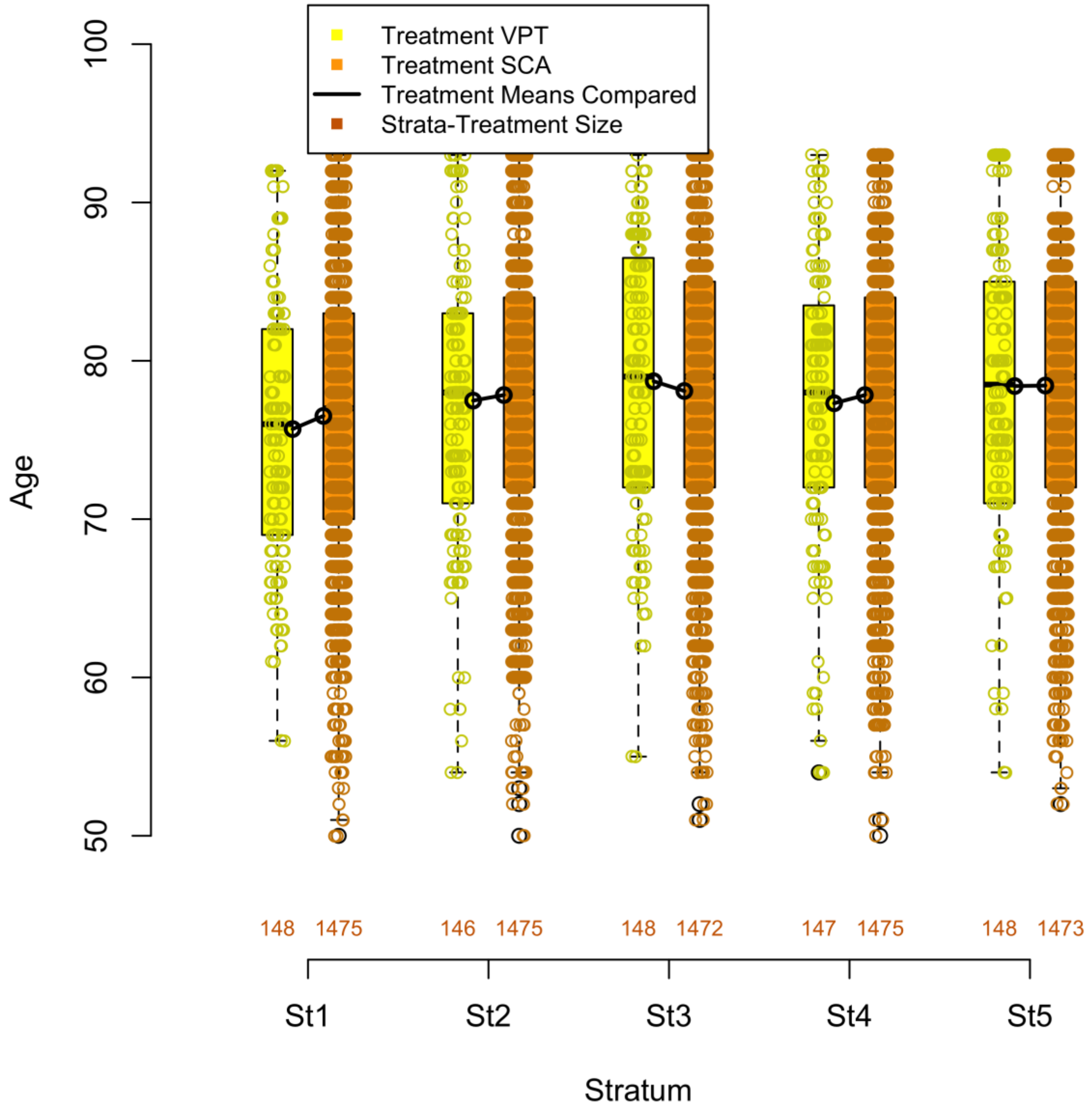

Figure 9. Diagnostic plots for propensity scores, age.

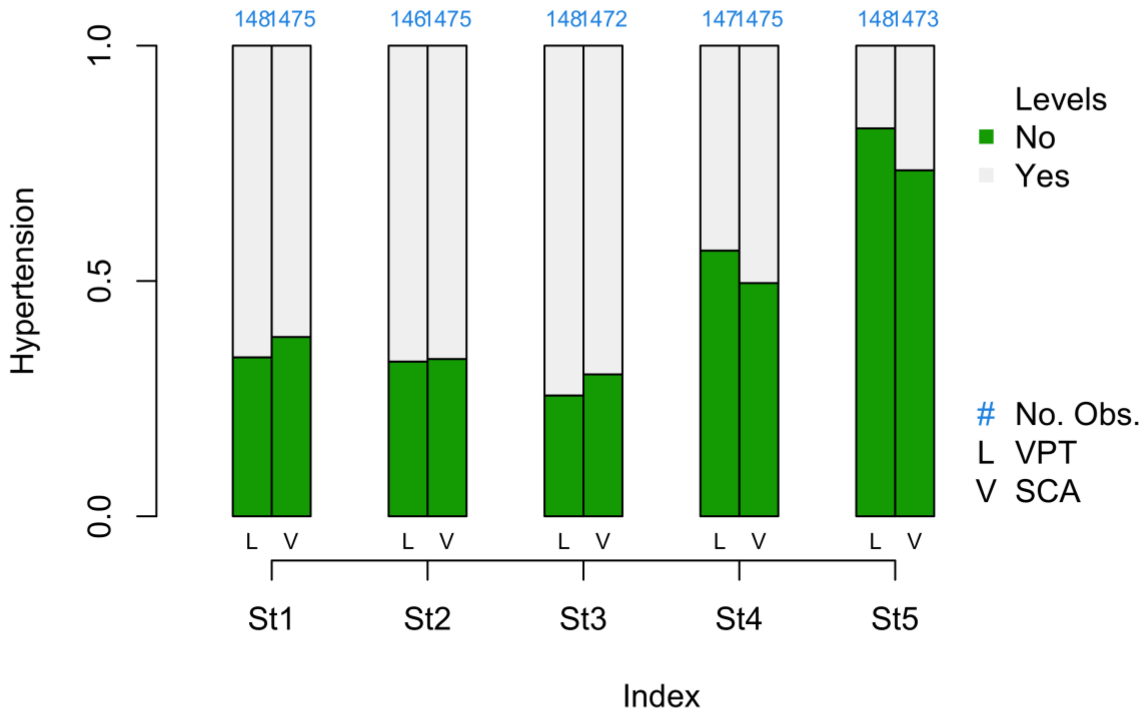

Figure 10. Diagnostic plots for propensity scores, hypertension.

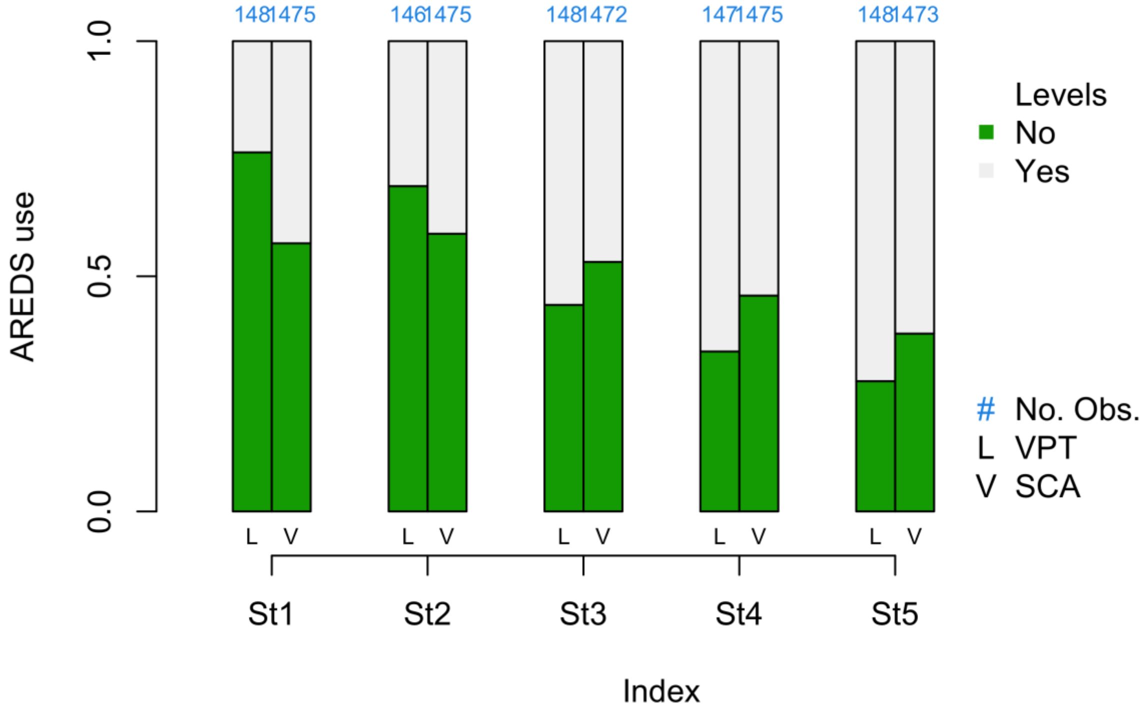

Figure 11. Diagnostic plots for propensity scores, AREDS use.

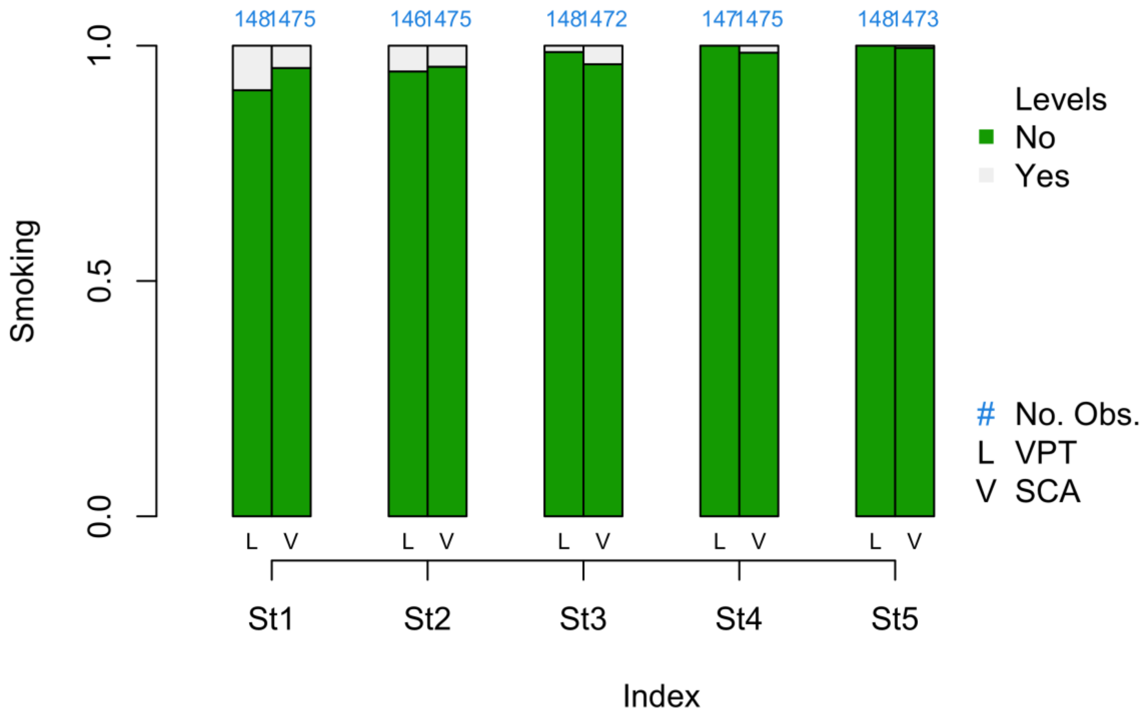

Figure 12. Diagnostic plots for propensity scores, smoking.

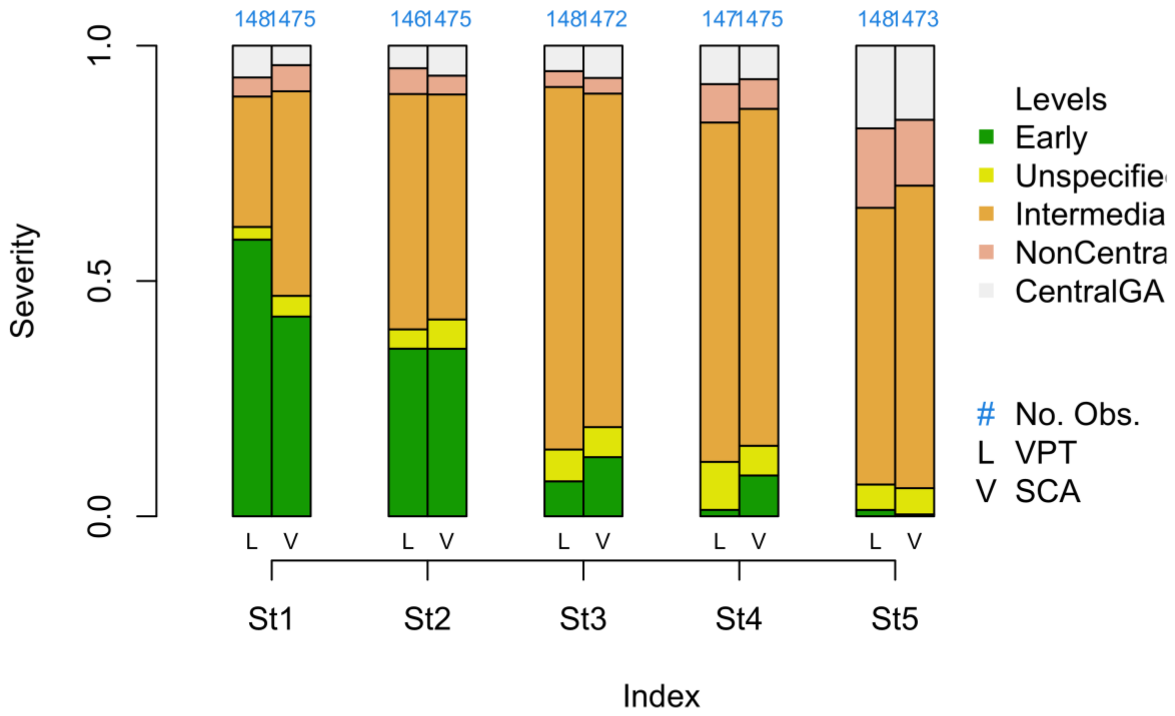

Figure 13. Diagnostic plots for propensity scores, severity

Incidence Rates via Poisson Regression

As a simpler alternative to the survival analysis, we can fit a poisson regression model. Poission regression adjusts for the follow-up on each eye individually, so corrects the bias in incidence rates somewhat. We can also easily account for the propensity score strata.

Fitting generalized (poisson/log) linear model: Converted.to.wAMD.n ~ Analysis.Group + PSStratum + offset(Follow.up.Years)

	Estimate	Std. Error	z value	Pr(>|z|)
**(Intercept)**	-7.281	0.1955	-37.25	1.031e-303
**Analysis.GroupSCA**	1.951	0.1799	10.84	2.118e-27
**PSStratumSt2**	0.7708	0.1088	7.084	1.397e-12
**PSStratumSt3**	1.137	0.106	10.72	7.817e-27
**PSStratumSt4**	1.438	0.1089	13.2	8.977e-40
**PSStratumSt5**	1.768	0.1273	13.89	7.403e-44

Table 13. Incidence rates from poisson regression (correcting for unequal follow-up in data, and adjusting for stratum differences.)

Analysis.Group	incidence rate	std.error	df	null	statistic	p.value
VPT	0.005205	0.0009223	Inf	1	-29.67	1.673e-193
SCA	0.03663	0.001269	Inf	1	-95.47	0

The incidence rate ratio from this fit is 7.0.

Diagnostic Plots for Cox PH Model

The following plots are diagnostics from the Cox proportional hazards fit.

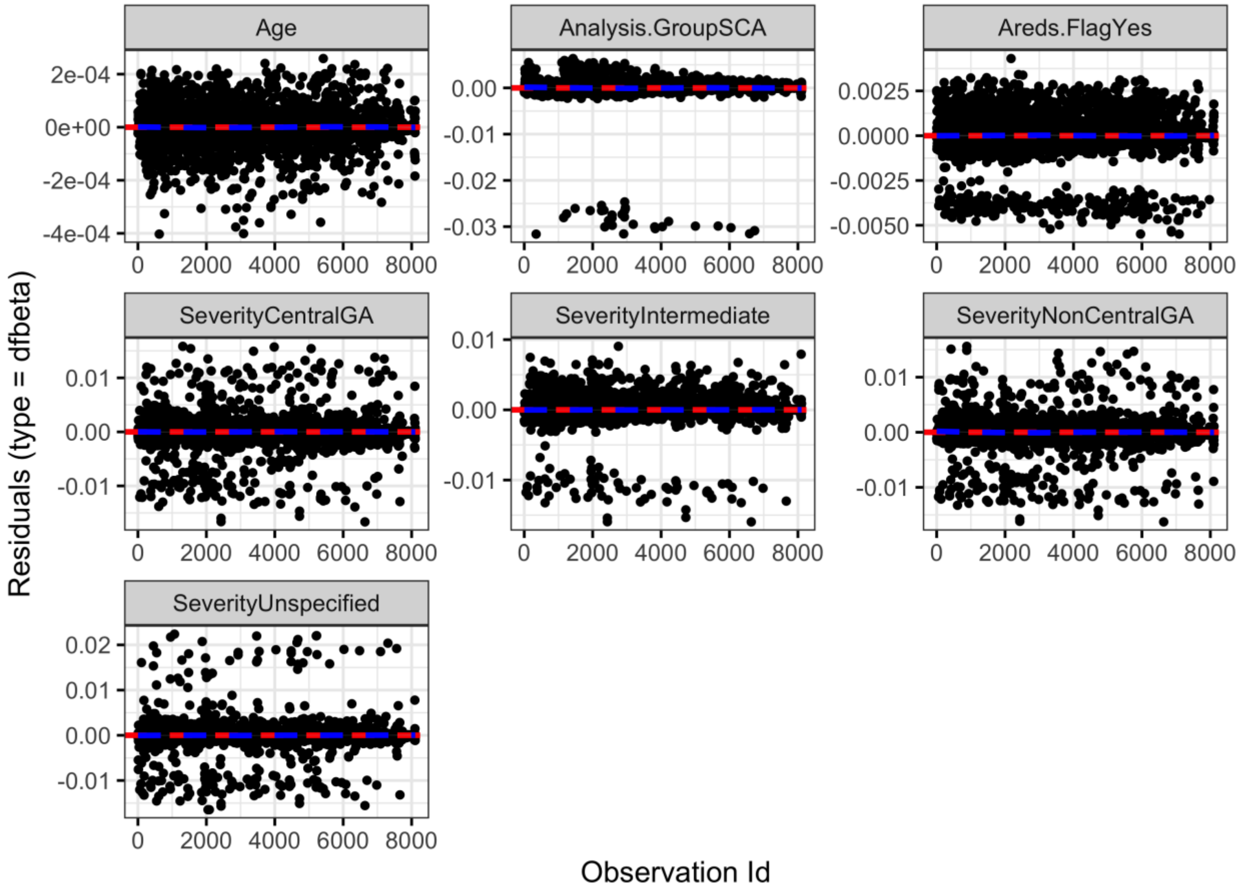

Figure 14. Dfbeta residuals from Cox PH fit.

```
## `geom_smooth()` using formula = 'y ~ x'
```

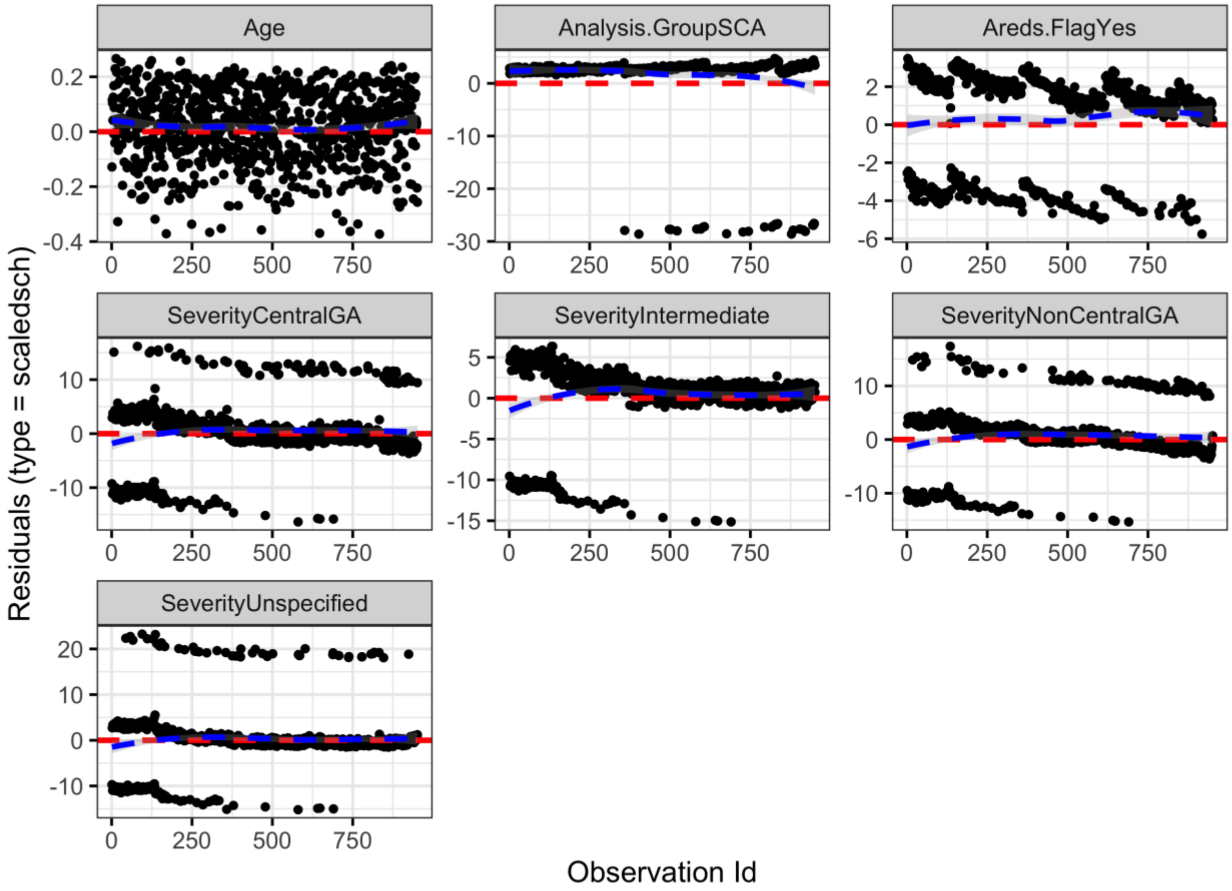

Figure 15. Scaled Schoenfeld residuals vs Time.

Retinal Protection Sciences

Comparison of SDM treatment to standard of care
Propensity matched Vestrum data,
with AMD severity coding
v6.0

[with non-laser-treated VPT subjects]
[with encounter matching]

Gerry Gray, Regulatory Pathways, Inc.

9/20/2023

Table of Contents

INTRODUCTION **66**
BASELINE TABULATIONS, VPT NON-LASER INCLUDED, WITH ENCOUNTER MATCHING **68**
TREATMENT AND RAW OUTCOMES, VPT NON-LASER INCLUDED, WITH ENCOUNTER MATCHING **69**
SURVIVAL ANALYSIS BY PS STRATUM, VPT NON-LASER INCLUDED, WITH ENCOUNTER MATCHING **71**
SUMMARY OF SURVIVAL FITS, VPT NON-LASER INCLUDED, WITH ENCOUNTER MATCHING **80**
VISUAL ACUITY, VPT NON-LASER INCLUDED, WITH ENCOUNTER MATCHING **81**
CAVEATS FOR PROPENSITY SCORE ANALYSIS **85**
APPENDIX/SUPPLEMENTAL **86**
DISTRIBUTION OF FOLLOW-UP TIME 86
DIAGNOSTIC PLOTS FOR PROPENSITY SCORES 87
INCIDENCE RATES VIA POISSON REGRESSION 94
DIAGNOSTIC PLOTS FOR COX PH MODEL 95

Introduction

This report contains analyses of propensity score matched data from the Vestrum database.

This is the second propensity score analysis from this database. One of the major perceived shortcomings of the previous analysis was that AMD severity at diagnosis was not available in the then-current ICD-9 coding. This issue was addressed in the new ICD-10 codes, which indicate AMD severity at diagnosis. This new information on initial AMD severity was used in the revised propensity score matching.

This analysis includes severity of the initial AMD diagnosis, classified as “Early”, “Unspecified”, “Intermediate”, “Non-Central GA” or “Central GA”.

There are four separate versions of this report, created to satisfy a reviewer’s request, to adhere to a principle of “no post-randomization exclusions” of subjects, and to evaluate the sensitivity of the analyses to differential follow-up intensity. The four versions are the combinations of the following:

SDM Laser Treatment

7. Including all subjects from the VPT group who met the initial filtering (I/E) criteria.
8. Including only VPT subjects who were treated with the SDM laser (a “post-randomization” event) in addition to meeting the I/E criteria.

Follow-up Intensity

9. Propensity score matching that includes follow-up intensity, using using the mean time between visits.
10. Propensity score matching that does not include follow-up intensity.

Throughout, we use the labels “VPT” to indicate Luttrull subjects, treated with standard of care and SDM laser as appropriate, and “SCA” to indicate the matched eyes from the Vestrum database.

The Vestrum database of ~500,00 eyes with visits between 1/2/2017 and 7/31/2023 were initially filtered using study inclusion/exclusion criteria to obtain a candidate set of ~200,000 eyes, including 814 VPT eyes (737 who were treated with SDM Laser). Although exclusion of VPT eyes based on treatment recieved during the study violates a statisical principle of “no post-randomization exclusions”, at the insistence of reviewers we analyzed data both with and without VPT SDM laser treated eyes.

After completion of the initial filtering, and for both 1) all VPT eyes and 2) only SDM laser-treated VPT eyes, nearest-neighbor propensity score matching was used to obtain a matched set of control eyes from the SCA group. The R Matchit package was used for the matching (R version 4.0.2, Matchit version 3.0.2).

Propensity scores were based on the following covariates:

Table 1. Variables used to perform propensity score matching

Variable
Age
Smoking status
AREDS vitamin use status
Hypertension status
AMD Severity

To obtain similar follow-up intensity between the groups, a second propensity score matching used all of the above variables plus the mean time-between-visits. These analyses are labeled “with encounter matching” in the headings.

An earlier analysis of data from the Vestrum database was reported in REF. At the time of that analysis, the Vestrum system used the then-current ICD-9 codes for AMD.

With the transition to ICD-10, the codes for AMD now include separate categories for disease severity.

Table 2. ICD 10 AMD Severity codings.

AMD Severity	ICD Code	Description
Early	H35.31X1	early dry AMD—a combination of multiple small drusen (≤ 63 μm), few intermediate drusen (> 63 μm and ≤ 124 μm), or retinal pigment epithelium (RPE) abnormalities
Intermediate	H35.31X2	intermediate dry AMD—extensive intermediate drusen (> 63 μm and ≤ 124 μm) or at least 1 large drusen (≥ 125 μm
NonCentral GA	H35.31X3	advanced atrophic dry AMD without subfoveal involvement—geographic atrophy (GA) not involving the center of the fovea
Central GA	H35.31X4	advanced atrophic dry AMD with subfoveal involvement—GA involving the center of the fovea
Unspecified	H35.31X0, H35.31,H35.312,H35.311,H35.313	

Diagnostics from the matching indicated a good overlap of propensity scores between the two groups (see appendix).

Eyes were considered to have “converted” to wet AMD during the follow-up period if both of the following occurred:

- an ICD code for wet AMD was entered into the database
- anti-VEGF injections were initiated

The time of wet AMD conversion was the earliest of the date where the ICD code was entered or the date of the first anti-VEGF injection.

Baseline Tabulations, VPT Non-Laser Included, with Encounter Matching

The following Table summarizes the demographics of the two Groups in this analysis.

Table 3. Demographics by study group, after propensity score matching.

**Factor**<br>Level	VPT	SCA
**N (study eyes)**	814	8140
**N (subjects)**	441	5114
**Gender**		
Female	275/441 (62.4%)	2989/5114 (58.4%)
Male	166/441 (37.6%)	2053/5114 (40.1%)
Other	0/441 (0.0%)	72/5114 (1.4%)
**Age (years)**		
Mean(SD)	77.2 (9.2)	77.3 (9.2)
Median	77.0	78.0
Min, Max	[52.0, 93.0]	[50.0, 93.0]
**Age (category)**		
Age: [50,65]	41/441 (9.3%)	535/5114 (10.5%)
Age: (65,70]	61/441 (13.8%)	671/5114 (13.1%)
Age: (70,75]	88/441 (20.0%)	939/5114 (18.4%)
Age: (75,80]	81/441 (18.4%)	1015/5114 (19.8%)
Age: (80,85]	76/441 (17.2%)	868/5114 (17.0%)
Age: (85,90]	55/441 (12.5%)	608/5114 (11.9%)
Age: (90,110]	39/441 (8.8%)	478/5114 (9.3%)
**Hypertension**		
No	207/441 (46.9%)	2574/5114 (50.3%)
Yes	234/441 (53.1%)	2540/5114 (49.7%)
**AREDS use**		
No	235/441 (53.3%)	2795/5114 (54.7%)
Yes	206/441 (46.7%)	2319/5114 (45.3%)
**Smoking**		
No	427/441 (96.8%)	4996/5114 (97.7%)
Yes	14/441 (3.2%)	118/5114 (2.3%)
**AMD Severity**		
Early	123/441 (27.9%)	1305/5114 (25.5%)
Unspecified	23/441 (5.2%)	282/5114 (5.5%)
Intermediate	239/441 (54.2%)	2710/5114 (53.0%)
NonCentralGA	41/441 (9.3%)	394/5114 (7.7%)
CentralGA	39/441 (8.8%)	467/5114 (9.1%)

Treatment and Raw Outcomes, VPT Non-Laser Included, with Encounter Matching

The following Table summarizes followup and AMD treatments received.

Table 4. Follow-up and treatment summary by study group, after propensity score matching.

**Factor**<br>Level	VPT	SCA
**N (study eyes)**	814	8140
**Total Follow-up Days**		
Mean(SD)	658.2 (571.3)	609.6 (642.7)
Median	486.0	341.0
Min, Max	[4.0, 2248.0]	[0.0, 2392.0]
**Follow Up Years (categories)**		
0 ≤ Follow Up Yrs ≤ 1	331/814 (40.7%)	4792/8140 (58.9%)
1 < Follow Up Yrs ≤ 2	194/814 (23.8%)	1359/8140 (16.7%)
Follow Up Yrs > 2	274/814 (33.7%)	1710/8140 (21.0%)
**Number of Encounters**		
Mean(SD)	12.4 (10.8)	11.6 (11.7)
Median	9.0	7.0
Min, Max	[2.0, 53.0]	[2.0, 70.0]
**Number of anti-VEGF injections per eye**		
Mean(SD)	0.125 (0.795)	2.388 (6.748)
Median	0.000	0.000
Min, Max	[0.000, 11.000]	[0.000, 58.000]
**Treated with SDM Laser**		
No	77/814 (9.5%)	N/A
Yes	737/814 (90.5%)	N/A
**Number of Laser Treatments**		
Mean(SD)	6.3 (5.0)	N/A
n	737	N/A
Min, Median, Max	1, 5.0, 38	N/A
**Converted to wAMD**		N/A
Yes	32/814 (3.9%)	1321/8140 (16.2%)
No	782/814 (96.1%)	6819/8140 (83.8%)

There were a total of 32 eyes (3.9%) in 26 subjects in the VPT group, and 1321 eyes (16.2%) in 1082 subjects in the SCA group that converted to wet AMD. The following Table summarizes the anti-VEGF treatments received by those eyes after conversion.

Table 5. Follow-up and treatment summary after wet AMD conversion, by study group.

**Factor**<br>Level	VPT	SCA
**N (eyes converted to wAMD)**	32	1321
**wAMD Follow up Days per Eye**		
Mean(SD)	413.8 (376.1)	816.1 (530.4)
Median	298.5	763.0
Min, Max	[0.0, 1270.0]	[0.0, 2327.0]
**Number of anti-VEGF Injections per wAMD Eye**		
Mean(SD)	3.2 (2.5)	14.5 (10.2)
Median	2.0	13.0
Min, Max	[1.0, 11.0]	[1.0, 58.0]

Survival analysis by PS Stratum, VPT Non-Laser Included, with Encounter Matching

For conversion to wet AMD, the most appropriate method of analysis appears to be survival analysis using the initial diagnosis of dry AMD as time 0, and conversion to wet AMD as the outcome.

Note that other analysis methods were also carried out, but are not reported here (see the appendix for Poisson regression results). These alternative simpler methods produced the same general conclusions as the survival analysis.

The survival analysis was stratified by propensity score quintiles. That is, eyes were divided into five (nearly equal size) groups using the quintiles of the propensity scores.

The following plots show the cumulative wet AMD conversion by propensity score stratum.

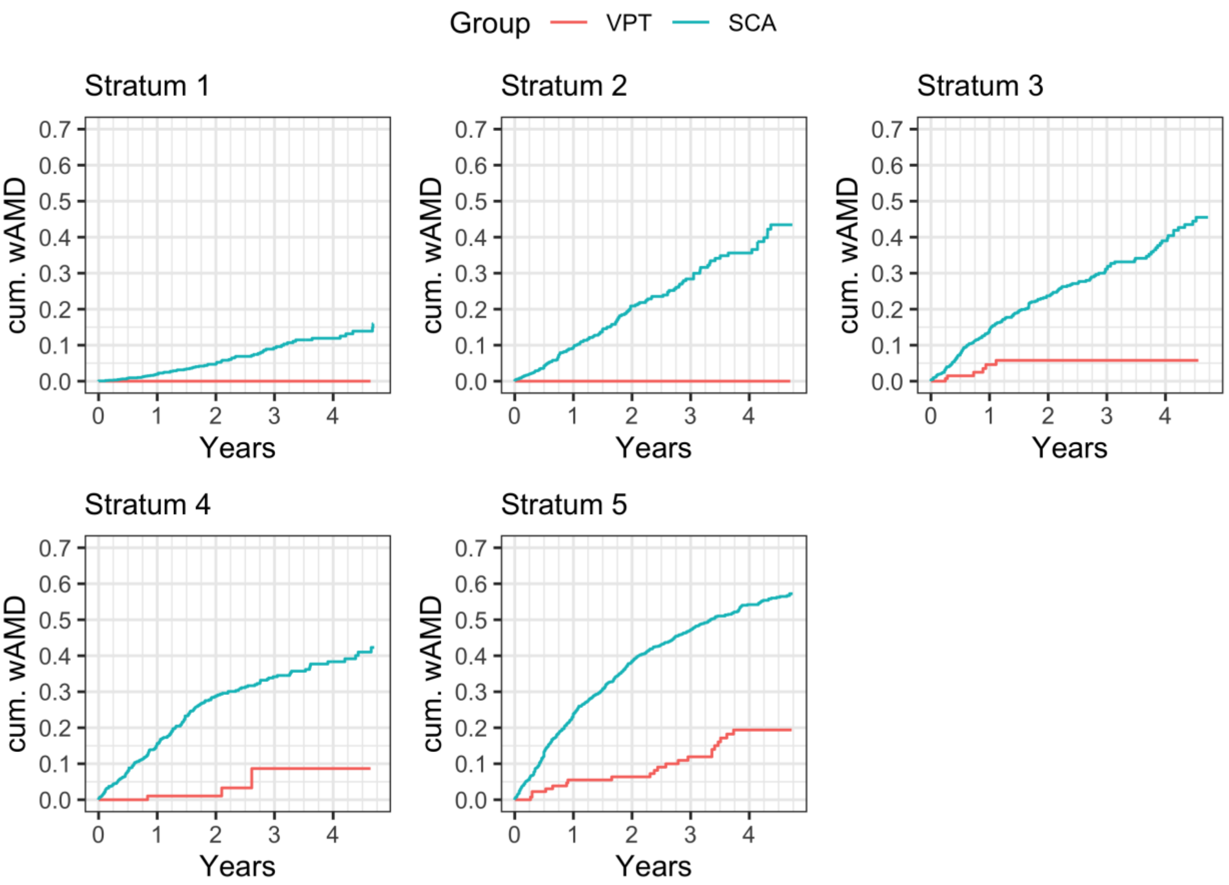

Figure 1. Cumulative probability of wAMD conversion by propensity score strata.

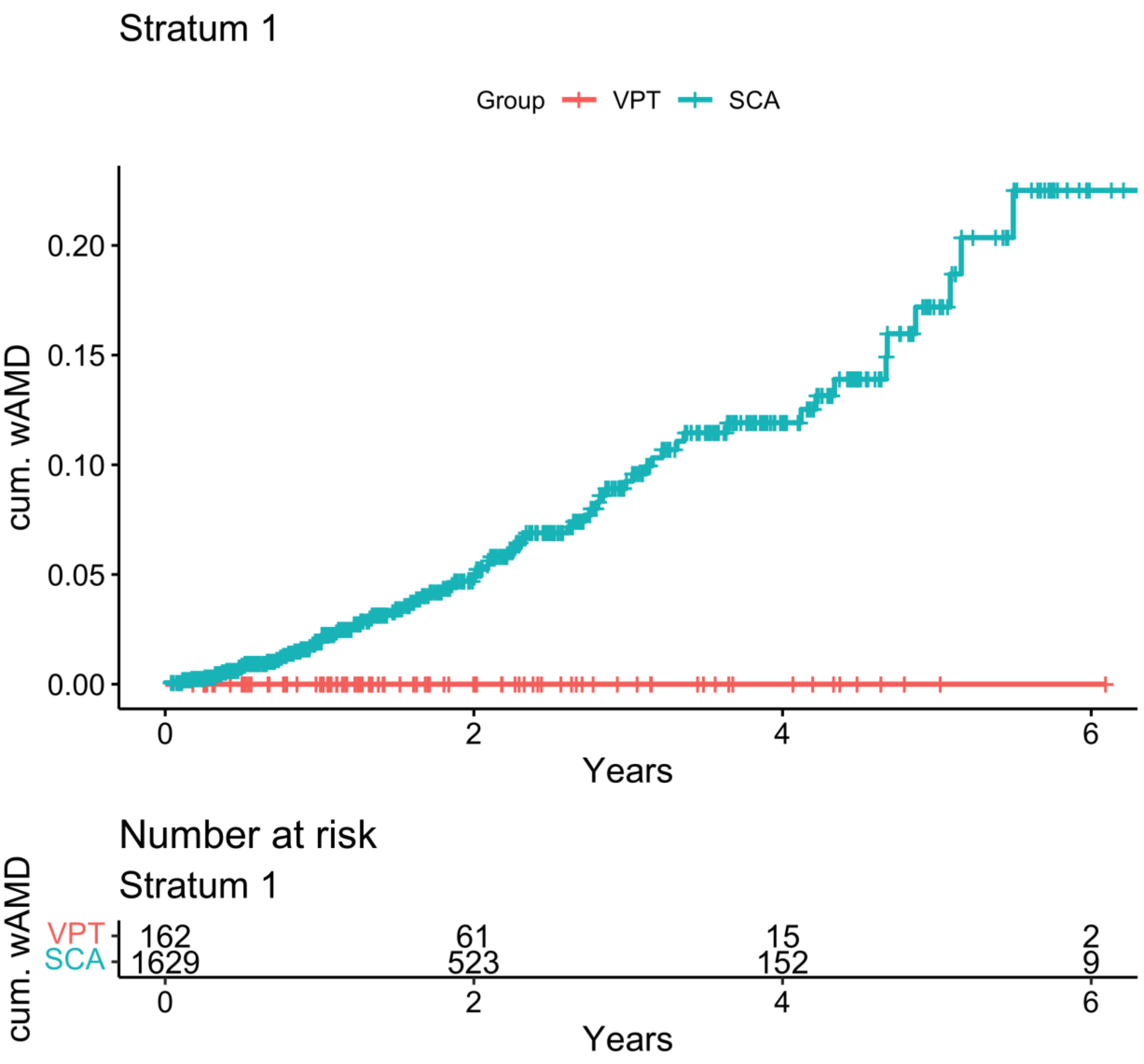

Stratum 1
Group VPT SCA
cum. wAMD
0.20
0.15
0.10
0.05
0.00
0
2
4
6
Years
Number at risk
Stratum 1
cum. wAMD
VPT 162 61 15 2
SCA 1629 523 152 9
0
2
4
6
Years

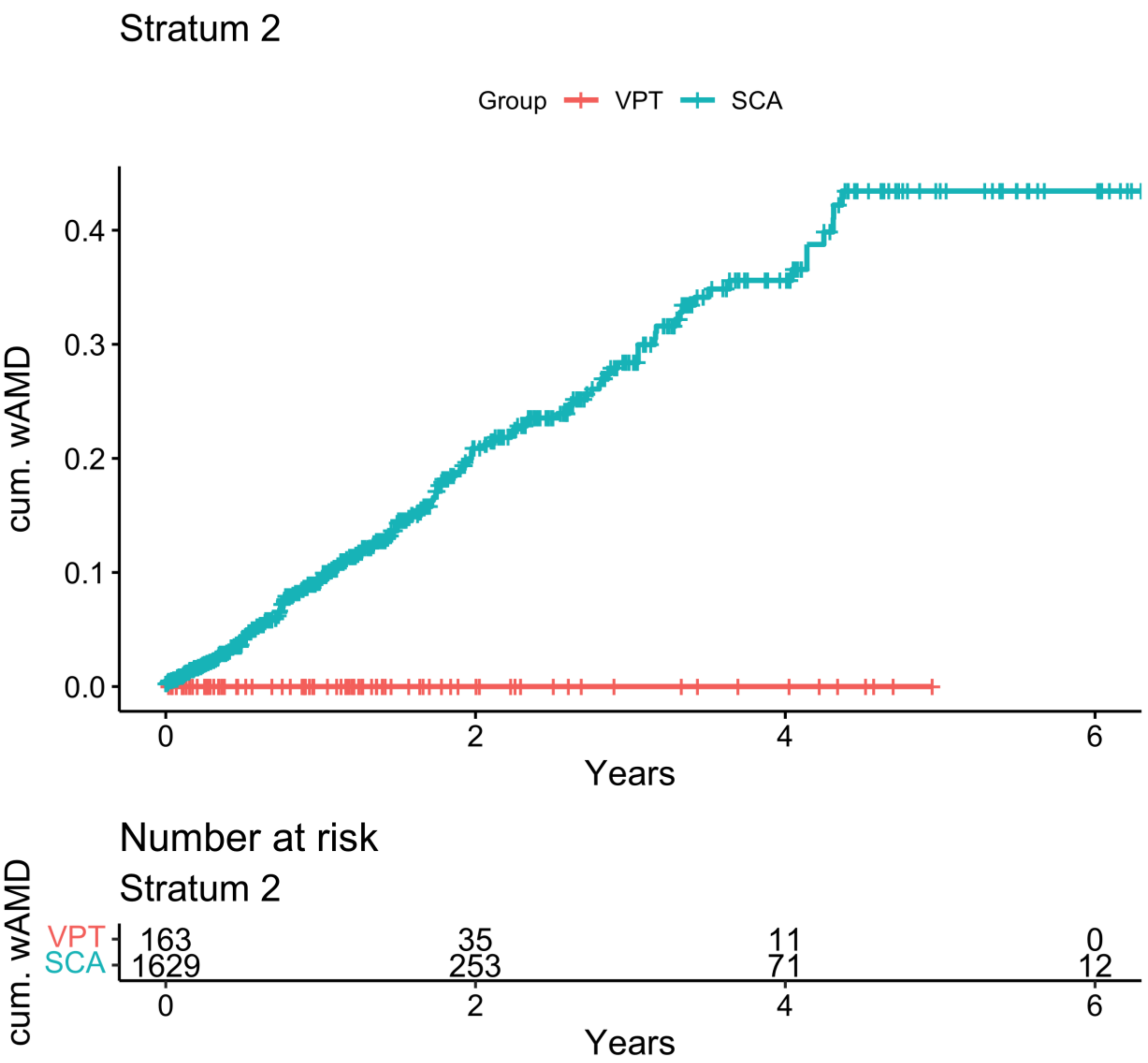

Stratum 2
Group VPT SCA
cum. wAMD
0.0
0.1
0.2
0.3
0.4
0
2
4
6
Years
Number at risk
Stratum 2
cum. wAMD
VPT 163 35 11 0
SCA 1629 253 71 12
0
2
4
6
Years

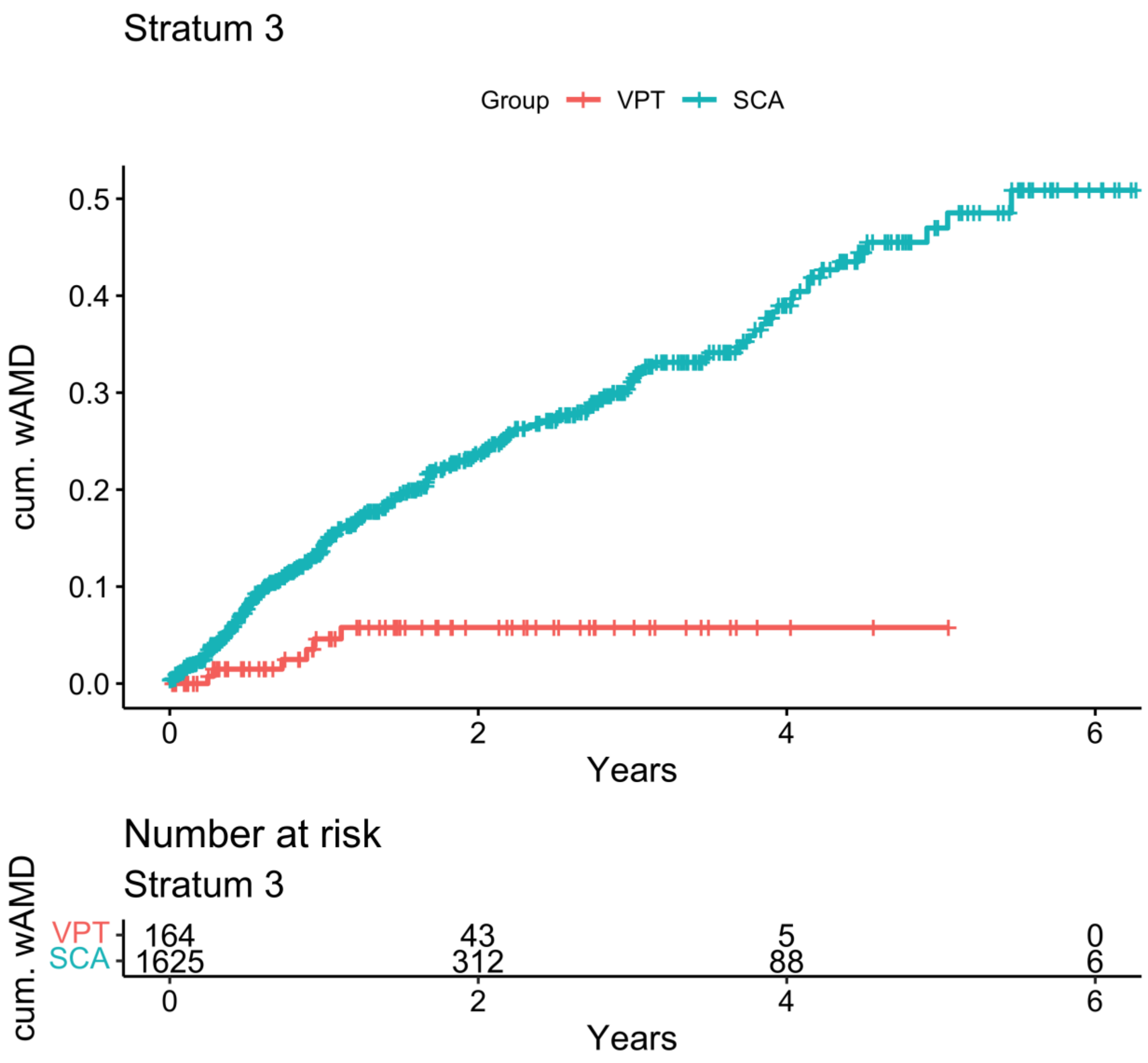
Stratum 3
Group VPT SCA
cum. wAMD
0.0
0.1
0.2
0.3
0.4
0.5
0
2
4
6
Years
Number at risk
Stratum 3
cum. wAMD
VPT 164 43 5 0
SCA 1625 312 88 6
0
2
4
6
Years

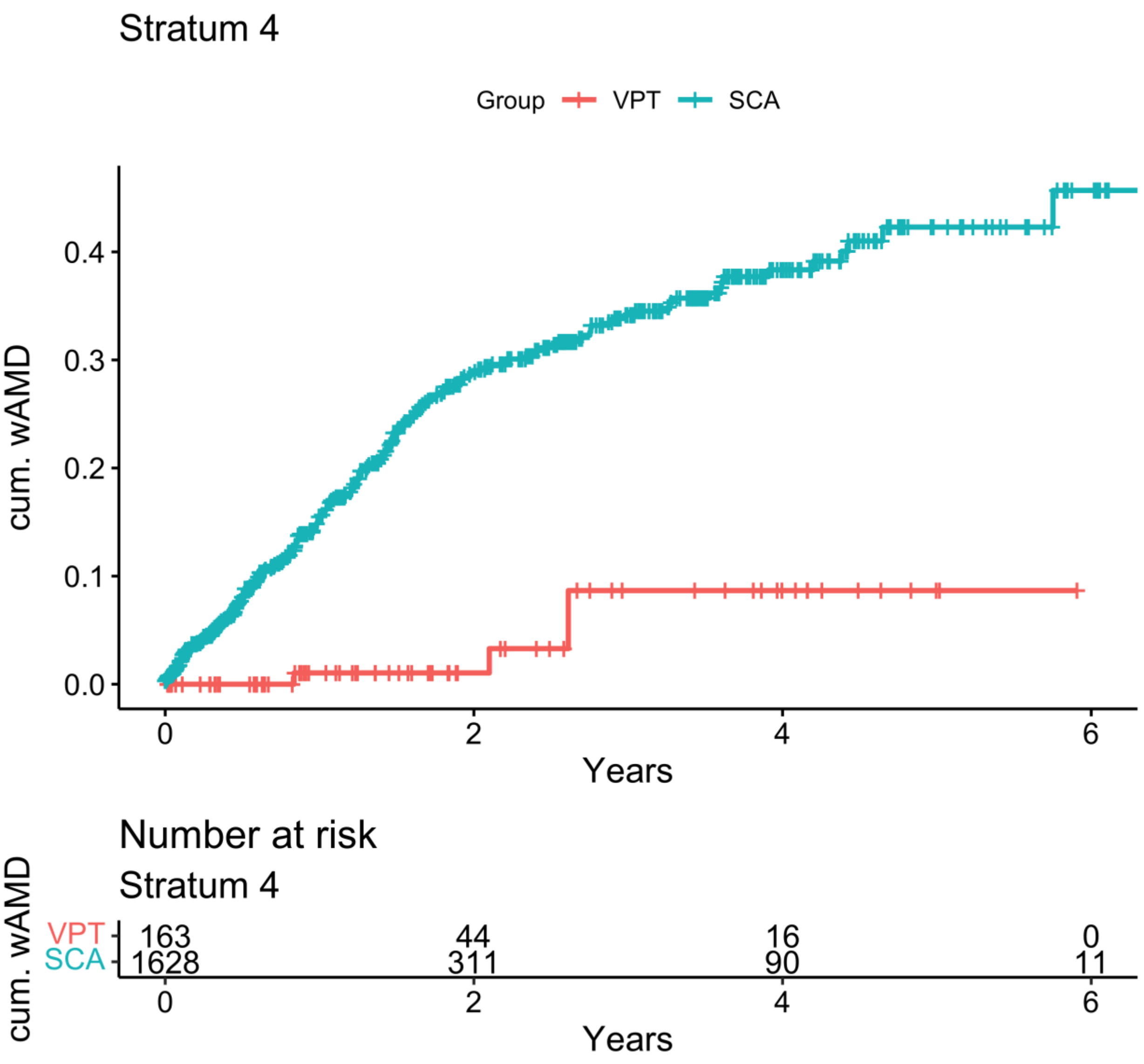

Stratum 4
Group VPT SCA
cum. wAMD
0.0
0.1
0.2
0.3
0.4
0
2
4
6
Years
Number at risk
Stratum 4
cum. wAMD
VPT 163 44 16 0
SCA 1628 311 90 11
0
2
4
6
Years

Stratum 5

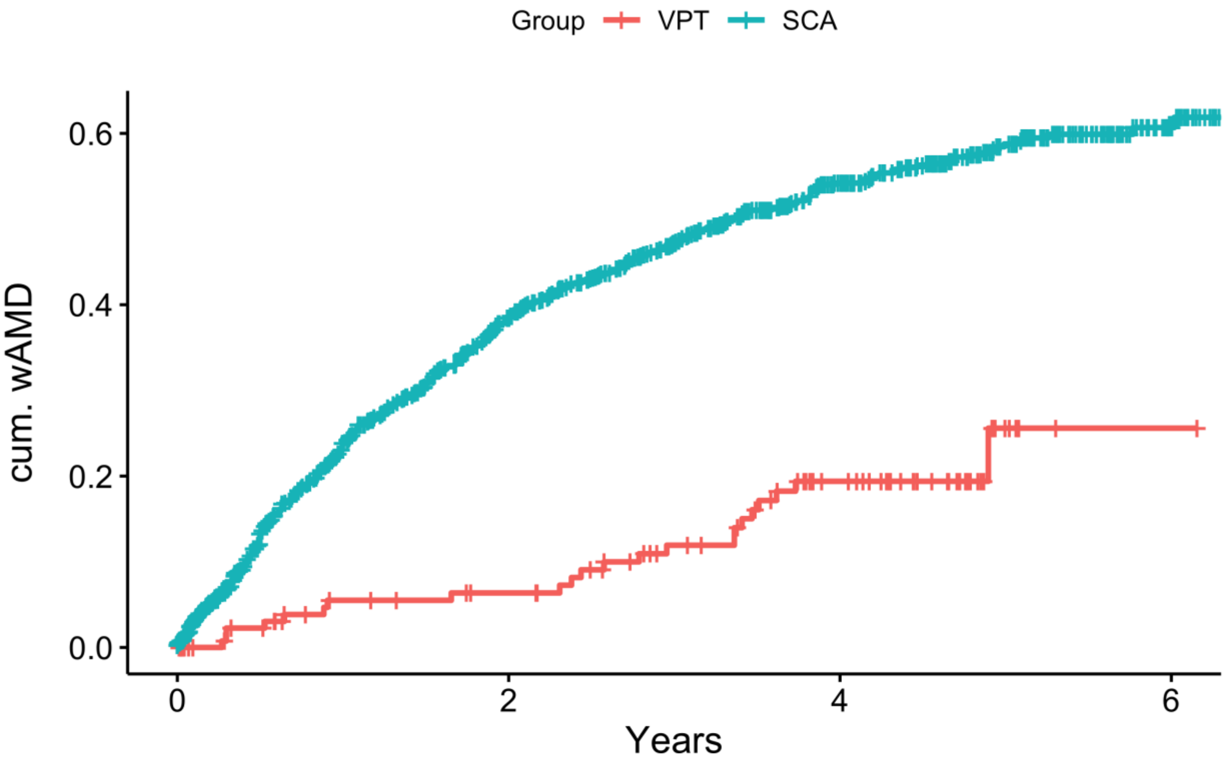

Number at risk

Stratum 5

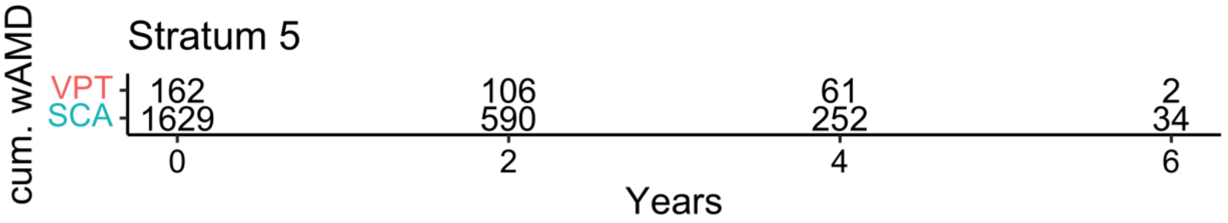

A test for equality of the Kaplan-Meier survival curves (a stratified log-rank test) shows a very significant difference in survival between the VPT and SCA groups.

Table 6. Kaplan-Meier test between Groups, stratified by propensity score quintiles. Chisq = 122.511026 on 1 degrees of freedom, p = 0.000000

	N	Observed	Expected	(O-E)^2/E	(O-E)^2/V
**Analysis.Group=VPT**	814	32	164.5	106.7	122.5
**Analysis.Group=SCA**	8140	1321	1188	14.78	122.5

To allow for inclusion of covariates, and to provide an overall summary of the results, we carried out a Cox proportional hazards regression, again stratified by propensity score quintiles.

Cox PH modelinig initially included all of the covariates included in the propensity score calculation. Non-significant variables were dropped, until the final model included the following important covariates:

- Severity
- Age
- AREDS use
- Analysis Group

Results from the Cox proportional hazards fit are shown in the following Table.

Table 7. Cox PH summary of survival difference, VPT Non-Laser Included, with Encounter Matching. Estimated hazard ratios are in the column labeled exp(coef).

	coef	exp(coef)	se(coef)	z	p
**SeverityUnspecified**	0.7097	2.033	0.1405	5.051	4.389e-07
**SeverityIntermediate**	0.8173	2.264	0.09259	8.828	0
**SeverityNonCentralGA**	0.7401	2.096	0.1291	5.731	9.96e-09
**SeverityCentralGA**	0.6133	1.847	0.1298	4.724	2.314e-06
**Age**	0.01289	1.013	0.003554	3.627	0.0002864
**Areds.FlagYes**	0.4384	1.55	0.06316	6.941	3.883e-12
**Analysis.GroupSCA**	1.764	5.836	0.1792	9.846	0

Likelihood ratio test=372.41 on 7 df, p=0 n= 8954, number of events= 1353

For severity, the “Early” group (used as the reference group in the CPH model) had the highest hazard, with all of the other severity levels producing a hazard ratio < 1. Depending on which of the 4 analysis data sets were used, several other severity levels showed significant increase in hazard over the reference group.

Subjects with AREDS use showed a significant increase in hazard (30%-50%, depending on analysis data set).

For all analysis data sets, there was a significant increase in hazard for the SCA group, of about 5.75 after adjusting for severity, age, and AREDS use (here we have (HR = 5.84, p = <1e-04)).

The following forest plot shows the parameter estimates and confidence intervals from the Cox PH regression.

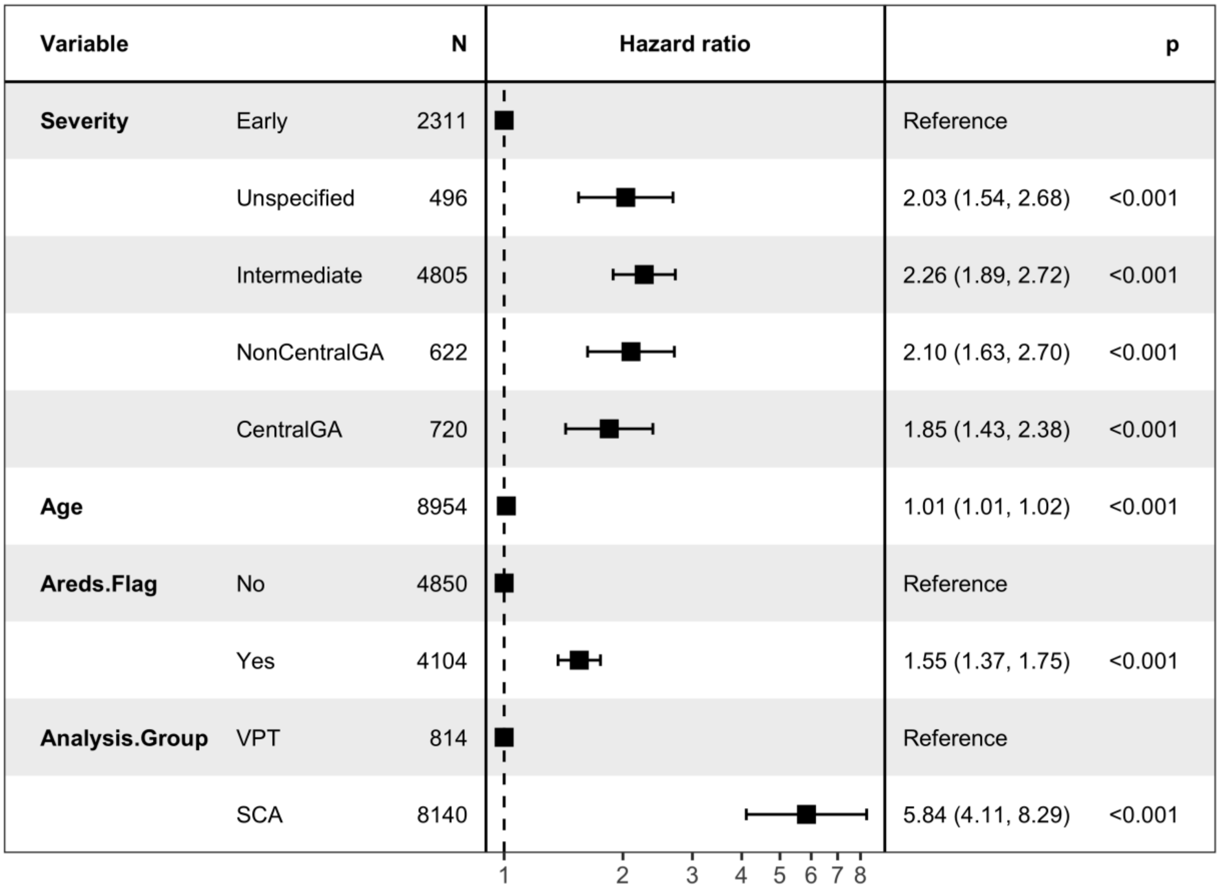

Figure 2. Forest plot of Cox PH results, VPT Non-Laser Included, with Encounter Matching

The test for Analysis Group shows a high level of significance.

Table 8. ANOVA for Cox PH model, VPT Non-Laser Included, with Encounter Matching.

	loglik	Chisq	Df	Pr(>|Chi|)
**NULL**	-8852	NA	NA	NA
**Severity**	-8787	130.3	4	3.322e-27
**Age**	-8781	12.41	1	0.000427
**Areds.Flag**	-8755	51.18	1	8.433e-13
**Analysis.Group**	-8666	178.5	1	1.033e-40

A test for the proportional hazards assumption shows strong evidence of non-proportionality (cox.zph(), p = 0.0234). However (see appendix), various diagnostic plots do not indicate strong non-proportionality in the Cox PH model.

Table 9. Tests for proportional hazards violations., VPT Non-Laser Included, with Encounter Matching.

	chisq	df	p
**Severity**	7.482	4	0.1125
**Age**	0.9195	1	0.3376
**Areds.Flag**	2.204	1	0.1377
**Analysis.Group**	6.197	1	0.0128
**GLOBAL**	16.2	7	0.02336

Summary of Survival Fits, VPT Non-Laser Included, with Encounter Matching

The following plot shows the overall cumulative wet AMD conversion probabilities by group, ignoring covariates.

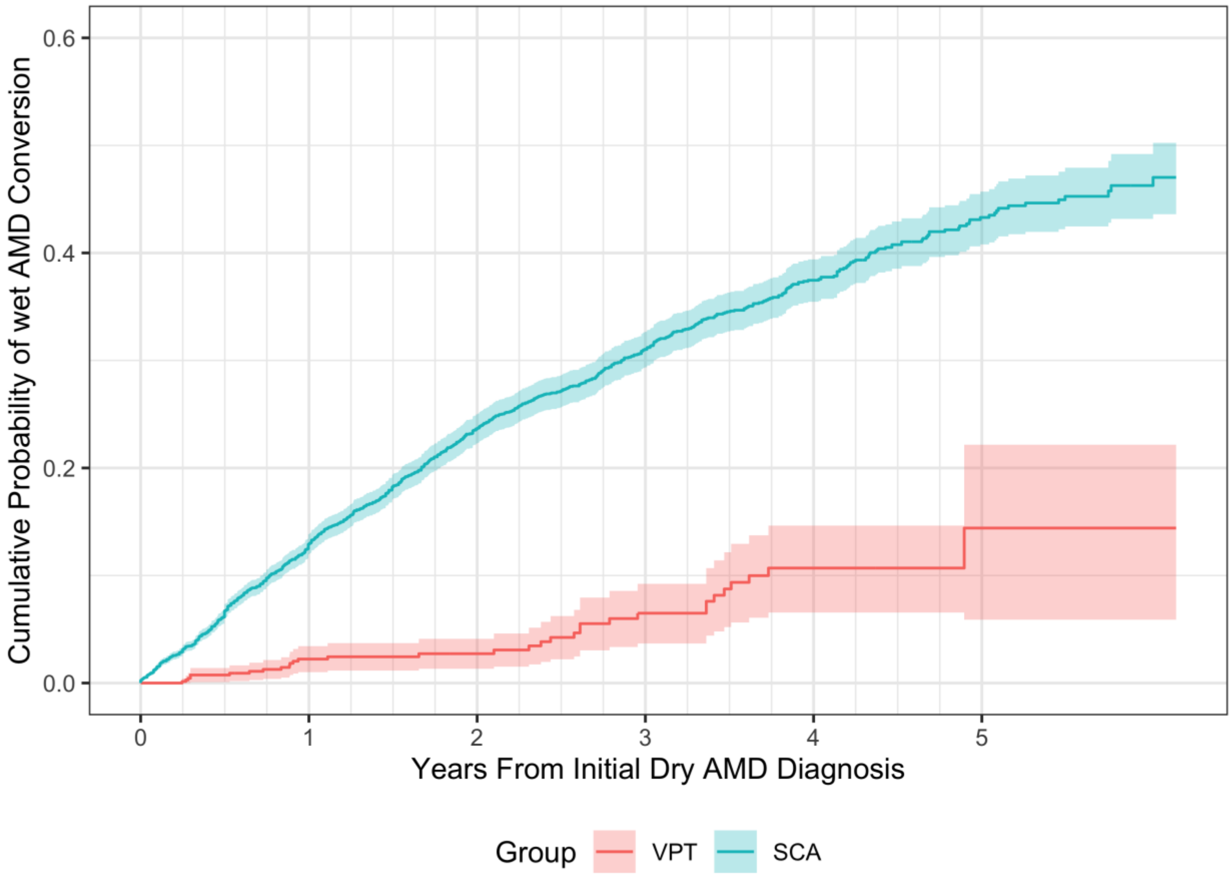

Figure 3. Overall Kaplan-Meier cumulative wet AMD conversion probability by group, VPT Non-Laser Included, with Encounter Matching. Shaded areas indicate 95% confidence intervals.

The following Table shows the cumulative probability of progressing to wet AMD, by year and group.

Table 10. Summary of overall survival by group (unstratified Kaplan-Meier estimates), VPT Non-Laser Included, with Encounter Matching.

Analysis Group	Years From DAMD Diagnosis	n at risk	n events	Cumulative Probability of wet AMD	95% CI
VPT	1	483	13	2.2%	[1.0%, 3.4%]
	2	289	2	2.7%	[1.3%, 4.1%]
	3	185	9	6.5%	[3.7%, 9.2%]
	4	108	7	10.7%	[6.6%, 14.6%]
SCA	1	3348	677	12.9%	[12.0%, 13.9%]
	2	1989	338	23.6%	[22.3%, 25.0%]
	3	1197	160	31.0%	[29.4%, 32.7%]
	4	653	90	37.5%	[35.5%, 39.4%]

The hazard ratio between the two groups is summarized in the following Table. Since there are multiple eyes per person, a clustered bootstrap (clustered by subject) was used to provide a robust check on the confidence interval. The lower bound on the 95% confidence interval for the hazard ratio is above 4 using either method, again providing strong evidence for a hazard ratio greater than 1.

Table 11. Cox proportional hazards estimated hazard ratio and associated confidence intervals, VPT Non-Laser Included, with Encounter Matching. Cox PH model is stratified by propensity score quartiles.

Estimated Hazard Ratio	95% CI (asymptotic)	95% CI (bootstrap[1])
5.8	[4.1, 8.3]	[4.1, 8.8]

1-Bootstrap confidence interval is based on 100 cluster (subject level) bootstrap samples.

Visual Acuity, VPT Non-Laser Included, with Encounter Matching

Visual acuity (ETDRS letters or the equivalent) was measured for a subset of subject visits (usually non-treatment visits). The SCA group averaged about 714.1 VA measurements per month, the VPT group averaged 52.3.

A tabulation of the mean VA by year shows a slight downward trend for the SCA group (perhaps due to aging?) but no obvious differences between the two groups.

Table 12. Mean visual acuity (ETDRS letters or equivalent) by Group and Year, VPT Non-Laser Included, with Encounter Matching. Cox PH model is stratified by propensity score quartiles.

Analysis Group	Mean VA 2017	Mean VA 2018	Mean VA 2019	Mean VA 2020	Mean VA 2021	Mean VA 2022
VPT	69.3	67.2	68.4	70.4	68.3	67.6
SCA	67.9	68.0	66.9	66.0	64.3	63.8

The following plot shows the mean VA per month for the SCA and VPT subjects. As expected there is more noise in the much smaller VPT group. Given the amount of noise it is difficult to assess whether there are any differences in VA through time.

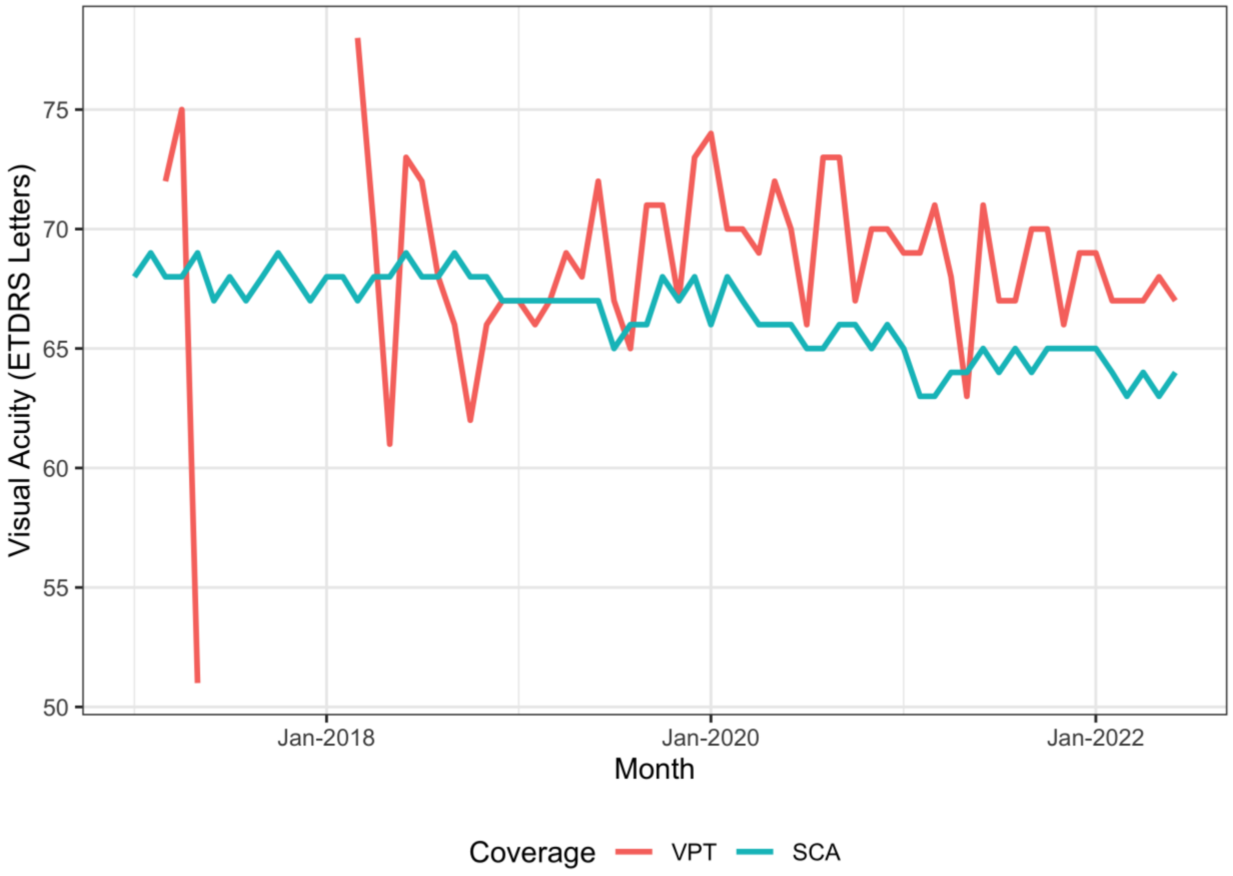

Figure 4. Visual Acuity by month, SCA, VPT Groups, VPT Non-Laser Included, with Encounter Matching.

A loess smoother shows a difference between the two groups during 2020-2021, perhaps due to the COVID pandemic?

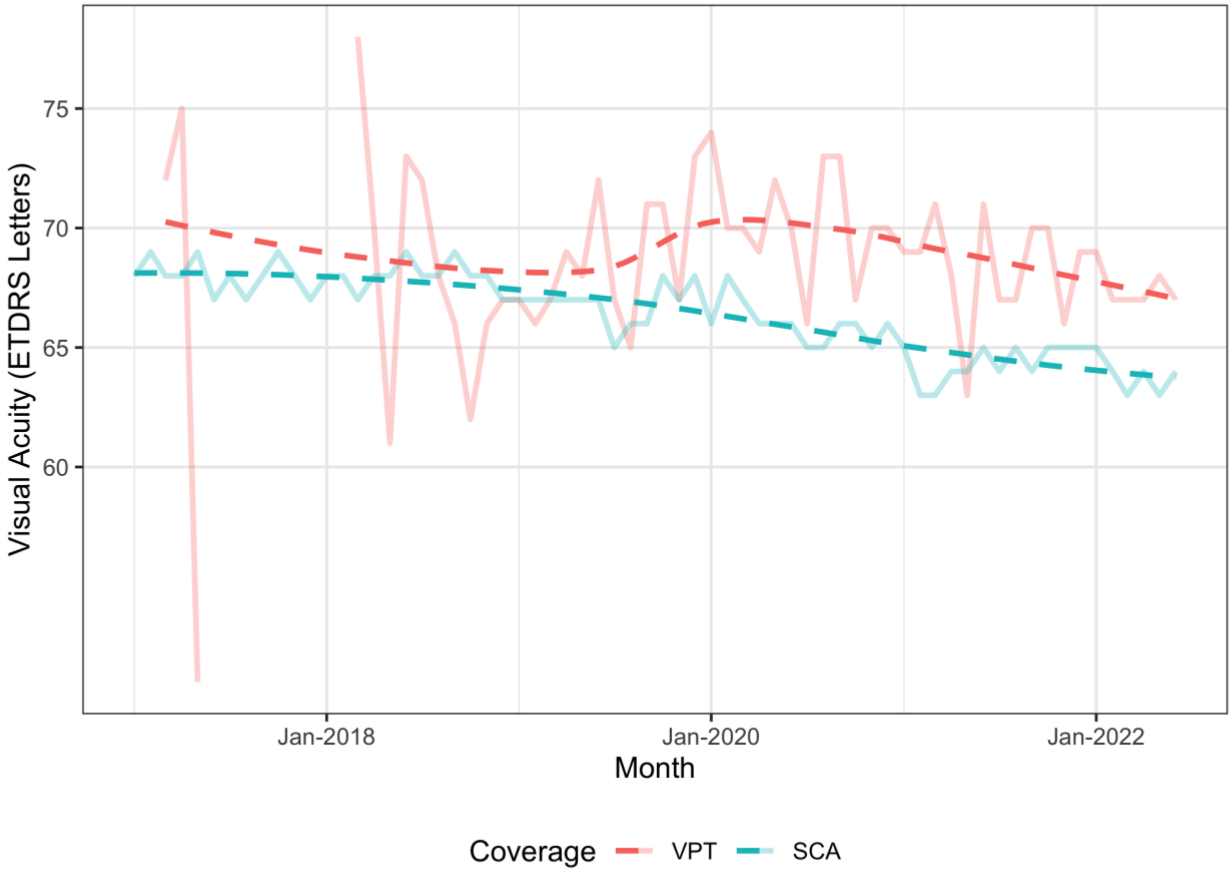

Figure 5. Visual Acuity by month, SCA, VPT Groups, with loess smooths., VPT Non-Laser Included, with Encounter Matching.

Caveats for Propensity Score Analysis

Potential shortcomings of this propensity score analysis include:

- The VPT group only includes subjects from a single practice, thus the generalizability of the results may be in question.
- Propensity score methods can "balance" the two groups with respect to the variables used in the propensity score model. However, they do not balance for unmeasured covariates, thus if there are other important predictors of group membership or outcome that are missing the results could be misleading.
- This analysis used subjects for whom the latest ICD-10 coding was used, which added Dry AMD severity codes not present in the earlier ICD-9. We are thus using the site-level assessment of AMD severity and assume a reasonably consistent diagnostic judgement across sites. There is no reading center or verification of the coding as would be implemented in a clinical trial.
- The ICD-10 coding used in the Vestrum database may not capture all subject outcomes, including potentially some adverse events.
- There are inconsistencies in ICD coding, for example some subjects who had a series of anti-VEGF injections were never coded as having converted to wet AMD. Thus an eye was considered converted to wet AMD if the appropriate ICD code was entered **and** at least one anti-VEGF injection was administered. The conversion date was set to earliest of the ICD coding date or the date of the first anti-VEGF injection. Alternative definitions of conversion do not change the basic conclusions.
- There are differences in follow-up time and number of encounters (reported subject-physician interactions) between the two groups, with the VPT group having more encounters and longer follow-up. As these are "post-randomization" outcomes they were not used in the initial propensity score matching. The longer follow-up in the VPT group most likely produced more events, whereas the effect of increased encounters, if any, is unknown. Thus if there is any bias due to follow-up it is likely to produce a smaller hazard ratio for SCA versus VPT.
- To match follow-up intensity between the two groups a second propensity score analysis used the mean time-between-visits for each subject as an additonal matching variable. This produces a better correspondence in follow-up time and number of visits between the two groups at the expense of potential bias issues with using post-diagnosis information in the matching.

Appendix/Supplemental

This appendix includes diagnostic plots and alternative analyses.

Distribution of Follow-Up Time

The following plot shows the follow-up time by group and wet AMD status.

Note the spikes in the SCA group at approximately 3 months, 6 months, 1 year, 2 years, and 3 years of follow-up.

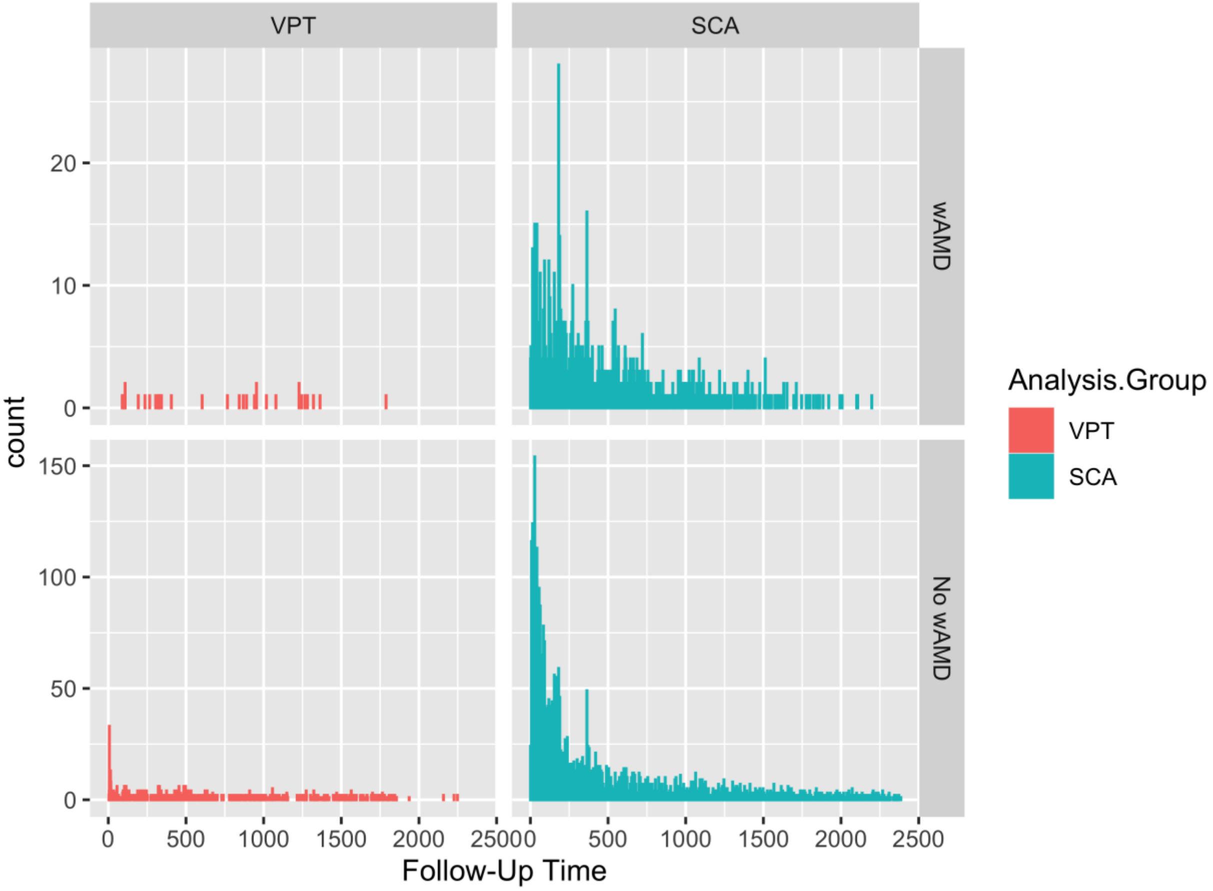

Figure 6. Follow up time by study group and wAMD conversion status. Subjects with 0 days follow-up are excluded.

Diagnostic Plots for Propensity Scores

Propensity score diagnostic plots look good overall. The overall distributions for the propensity scores are quite similar.

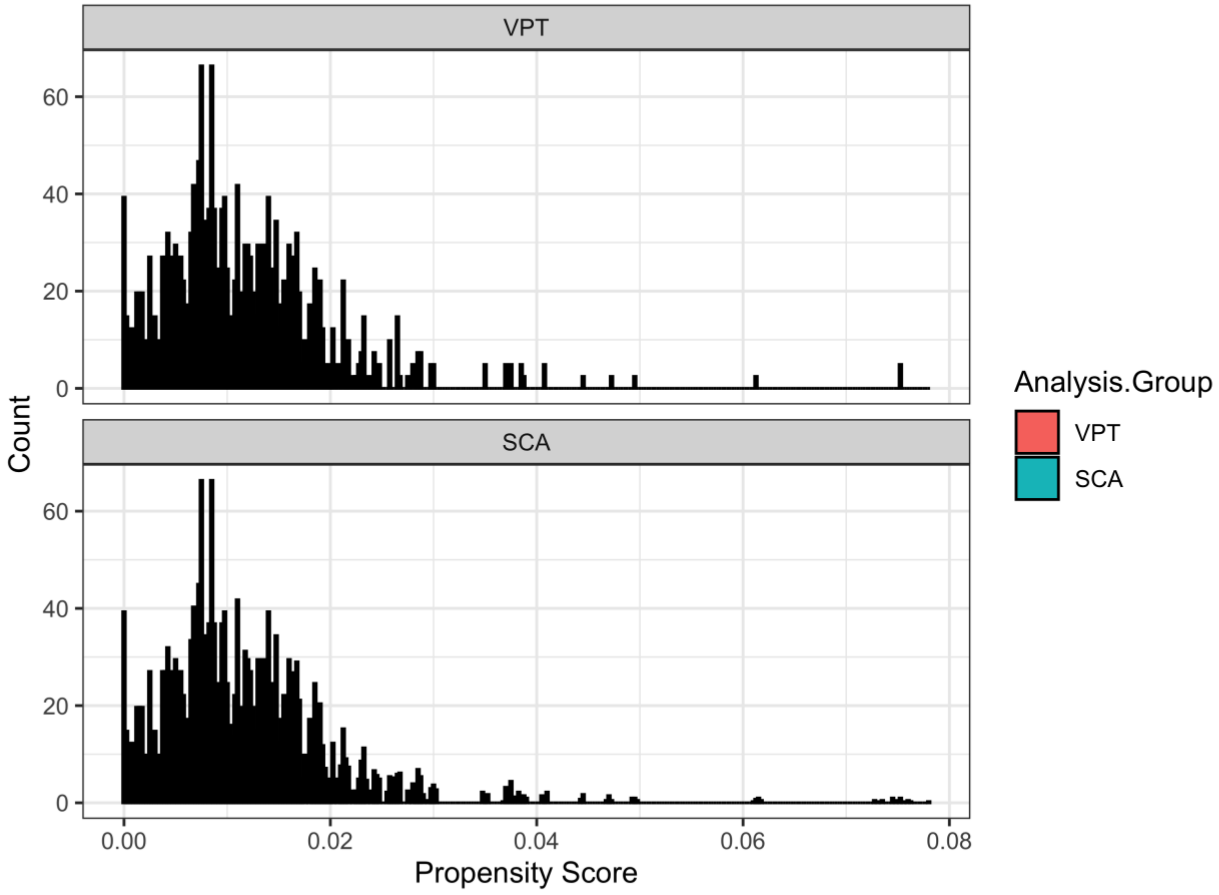

Figure 7. Diagnostic plots for propensity scores, overall distribution.

Propensity scores are also similar within each PS stratum.

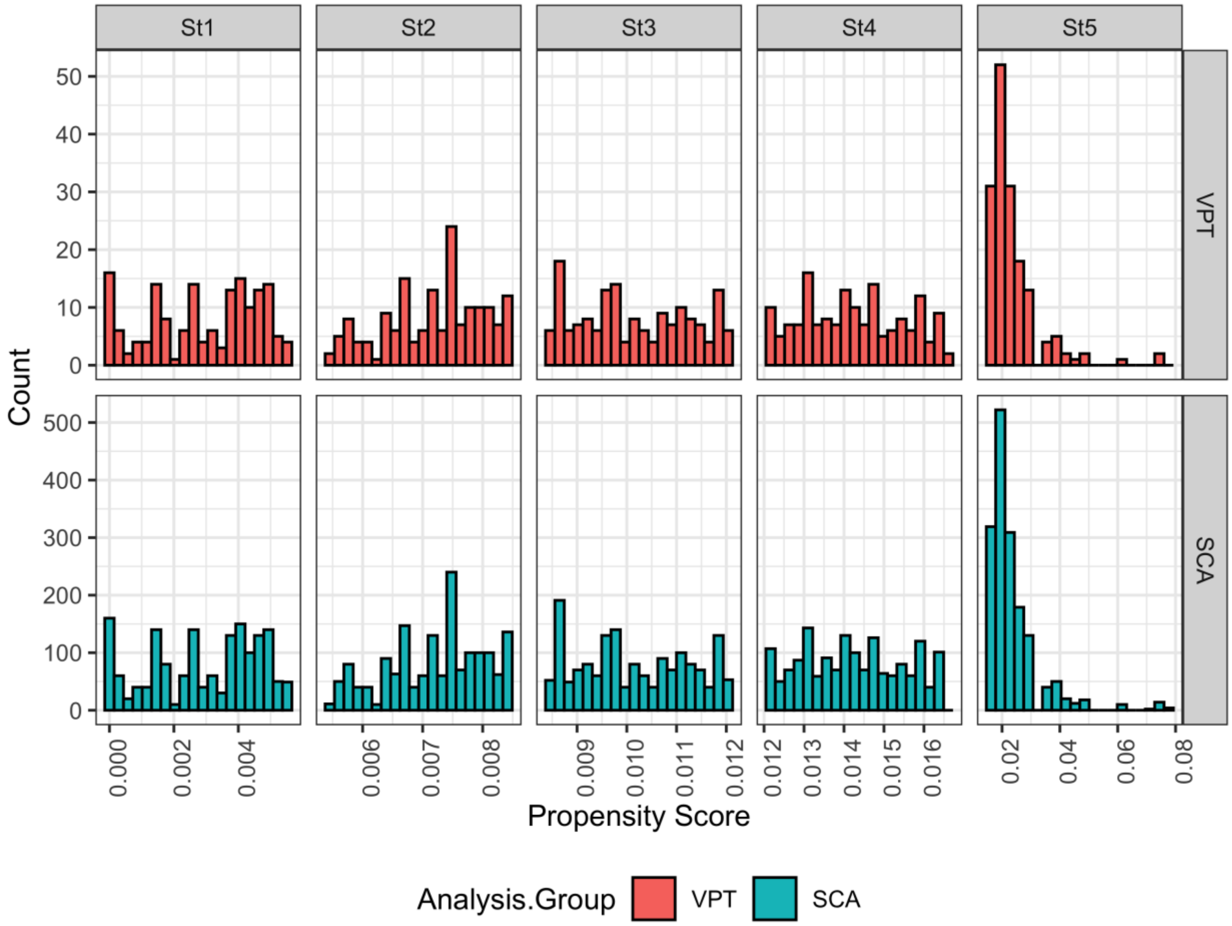

Figure 8. Diagnostic plots for propensity scores, distribution by PS stratum.

Each individual component also looks good. What we want to see is that within each stratum the two groups are similar (e.g. the pairs of orange and yellow bars have good overlap, the pairs of green bars are similar height).

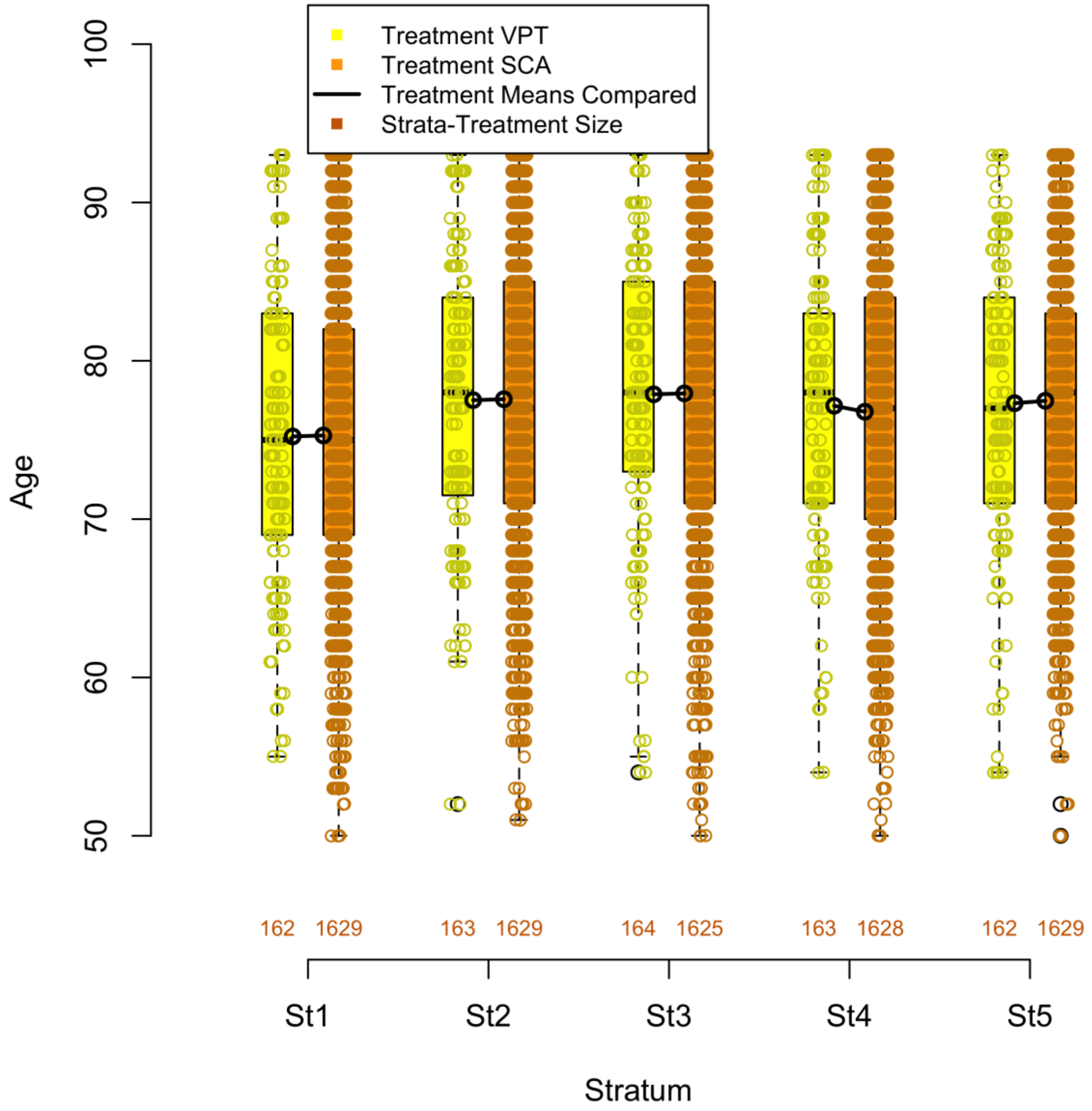

Figure 9. Diagnostic plots for propensity scores, age.

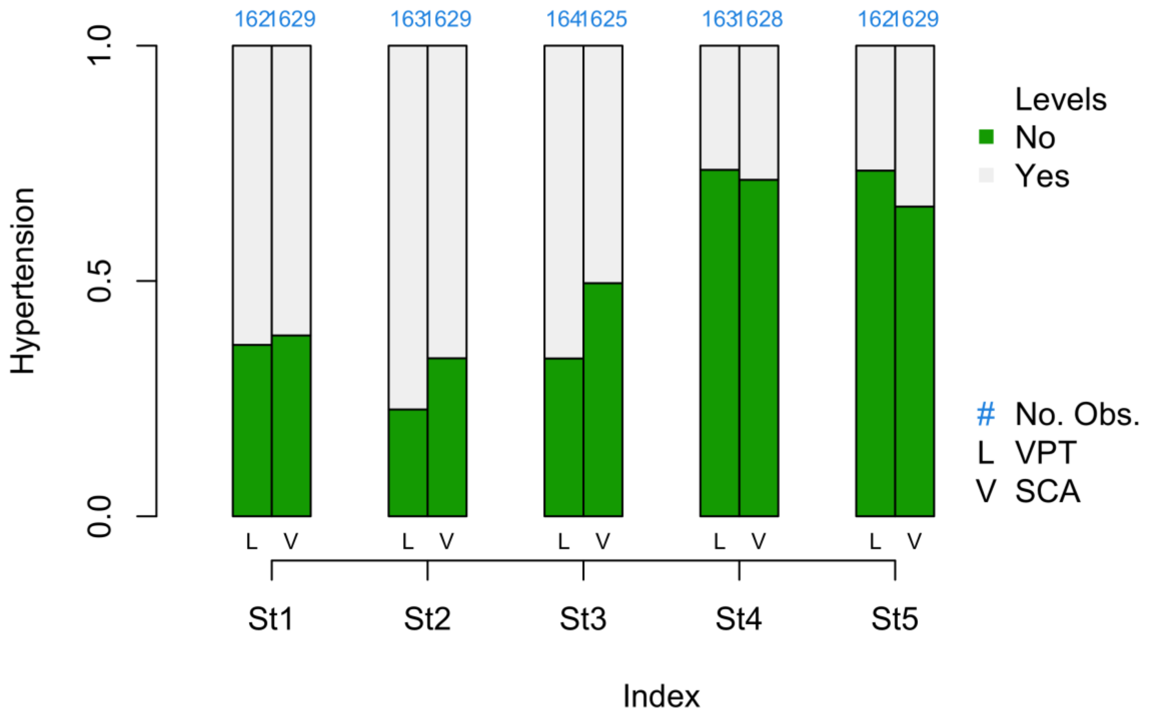

Figure 10. Diagnostic plots for propensity scores, hypertension.

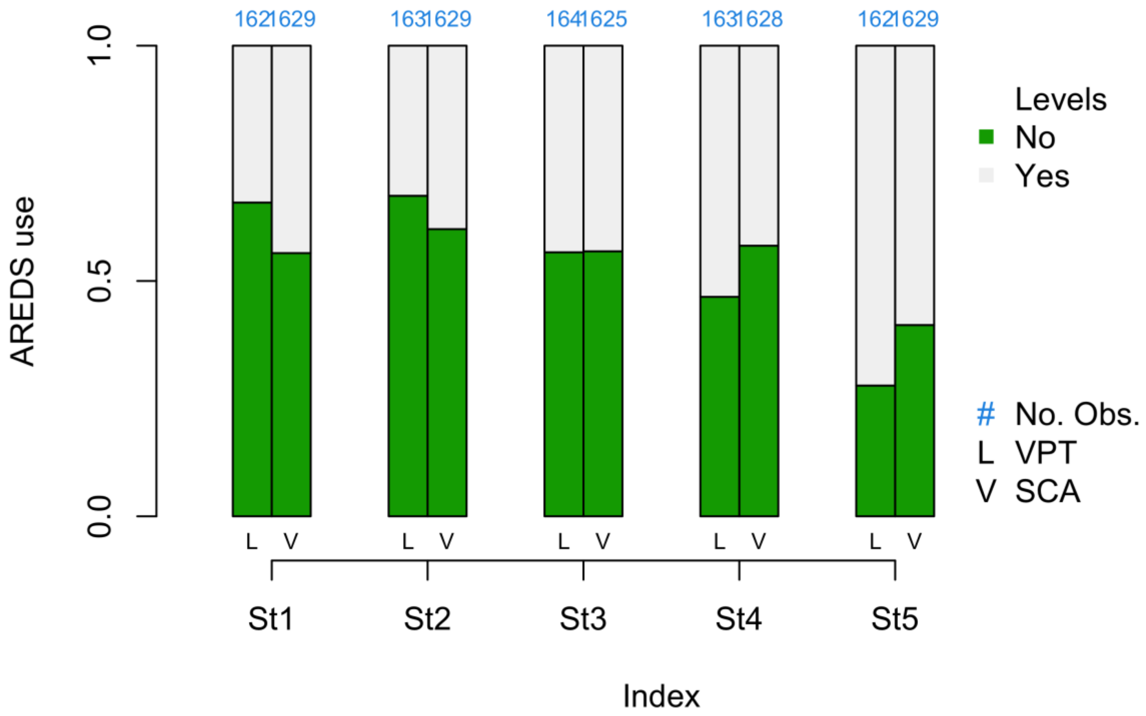

Figure 11. Diagnostic plots for propensity scores, AREDS use.

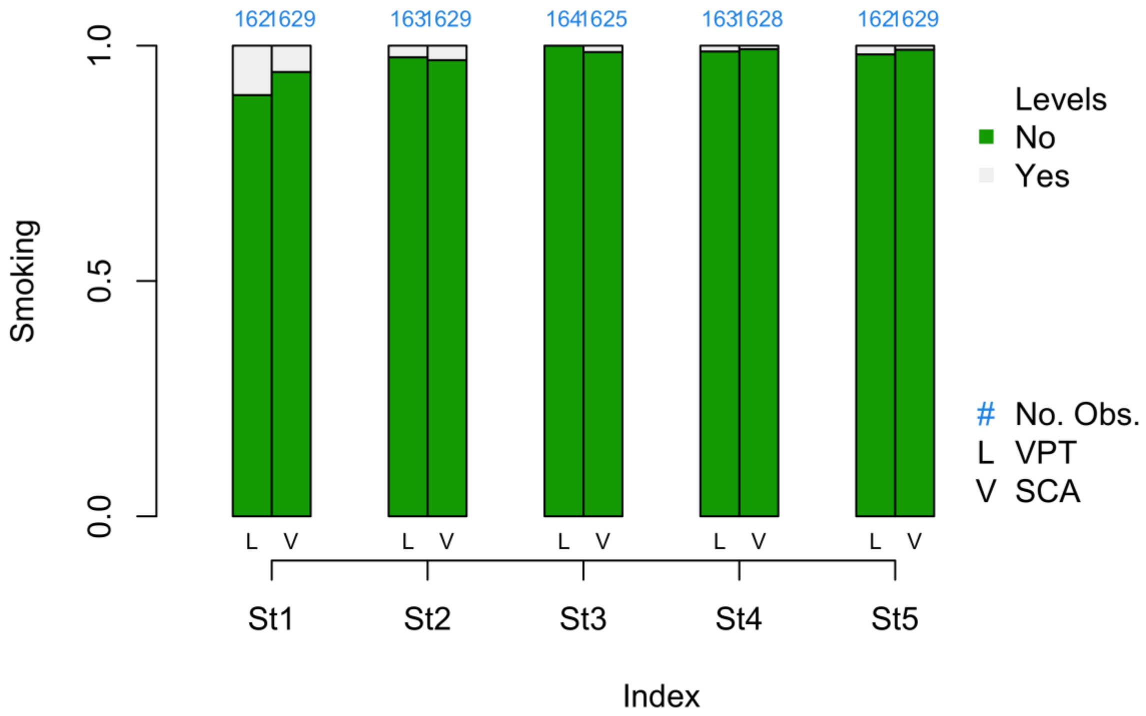

Figure 12. Diagnostic plots for propensity scores, smoking.

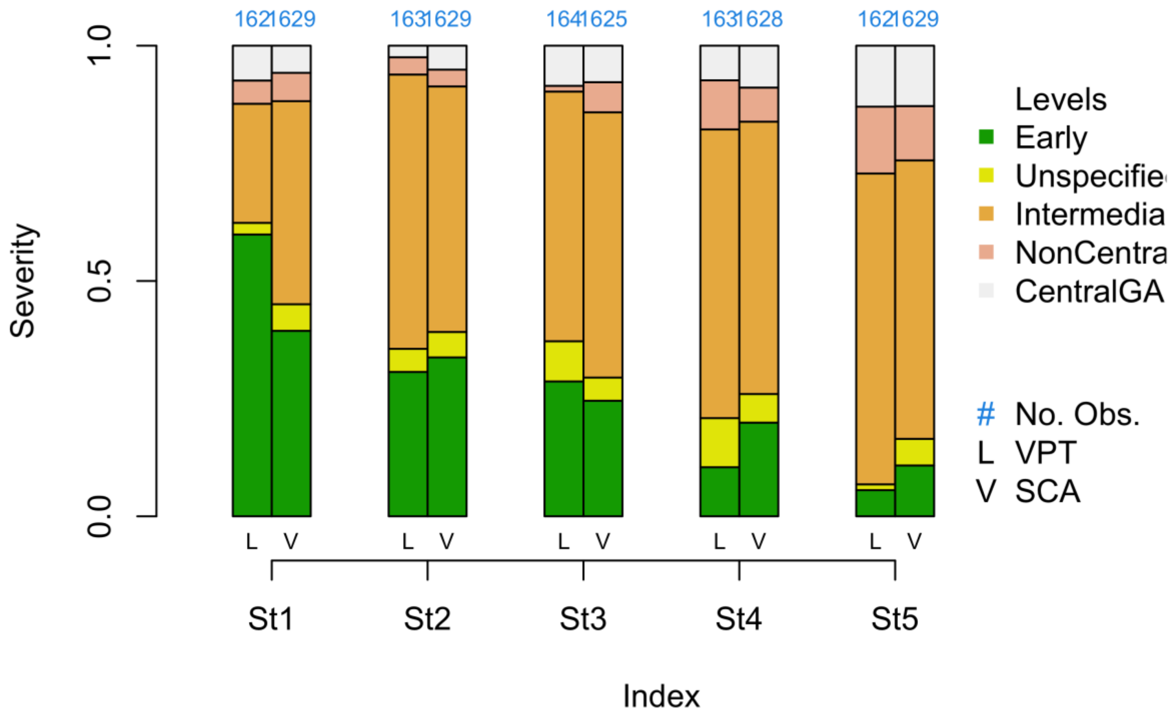

Figure 13. Diagnostic plots for propensity scores, severity

Incidence Rates via Poisson Regression

As a simpler alternative to the survival analysis, we can fit a poisson regression model. Poission regression adjusts for the follow-up on each eye individually, so corrects the bias in incidence rates somewhat. We can also easily account for the propensity score strata.

Fitting generalized (poisson/log) linear model: Converted.to.wAMD.n ~ Analysis.Group + PSStratum + offset(Follow.up.Years)

	Estimate	Std. Error	z value	Pr(>|z|)
**(Intercept)**	-7.737	0.2091	-37	1.169e-299
**Analysis.GroupSCA**	1.575	0.179	8.797	1.411e-18
**PSStratumSt2**	1.449	0.1364	10.63	2.252e-26
**PSStratumSt3**	1.632	0.1305	12.51	6.934e-36
**PSStratumSt4**	1.599	0.13	12.3	8.839e-35
**PSStratumSt5**	1.534	0.1202	12.77	2.451e-37

Table 13. Incidence rates from poisson regression (correcting for unequal follow-up in data, and adjusting for stratum differences.)

Analysis.Group	incidence rate	std.error	df	null	statistic	p.value
VPT	0.004109	0.0007331	Inf	1	-30.8	2.87e-208
SCA	0.01984	0.0006741	Inf	1	-115.4	0

The incidence rate ratio from this fit is 4.8.

Diagnostic Plots for Cox PH Model

The following plots are diagnostics from the Cox proportional hazards fit.

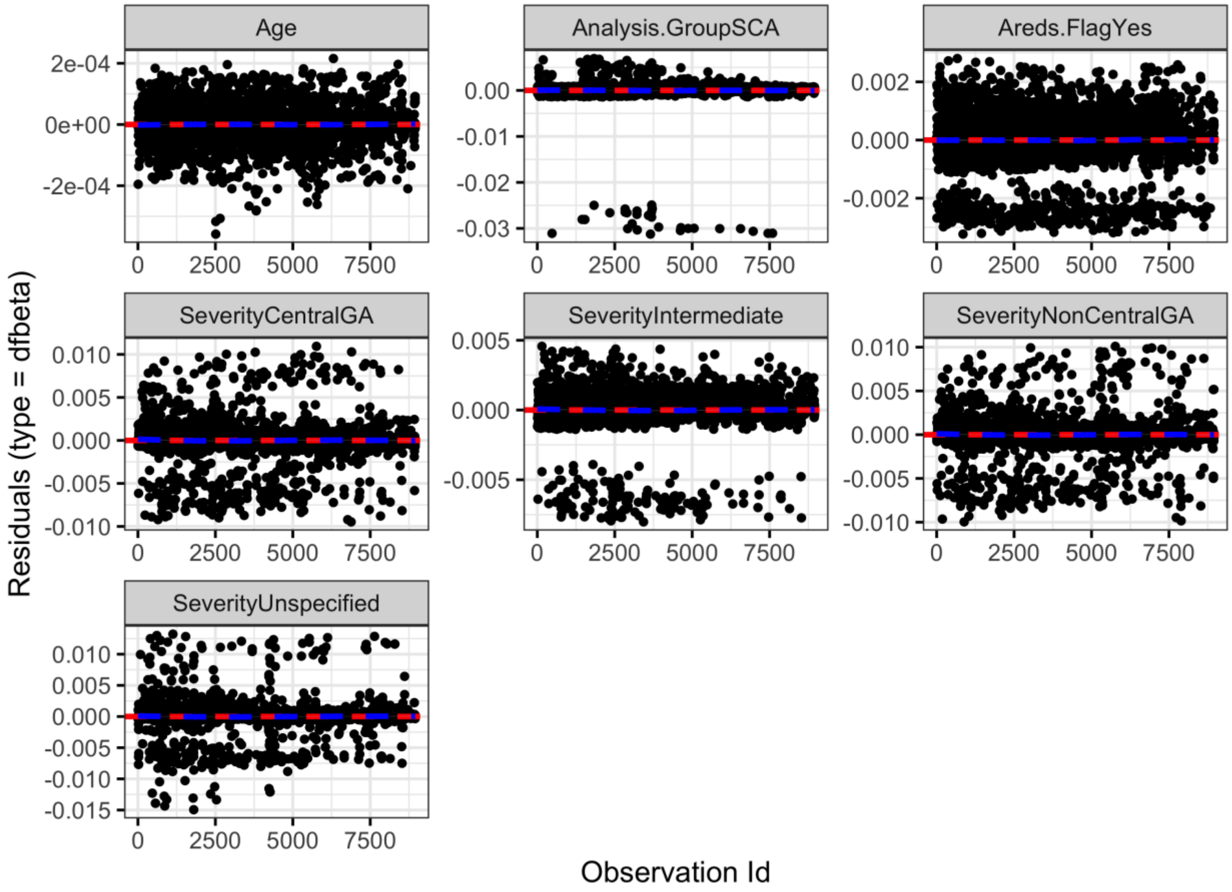

Figure 14. Dfbeta residuals from Cox PH fit.

```
## `geom_smooth()` using formula = 'y ~ x'
```

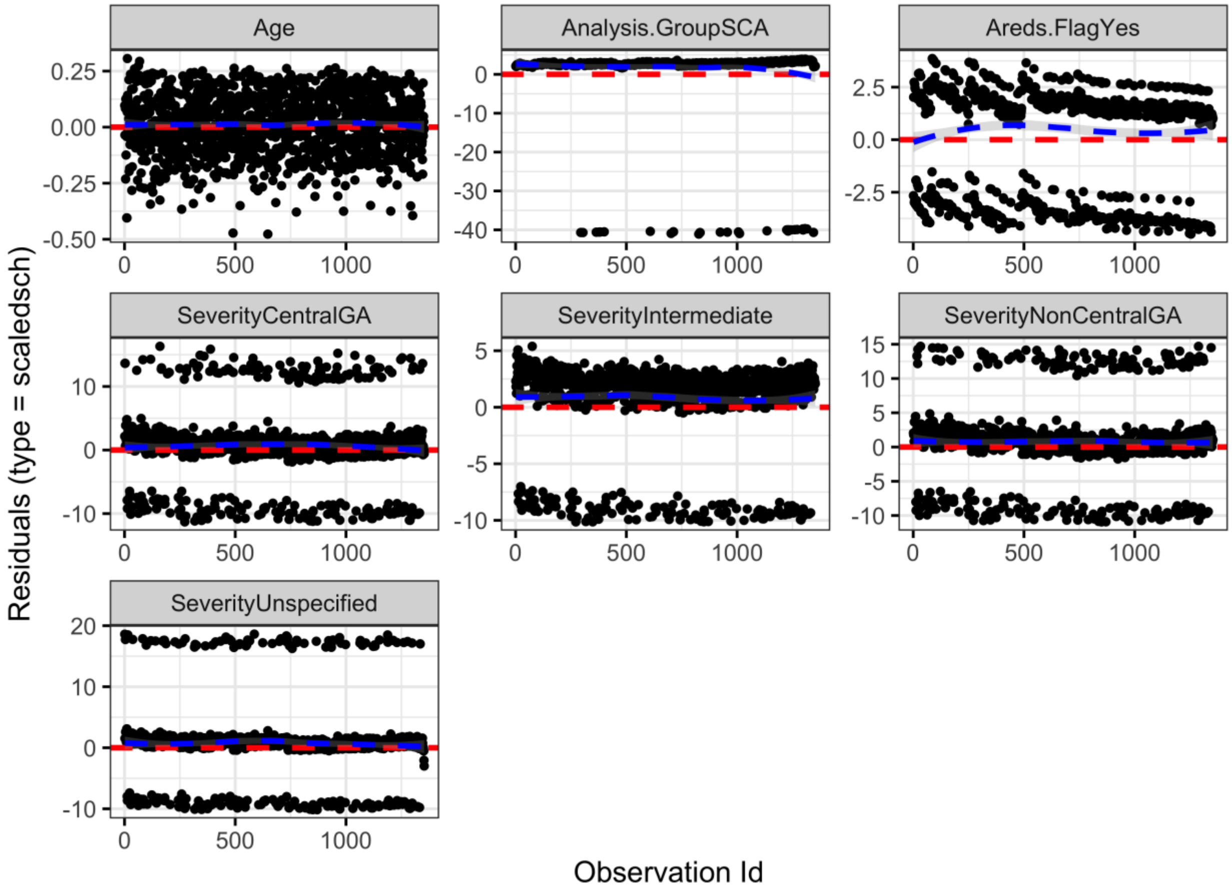

Figure 15. Scaled Schoenfeld residuals vs Time.

Retinal Protection Sciences

Comparison of SDM treatment to standard of care Propensity matched Vestrum data, with AMD severity coding v6.0

[with non-laser-treated VPT subjects] [without encounter matching]

Gerry Gray, Regulatory Pathways, Inc.

9/20/2023

Table of Contents

INTRODUCTION **98**
BASELINE TABULATIONS, VPT NON-LASER INCLUDED, NO ENCOUNTER MATCHING **100**
TREATMENT AND RAW OUTCOMES, VPT NON-LASER INCLUDED, NO ENCOUNTER MATCHING **101**
SURVIVAL ANALYSIS BY PS STRATUM, VPT NON-LASER INCLUDED, NO ENCOUNTER MATCHING **103**
SUMMARY OF SURVIVAL FITS, VPT NON-LASER INCLUDED, NO ENCOUNTER MATCHING **112**
VISUAL ACUITY, VPT NON-LASER INCLUDED, NO ENCOUNTER MATCHING **113**
CAVEATS FOR PROPENSITY SCORE ANALYSIS **117**
APPENDIX/SUPPLEMENTAL **118**
DISTRIBUTION OF FOLLOW-UP TIME 118
DIAGNOSTIC PLOTS FOR PROPENSITY SCORES 119
INCIDENCE RATES VIA POISSON REGRESSION 126
DIAGNOSTIC PLOTS FOR COX PH MODEL 127

Introduction

This report contains analyses of propensity score matched data from the Vestrum database.

This is the second propensity score analysis from this database. One of the major perceived shortcomings of the previous analysis was that AMD severity at diagnosis was not available in the then-current ICD-9 coding. This issue was addressed in the new ICD-10 codes, which indicate AMD severity at diagnosis. This new information on initial AMD severity was used in the revised propensity score matching.

This analysis includes severity of the initial AMD diagnosis, classified as "Early", "Unspecified", "Intermediate", "Non-Central GA" or "Central GA".

There are four separate versions of this report, created to satisfy a reviewer's request, to adhere to a principle of "no post-randomization exclusions" of subjects, and to evaluate the sensitivity of the analyses to differential follow-up intensity. The four versions are the combinations of the following:

SDM Laser Treatment

11. Including all subjects from the VPT group who met the initial filtering (I/E) criteria.
12. Including only VPT subjects who were treated with the SDM laser (a "post-randomization" event) in addition to meeting the I/E criteria.

Follow-up Intensity

13. Propensity score matching that includes follow-up intensity, using using the mean time between visits.
14. Propensity score matching that does not include follow-up intensity.

Throughout, we use the labels "VPT" to indicate Luttrull subjects, treated with standard of care and SDM laser as appropriate, and "SCA" to indicate the matched eyes from the Vestrum database.

The Vestrum database of ~500,00 eyes with visits between 1/2/2017 and 7/31/2023 were initially filtered using study inclusion/exclusion criteria to obtain a candidate set of ~200,000 eyes, including 814 VPT eyes (737 who were treated with SDM Laser). Although exclusion of VPT eyes based on treatment recieved during the study violates a statisical principle of "no post-randomization exclusions", at the insistence of reviewers we analyzed data both with and without VPT SDM laser treated eyes.

After completion of the initial filtering, and for both 1) all VPT eyes and 2) only SDM laser-treated VPT eyes, nearest-neighbor propensity score matching was used to obtain a matched set of control eyes from the SCA group. The R Matchit package was used for the matching (R version 4.0.2, Matchit version 3.0.2).

Propensity scores were based on the following covariates:

Table 1. Variables used to perform propensity score matching

Variable
Age
Smoking status
AREDS vitamin use status
Hypertension status
AMD Severity

To obtain similar follow-up intensity between the groups, a second propensity score matching used all of the above variables plus the mean time-between-visits. These analyses are labeled “with encounter matching” in the headings.

An earlier analysis of data from the Vestrum database was reported in REF. At the time of that analysis, the Vestrum system used the then-current ICD-9 codes for AMD.

With the transition to ICD-10, the codes for AMD now include separate categories for disease severity.

Table 2. ICD 10 AMD Severity codings.

AMD Severity	ICD Code	Description
Early	H35.31X1	early dry AMD—a combination of multiple small drusen (≤ 63 µm), few intermediate drusen (> 63 µm and ≤ 124 µm), or retinal pigment epithelium (RPE) abnormalities
Intermediate	H35.31X2	intermediate dry AMD—extensive intermediate drusen (> 63 µm and ≤ 124 µm) or at least 1 large drusen (≥ 125 µm
NonCentral GA	H35.31X3	advanced atrophic dry AMD without subfoveal involvement—geographic atrophy (GA) not involving the center of the fovea
Central GA	H35.31X4	advanced atrophic dry AMD with subfoveal involvement—GA involving the center of the fovea
Unspecified	H35.31X0, H35.31,H35.312,H35.311,H35.313	

Diagnostics from the matching indicated a good overlap of propensity scores between the two groups (see appendix).

Eyes were considered to have “converted” to wet AMD during the follow-up period if both of the following occurred:

- an ICD code for wet AMD was entered into the database
- anti-VEGF injections were initiated

The time of wet AMD conversion was the earliest of the date where the ICD code was entered or the date of the first anti-VEGF injection.

Baseline Tabulations, VPT Non-Laser Included, no Encounter Matching

The following Table summarizes the demographics of the two Groups in this analysis.

Table 3. Demographics by study group, after propensity score matching.

**Factor**<br>Level	VPT	SCA
**N (study eyes)**	814	8140
**N (subjects)**	441	5115
**Gender**		
Female	275/441 (62.4%)	3013/5115 (58.9%)
Male	166/441 (37.6%)	2057/5115 (40.2%)
Other	0/441 (0.0%)	45/5115 (0.9%)
**Age (years)**		
Mean(SD)	77.2 (9.2)	77.5 (9.2)
Median	77.0	78.0
Min, Max	[52.0, 93.0]	[50.0, 93.0]
**Age (category)**		
Age: [50,65]	41/441 (9.3%)	534/5115 (10.4%)
Age: (65,70]	61/441 (13.8%)	619/5115 (12.1%)
Age: (70,75]	88/441 (20.0%)	968/5115 (18.9%)
Age: (75,80]	81/441 (18.4%)	1025/5115 (20.0%)
Age: (80,85]	76/441 (17.2%)	861/5115 (16.8%)
Age: (85,90]	55/441 (12.5%)	569/5115 (11.1%)
Age: (90,110]	39/441 (8.8%)	539/5115 (10.5%)
**Hypertension**		
No	207/441 (46.9%)	2447/5115 (47.8%)
Yes	234/441 (53.1%)	2668/5115 (52.2%)
**AREDS use**		
No	235/441 (53.3%)	2738/5115 (53.5%)
Yes	206/441 (46.7%)	2377/5115 (46.5%)
**Smoking**		
No	427/441 (96.8%)	4978/5115 (97.3%)
Yes	14/441 (3.2%)	137/5115 (2.7%)
**AMD Severity**		
Early	123/441 (27.9%)	1271/5115 (24.8%)
Unspecified	23/441 (5.2%)	285/5115 (5.6%)
Intermediate	239/441 (54.2%)	2766/5115 (54.1%)
NonCentralGA	41/441 (9.3%)	390/5115 (7.6%)
CentralGA	39/441 (8.8%)	450/5115 (8.8%)

Treatment and Raw Outcomes, VPT Non-Laser Included, no Encounter Matching

The following Table summarizes followup and AMD treatments received.

Table 4. Follow-up and treatment summary by study group, after propensity score matching.

**Factor**<br>Level	VPT	SCA
**N (study eyes)**	814	8140
**Total Follow-up Days**		
Mean(SD)	658.2 (571.3)	451.3 (544.9)
Median	486.0	196.0
Min, Max	[4.0, 2248.0]	[0.0, 2392.0]
**Follow Up Years (categories)**		
0 ≤ Follow Up Yrs ≤ 1	331/814 (40.7%)	5415/8140 (66.5%)
1 < Follow Up Yrs ≤ 2	194/814 (23.8%)	1289/8140 (15.8%)
Follow Up Yrs > 2	274/814 (33.7%)	1297/8140 (15.9%)
**Number of Encounters**		
Mean(SD)	12.4 (10.8)	8.4 (9.6)
Median	9.0	5.0
Min, Max	[2.0, 53.0]	[2.0, 93.0]
**Number of anti-VEGF injections per eye**		
Mean(SD)	0.125 (0.795)	1.479 (5.308)
Median	0.000	0.000
Min, Max	[0.000, 11.000]	[0.000, 64.000]
**Treated with SDM Laser**		
No	77/814 (9.5%)	N/A
Yes	737/814 (90.5%)	N/A
**Number of Laser Treatments**		
Mean(SD)	6.3 (5.0)	N/A
n	737	N/A
Min, Median, Max	1, 5.0, 38	N/A
**Converted to wAMD**		N/A
Yes	32/814 (3.9%)	934/8140 (11.5%)
No	782/814 (96.1%)	7206/8140 (88.5%)

There were a total of 32 eyes (3.9%) in 26 subjects in the VPT group, and 934 eyes (11.5%) in 784 subjects in the SCA group that converted to wet AMD. The following Table summarizes the anti-VEGF treatments received by those eyes after conversion.

Table 5. Follow-up and treatment summary after wet AMD conversion, by study group.

**Factor**<br>Level	VPT	SCA
**N (eyes converted to wAMD)**	32	934
**wAMD Follow up Days per Eye**		
Mean(SD)	413.8 (376.1)	693.1 (512.7)
Median	298.5	578.0
Min, Max	[0.0, 1270.0]	[0.0, 2334.0]
**Number of anti-VEGF Injections per wAMD Eye**		
Mean(SD)	3.2 (2.5)	12.6 (10.1)
Median	2.0	10.0
Min, Max	[1.0, 11.0]	[1.0, 64.0]

Survival analysis by PS Stratum, VPT Non-Laser Included, no Encounter Matching

For conversion to wet AMD, the most appropriate method of analysis appears to be survival analysis using the initial diagnosis of dry AMD as time 0, and conversion to wet AMD as the outcome.

Note that other analysis methods were also carried out, but are not reported here (see the appendix for Poisson regression results). These alternative simpler methods produced the same general conclusions as the survival analysis.

The survival analysis was stratified by propensity score quintiles. That is, eyes were divided into five (nearly equal size) groups using the quintiles of the propensity scores.

The following plots show the cumulative wet AMD conversion by propensity score stratum.

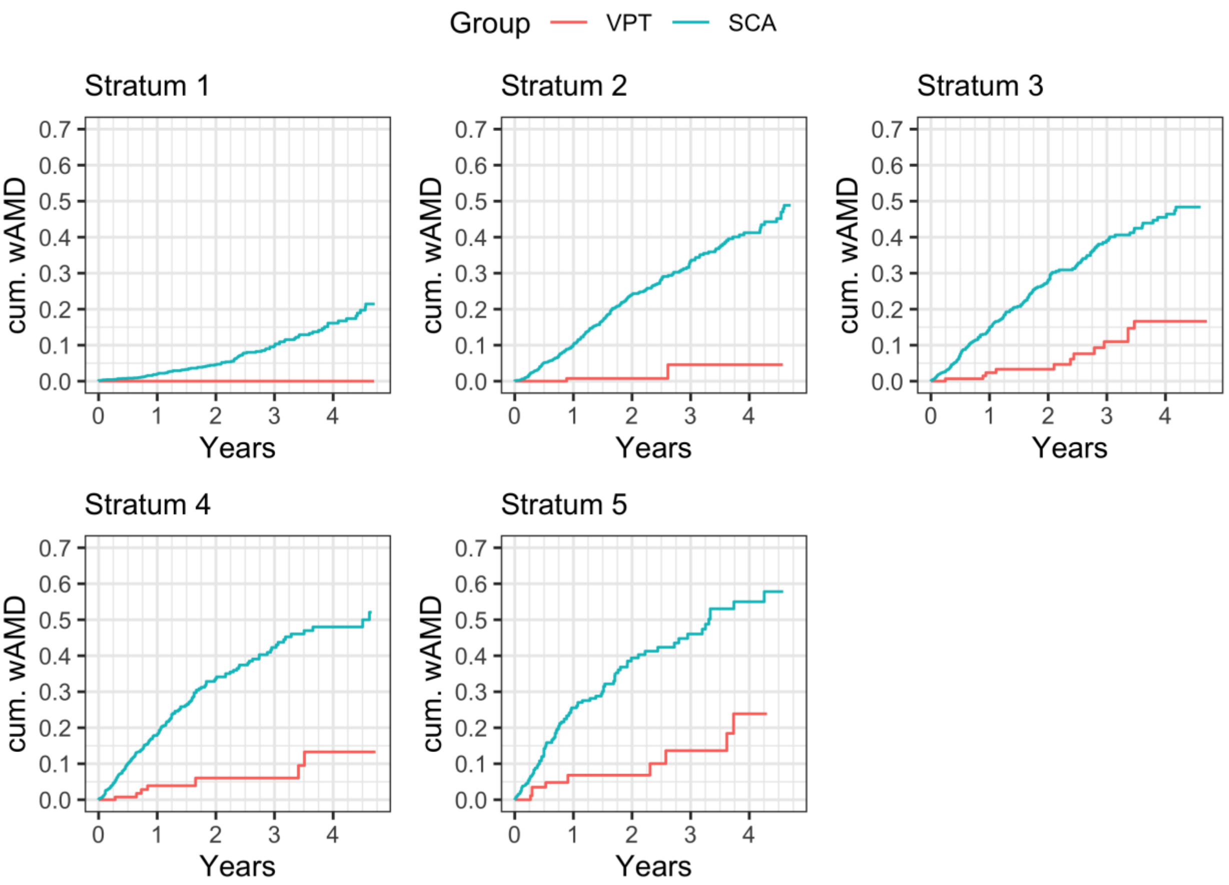

Figure 1. Cumulative probability of wAMD conversion by propensity score strata, VPT Non-Laser Included, no Encounter Matching.

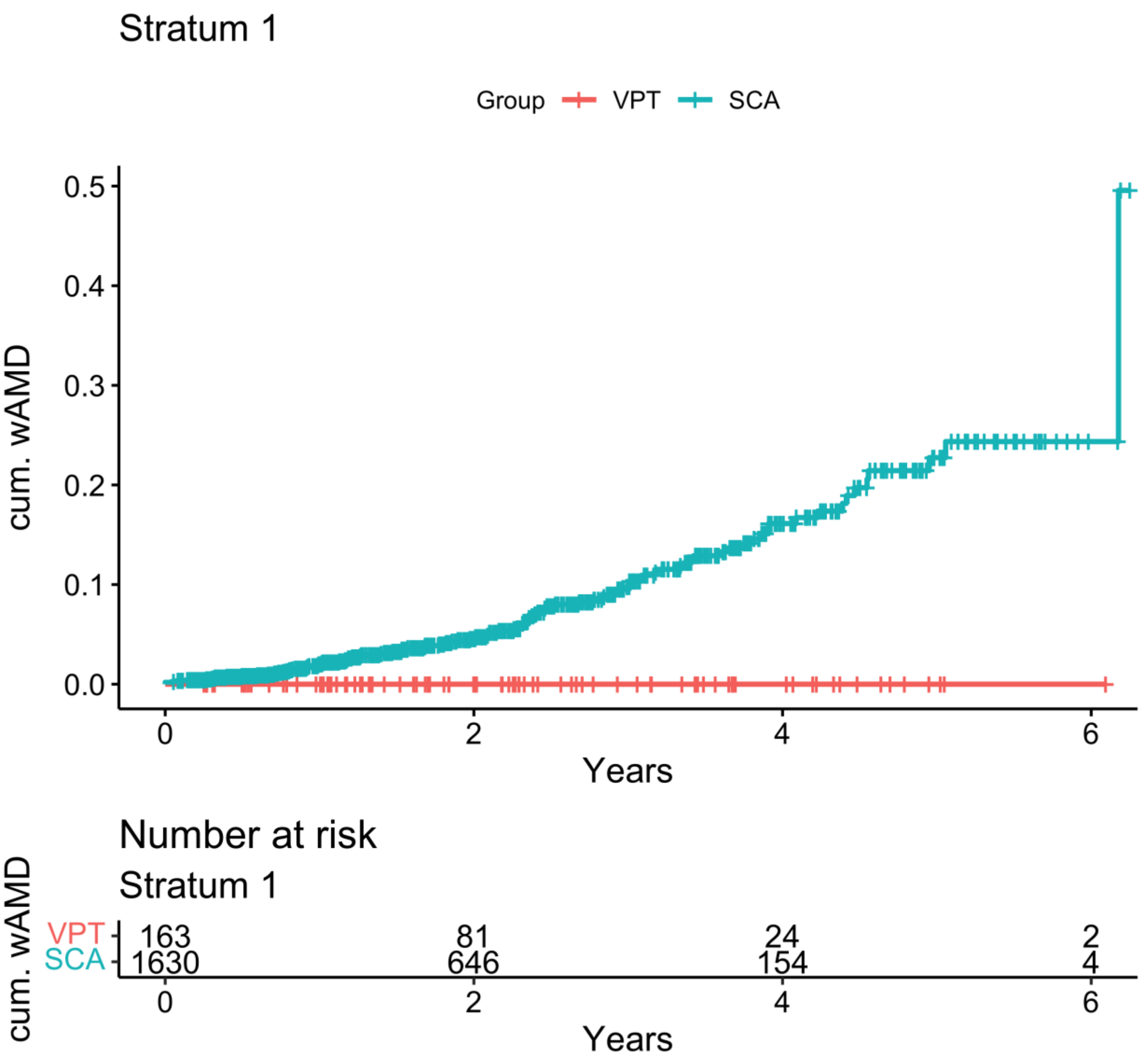

Stratum 1
Group VPT SCA
cum. wAMD
0.5
0.4
0.3
0.2
0.1
0.0
0
2
4
6
Years
Number at risk
Stratum 1
cum. wAMD
VPT 163 81 24 2
SCA 1630 646 154 4
0
2
4
6
Years

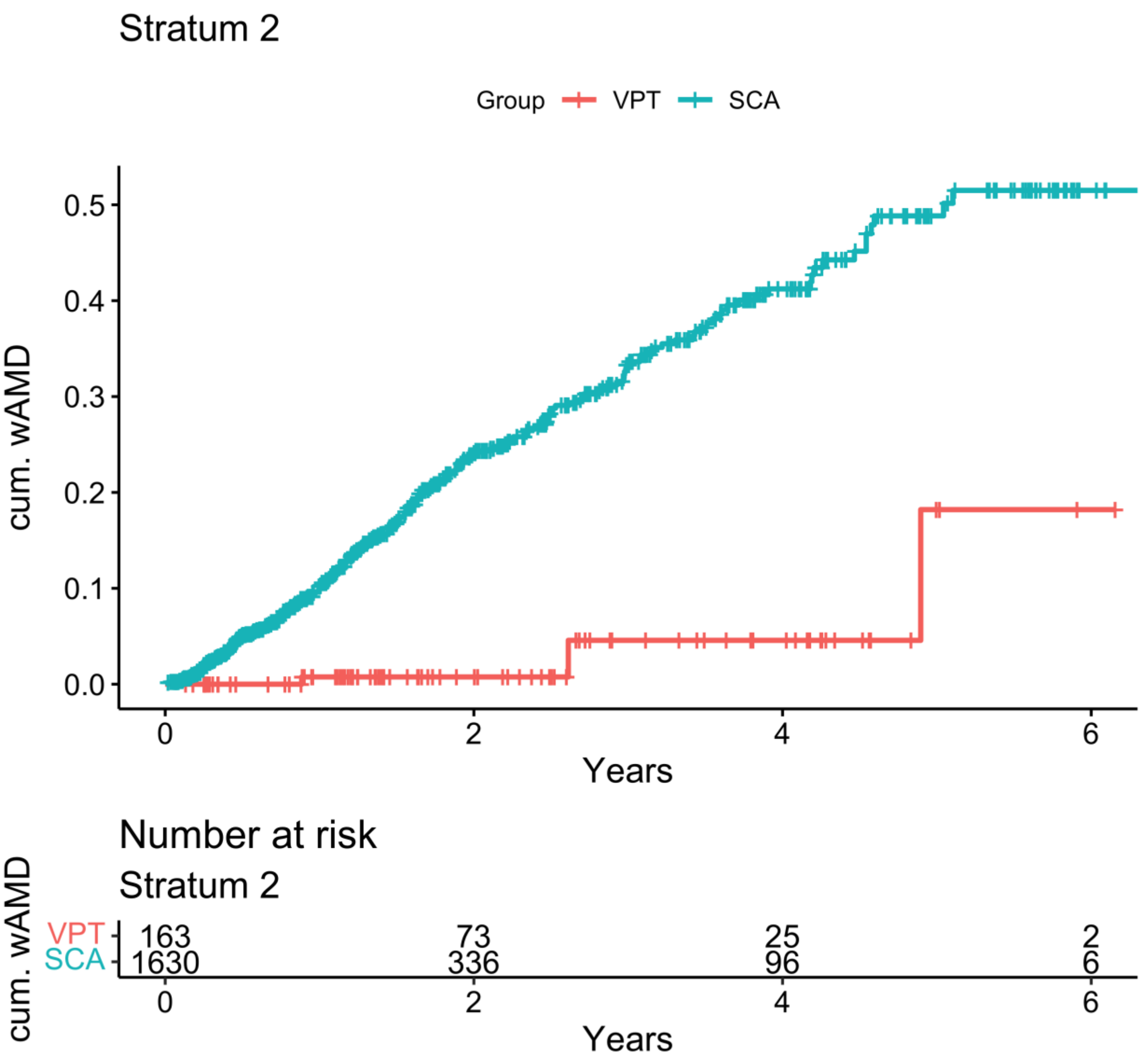

Stratum 2
Group VPT SCA
cum. wAMD
0.5
0.4
0.3
0.2
0.1
0.0
0
2
4
6
Years
Number at risk
Stratum 2
cum. wAMD
VPT 163 73 25 2
SCA 1630 336 96 6
0
2
4
6
Years

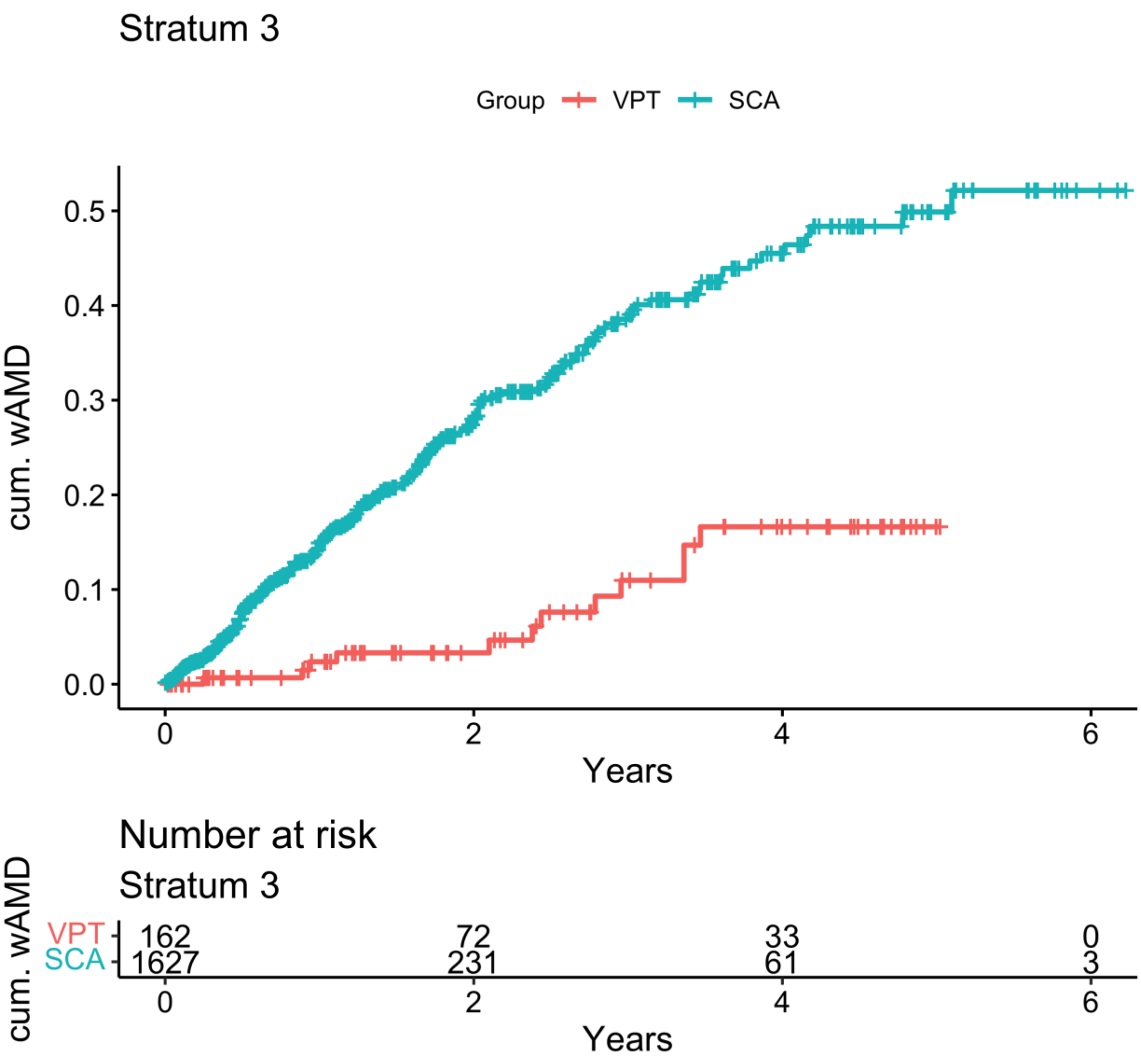

Stratum 3
Group VPT SCA
cum. wAMD
0.5
0.4
0.3
0.2
0.1
0.0
0
2
4
6
Years
Number at risk
Stratum 3
cum. wAMD
VPT 162 72 33 0
SCA 1627 231 61 3
0
2
4
6
Years

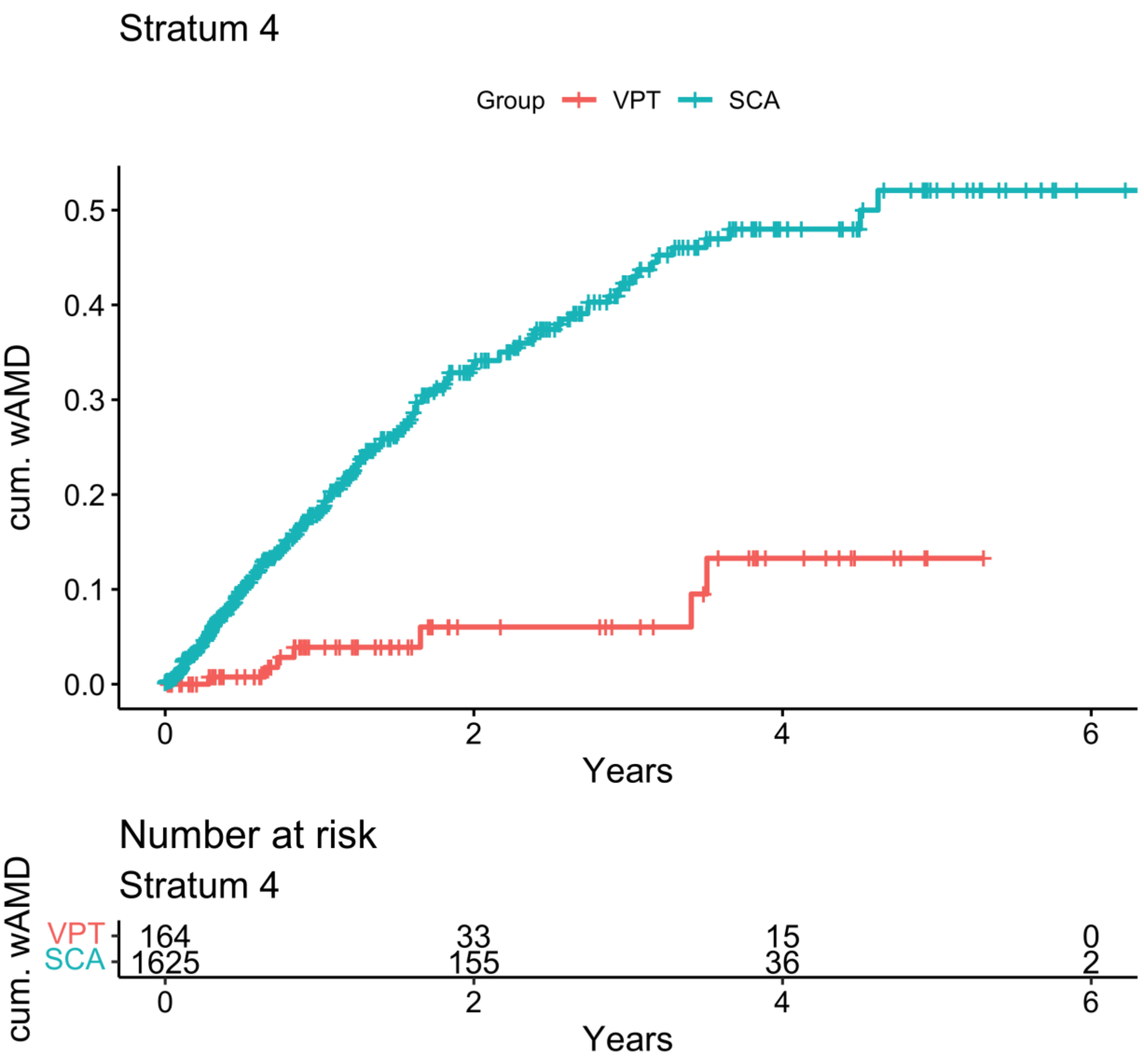

Stratum 4
Group VPT SCA
cum. wAMD
0.5
0.4
0.3
0.2
0.1
0.0
0
2
4
6
Years
Number at risk
Stratum 4
cum. wAMD
VPT 164 33 15 0
SCA 1625 155 36 2
0
2
4
6
Years

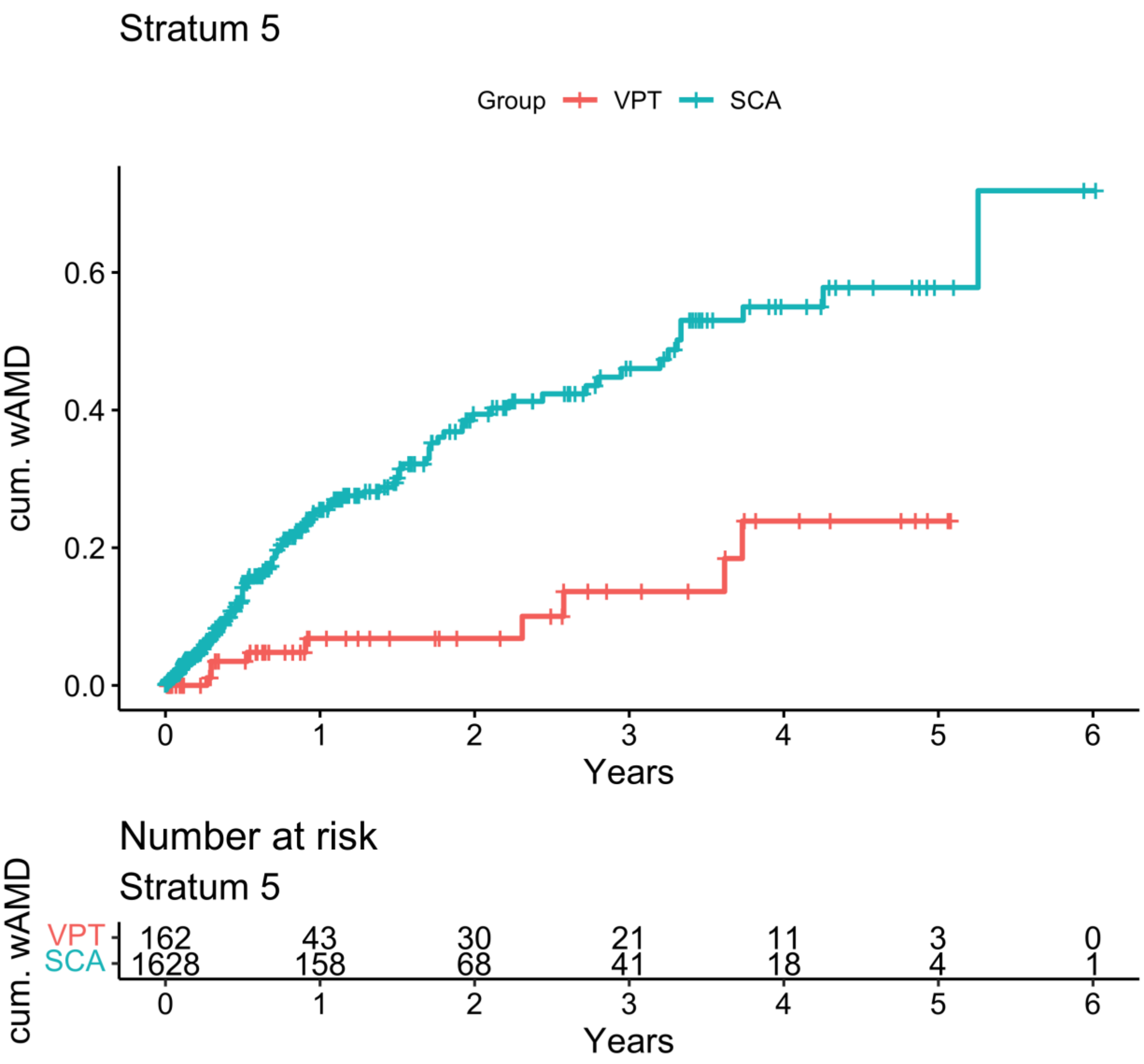
Stratum 5
Group
VPT
SCA
cum. wAMD
0.0
0.2
0.4
0.6
Years
0
1
2
3
4
5
6
Number at risk
Stratum 5
VPT 162 43 30 21 11 3 0
SCA 1628 158 68 41 18 4 1

A test for equality of the Kaplan-Meier survival curves (a stratified log-rank test) shows a very significant difference in survival between the VPT and SCA groups.

Table 6. Kaplan-Meier test between Groups, stratified by propensity score quintiles. Chisq = 123.167995 on 1 degrees of freedom, p = 0.000000

	N	Observed	Expected	(O-E)^2/E	(O-E)^2/V
**Analysis.Group=VPT**	814	32	157.9	100.4	123.2
**Analysis.Group=SCA**	8140	934	808.1	19.61	123.2

To allow for inclusion of covariates, and to provide an overall summary of the results, we carried out a Cox proportional hazards regression, again stratified by propensity score quintiles.

Cox PH modelinig initially included all of the covariates included in the propensity score calculation. Non-significant variables were dropped, until the final model included the following important covariates:

- Severity
- Age
- AREDS use
- Analysis Group

Results from the Cox proportional hazards fit are shown in the following Table.

Table 7. Cox PH summary of survival difference, VPT Non-Laser Included, no Encounter Matching. Estimated hazard ratios are in the column labeled exp(coef).

	coef	exp(coef)	se(coef)	z	p
**SeverityUnspecified**	0.5535	1.739	0.1723	3.212	0.001318
**SeverityIntermediate**	0.5867	1.798	0.1145	5.125	2.972e-07
**SeverityNonCentralGA**	0.5503	1.734	0.1537	3.581	0.0003426
**SeverityCentralGA**	0.3611	1.435	0.1618	2.233	0.02557
**Age**	0.01508	1.015	0.004002	3.768	0.0001643
**Areds.FlagYes**	0.2701	1.31	0.07485	3.609	0.0003075
**Analysis.GroupSCA**	1.732	5.651	0.1806	9.591	0

Likelihood ratio test=243.38 on 7 df, p=0 n= 8954, number of events= 966

For severity, the “Early” group (used as the reference group in the CPH model) had the highest hazard, with all of the other severity levels producing a hazard ratio < 1. Depending on which of the 4 analysis data sets were used, several other severity levels showed significant increase in hazard over the reference group.

Subjects with AREDS use showed a significant increase in hazard (30%-50%, depending on analysis data set).

For all analysis data sets, there was a significant increase in hazard for the SCA group, of about 5.75 after adjusting for severity, age, and AREDS use (here we have (HR = 5.65, p = <1e-04)).

The following forest plot shows the parameter estimates and confidence intervals from the Cox PH regression.

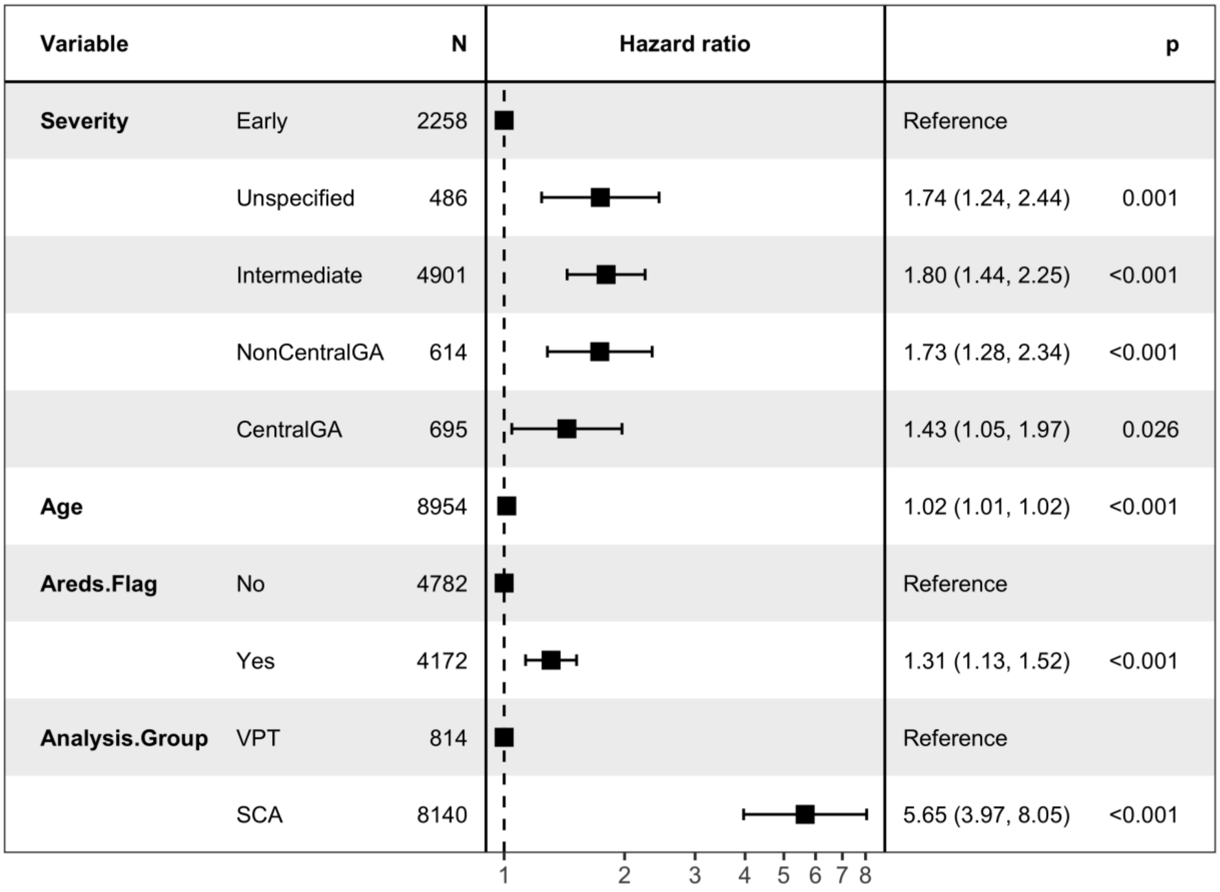

Figure 2. Forest plot of Cox PH results, VPT Non-Laser Included, no Encounter Matching

The test for Analysis Group shows a high level of significance.

Table 8. ANOVA for Cox PH model, VPT Non-Laser Included, no Encounter Matching.

	loglik	Chisq	Df	Pr(>|Chi|)
**NULL**	-6047	NA	NA	NA
**Severity**	-6022	50.14	4	3.381e-10
**Age**	-6015	13.77	1	0.0002062
**Areds.Flag**	-6007	17.65	1	2.658e-05
**Analysis.Group**	-5926	161.8	1	4.518e-37

A test for the proportional hazards assumption shows strong evidence of non-proportionality (cox.zph(), p = 0.0073). However (see appendix), various diagnostic plots do not indicate strong non-proportionality in the Cox PH model.

Table 9. Tests for proportional hazards violations., VPT Non-Laser Included, no Encounter Matching.

	chisq	df	p
**Severity**	9.802	4	0.0439
**Age**	0.7428	1	0.3888
**Areds.Flag**	2.012	1	0.156
**Analysis.Group**	6.03	1	0.01406
**GLOBAL**	19.3	7	0.007296

Summary of Survival Fits, VPT Non-Laser Included, no Encounter Matching

The following plot shows the overall cumulative wet AMD conversion probabilities by group, ignoring covariates.

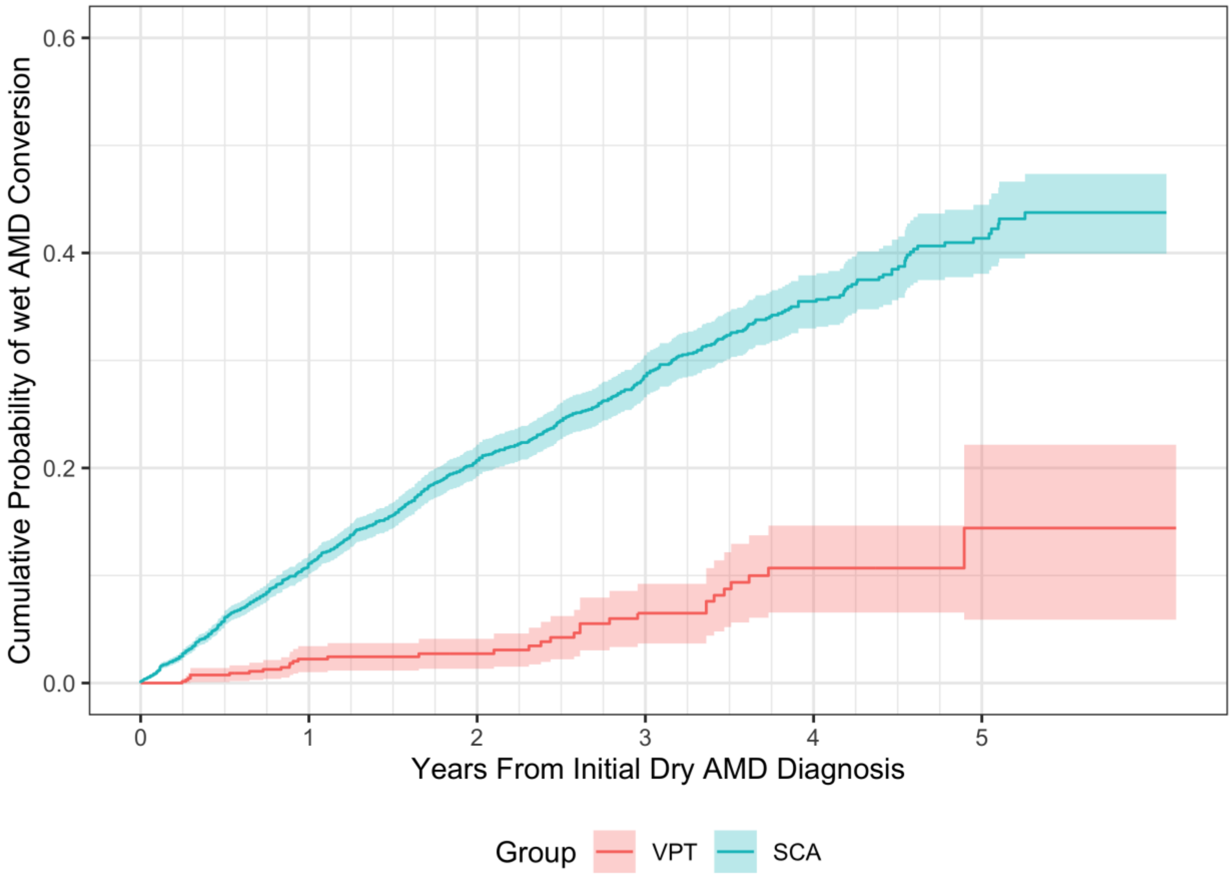

Figure 3. Overall Kaplan-Meier cumulative wet AMD conversion probability by group, VPT Non-Laser Included, no Encounter Matching. Shaded areas indicate 95% confidence intervals.

The following Table shows the cumulative probability of progressing to wet AMD, by year and group.

Table 10. Summary of overall survival by group (unstratified Kaplan-Meier estimates), VPT Non-Laser Included, no Encounter Matching.

Analysis Group	Years From DAMD Diagnosis	n at risk	n events	Cumulative Probability of wet AMD	95% CI
VPT	1	483	13	2.2%	[1.0%, 3.4%]
	2	289	2	2.7%	[1.3%, 4.1%]
	3	185	9	6.5%	[3.7%, 9.2%]
	4	108	7	10.7%	[6.6%, 14.6%]
SCA	1	2725	511	11.1%	[10.1%, 12.0%]
	2	1436	227	20.7%	[19.2%, 22.2%]
	3	768	108	28.6%	[26.6%, 30.5%]
	4	365	58	35.5%	[33.0%, 37.9%]

The hazard ratio between the two groups is summarized in the following Table. Since there are multiple eyes per person, a clustered bootstrap (clustered by subject) was used to provide a robust check on the confidence interval. The lower bound on the 95% confidence interval for the hazard ratio is above 4 using either method, again providing strong evidence for a hazard ratio greater than 1.

Table 11. Cox proportional hazards estimated hazard ratio and associated confidence intervals, VPT Non-Laser Included, no Encounter Matching. Cox PH model is stratified by propensity score quartiles.

Estimated Hazard Ratio	95% CI (asymptotic)	95% CI (bootstrap[1])
5.7	[4.0, 8.1]	[4.0, 9.0]

1-Bootstrap confidence interval is based on 10000 cluster (subject level) bootstrap samples.

Visual Acuity, VPT Non-Laser Included, no Encounter Matching

Visual acuity (ETDRS letters or the equivalent) was measured for a subset of subject visits (usually non-treatment visits). The SCA group averaged about 505.9 VA measurements per month, the VPT group averaged 52.3.

A tabulation of the mean VA by year shows a slight downward trend for the SCA group (perhaps due to aging?) but no obvious differences between the two groups.

Table 12. Mean visual acuity (ETDRS letters or equivalent) by Group and Year, VPT Non-Laser Included, no Encounter Matching. Cox PH model is stratified by propensity score quartiles.

Analysis Group	Mean VA 2017	Mean VA 2018	Mean VA 2019	Mean VA 2020	Mean VA 2021	Mean VA 2022
VPT	69.3	67.2	68.4	70.4	68.3	67.6
SCA	66.2	66.8	66.0	65.3	64.1	63.5

The following plot shows the mean VA per month for the SCA and VPT subjects. As expected there is more noise in the much smaller VPT group. Given the amount of noise it is difficult to assess whether there are any differences in VA through time.

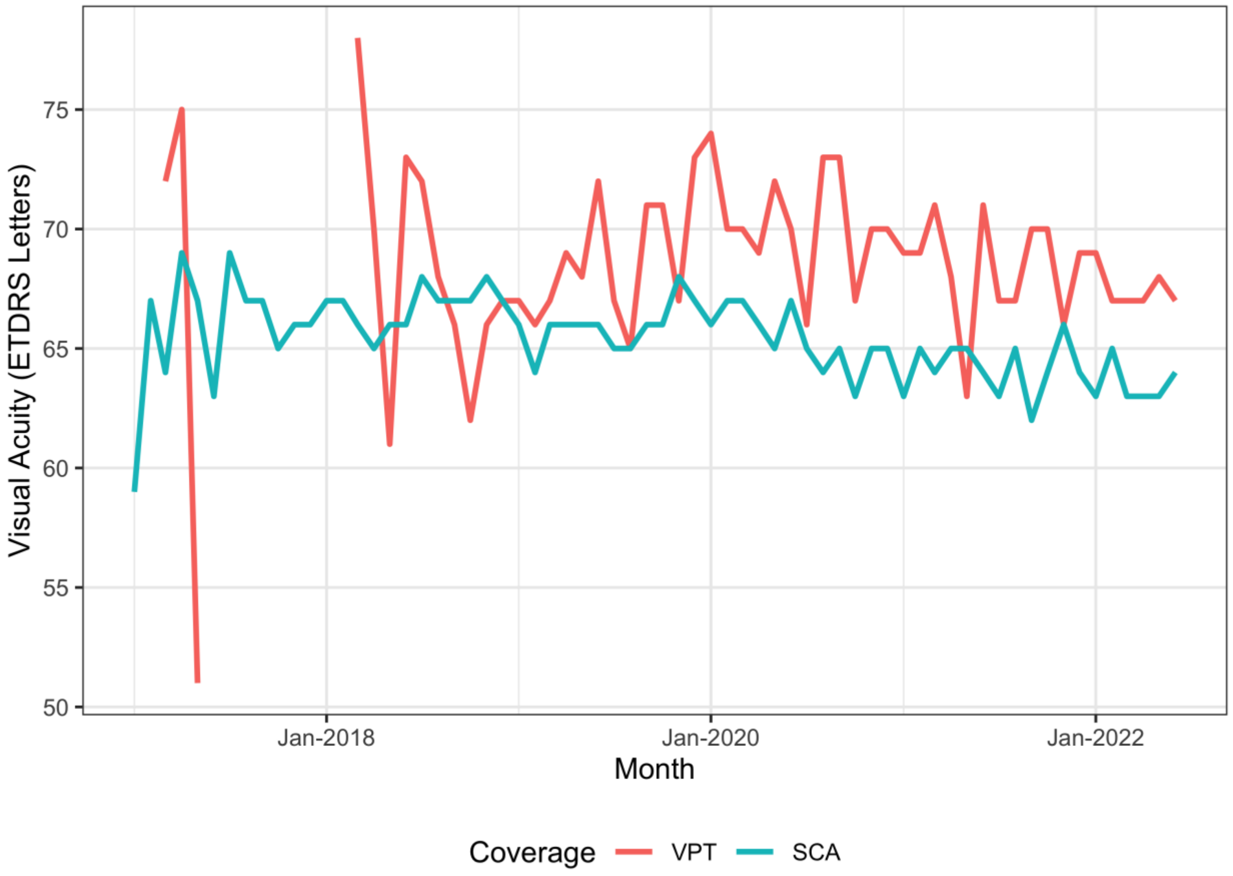

Figure 4. Visual Acuity by month, SCA, VPT Groups, VPT Non-Laser Included, no Encounter Matching.

A loess smoother shows a difference between the two groups during 2020-2021, perhaps due to the COVID pandemic?

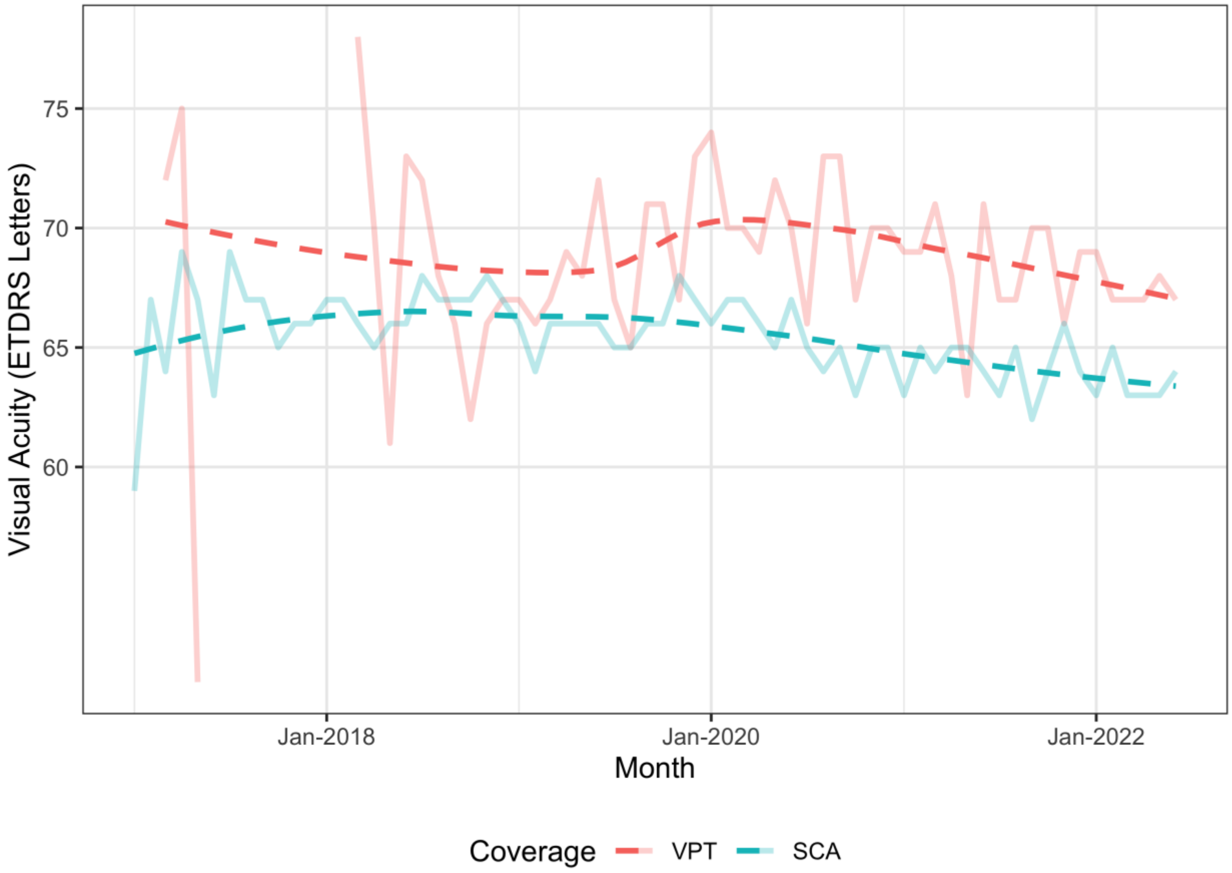

Figure 5. Visual Acuity by month, SCA, VPT Groups, with loess smooths., VPT Non-Laser Included, no Encounter Matching.

Caveats for Propensity Score Analysis

Potential shortcomings of this propensity score analysis include:

- The VPT group only includes subjects from a single practice, thus the generalizability of the results may be in question.
- Propensity score methods can “balance” the two groups with respect to the variables used in the propensity score model. However, they do not balance for unmeasured covariates, thus if there are other important predictors of group membership or outcome that are missing the results could be misleading.
- This analysis used subjects for whom the latest ICD-10 coding was used, which added Dry AMD severity codes not present in the earlier ICD-9. We are thus using the site-level assessment of AMD severity and assume a reasonably consistent diagnostic judgement across sites. There is no reading center or verification of the coding as would be implemented in a clinical trial.
- The ICD-10 coding used in the Vestrum database may not capture all subject outcomes, including potentially some adverse events.
- There are inconsistencies in ICD coding, for example some subjects who had a series of anti-VEGF injections were never coded as having converted to wet AMD. Thus an eye was considered converted to wet AMD if the appropriate ICD code was entered **and** at least one anti-VEGF injection was administered. The conversion date was set to earliest of the ICD coding date or the date of the first anti-VEGF injection. Alternative definitions of conversion do not change the basic conclusions.
- There are differences in follow-up time and number of encounters (reported subject-physician interactions) between the two groups, with the VPT group having more encounters and longer follow-up. As these are “post-randomization” outcomes they were not used in the initial propensity score matching. The longer follow-up in the VPT group most likely produced more events, whereas the effect of increased encounters, if any, is unknown. Thus if there is any bias due to follow-up it is likely to produce a smaller hazard ratio for SCA versus VPT.
- To match follow-up intensity between the two groups a second propensity score analysis used the mean time-between-visits for each subject as an additonal matching variable. This produces a better correspondence in follow-up time and number of visits between the two groups at the expense of potential bias issues with using post-diagnosis information in the matching.

Appendix/Supplemental

This appendix includes diagnostic plots and alternative analyses.

Distribution of Follow-Up Time

The following plot shows the follow-up time by group and wet AMD status.

Note the spikes in the SCA group at approximately 3 months, 6 months, 1 year, 2 years, and 3 years of follow-up.

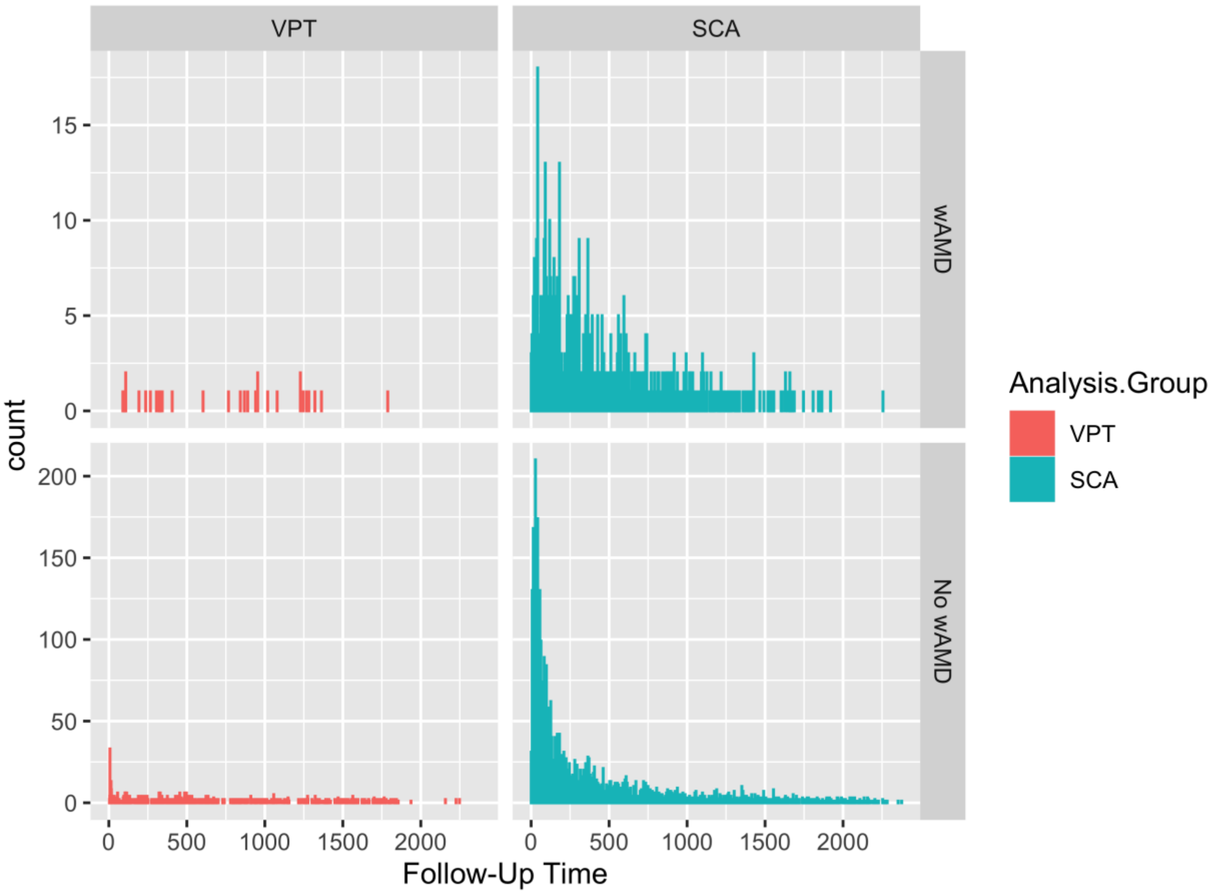

Figure 6. Follow up time by study group and wAMD conversion status. Subjects with 0 days follow-up are excluded.

Diagnostic Plots for Propensity Scores

Propensity score diagnostic plots look good overall. The overall distributions for the propensity scores are quite similar.

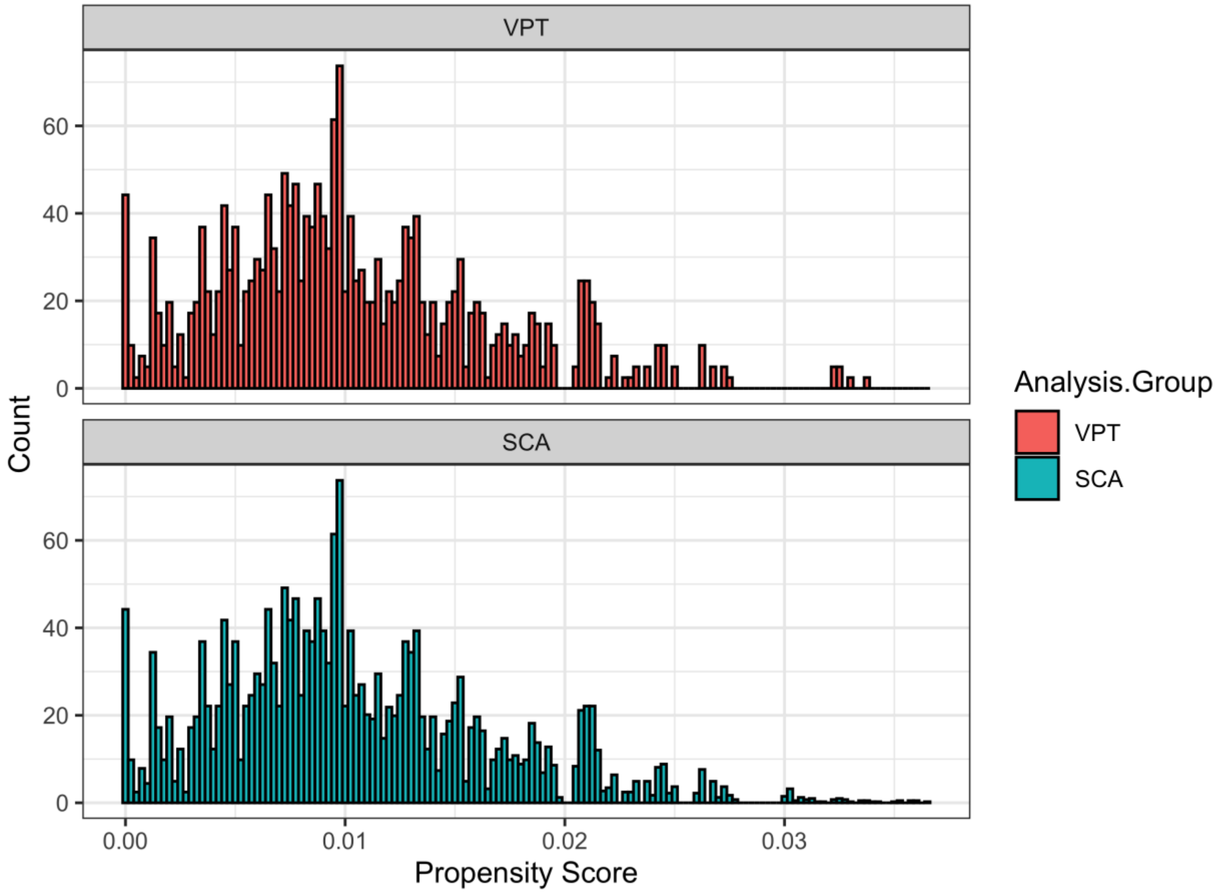

Figure 7. Diagnostic plots for propensity scores, overall distribution.

Propensity scores are also similar within each PS stratum.

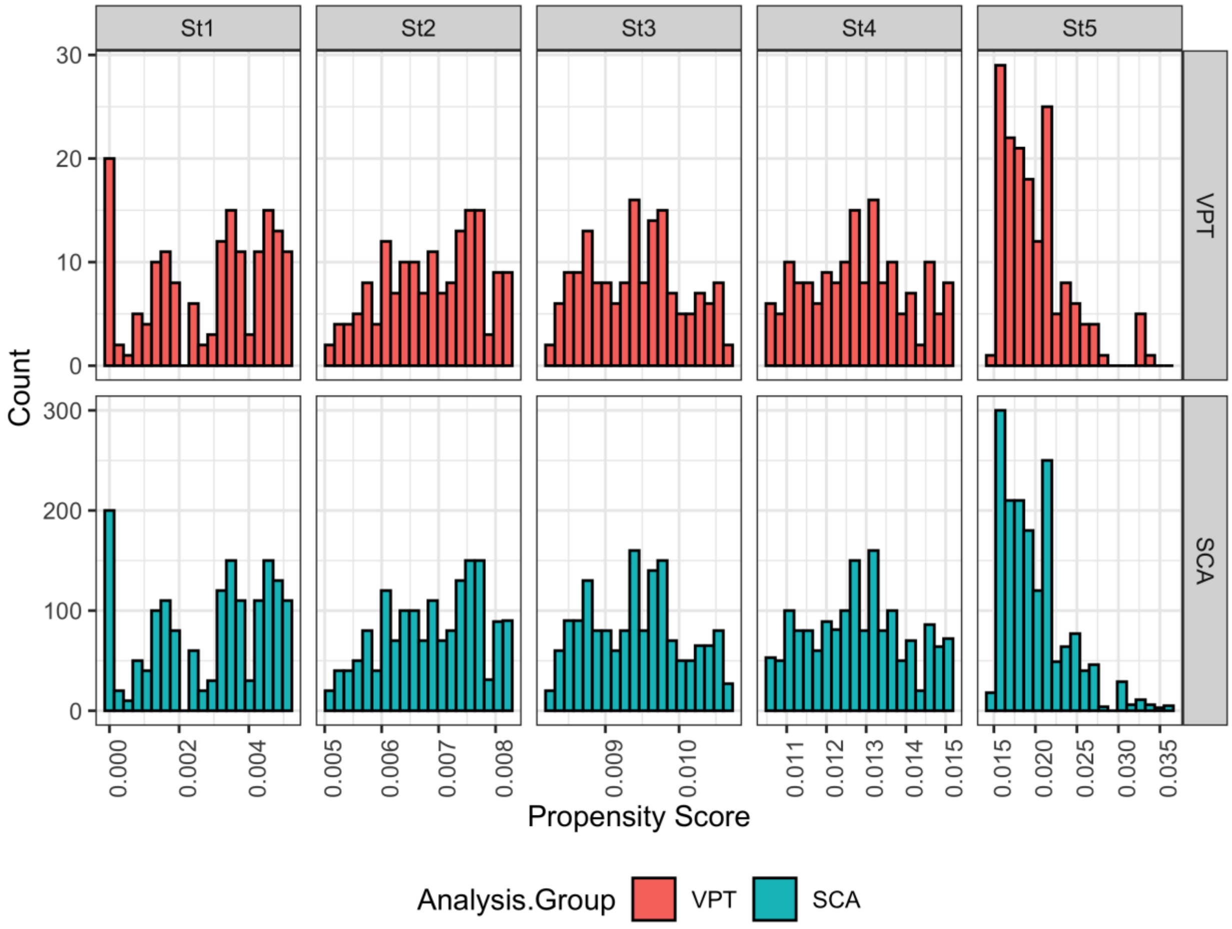

Figure 8. Diagnostic plots for propensity scores, distribution by PS stratum.

Each individual component also looks good. What we want to see is that within each stratum the two groups are similar (e.g. the pairs of orange and yellow bars have good overlap, the pairs of green bars are similar height).

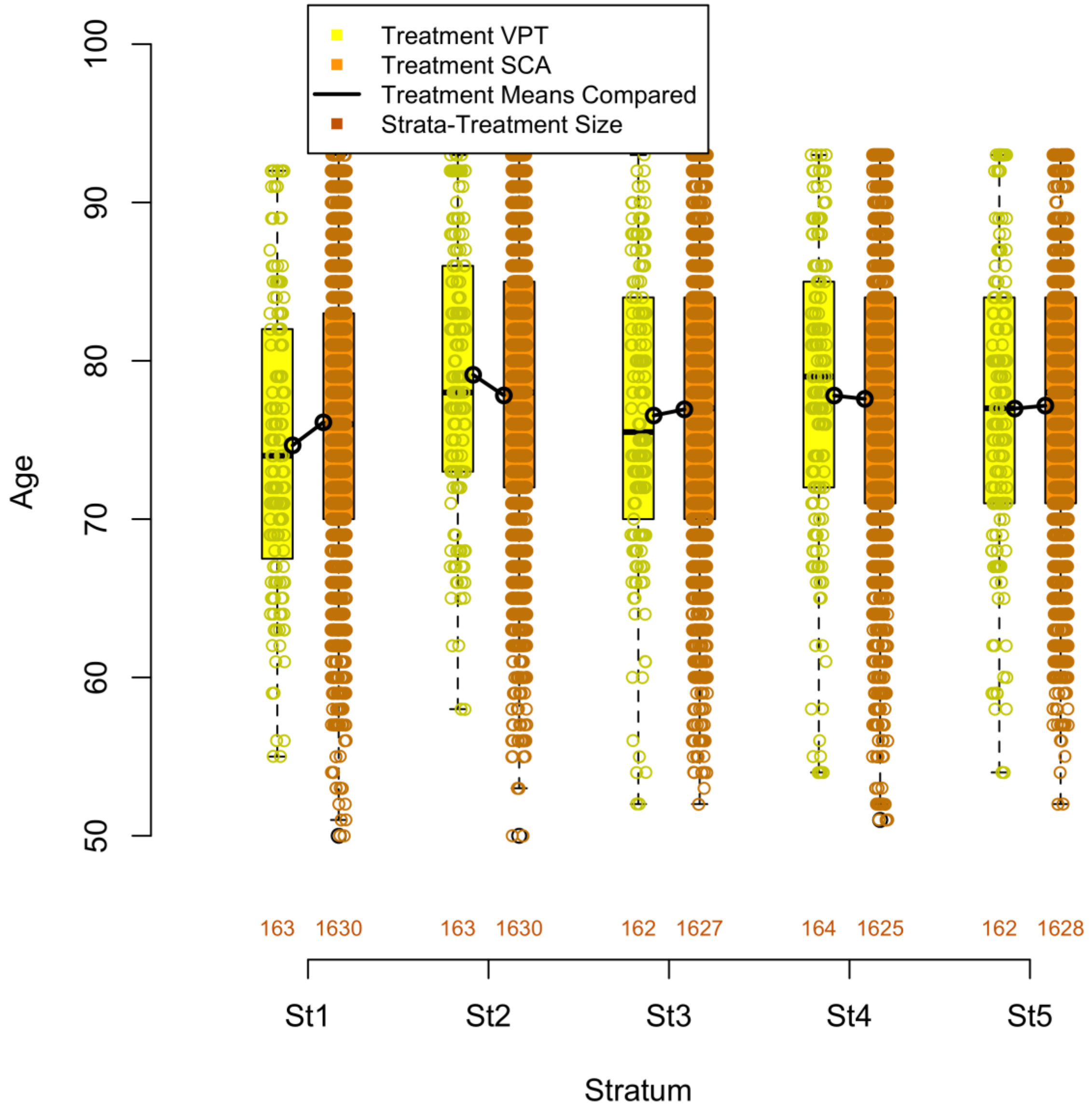

Figure 9. Diagnostic plots for propensity scores, age.

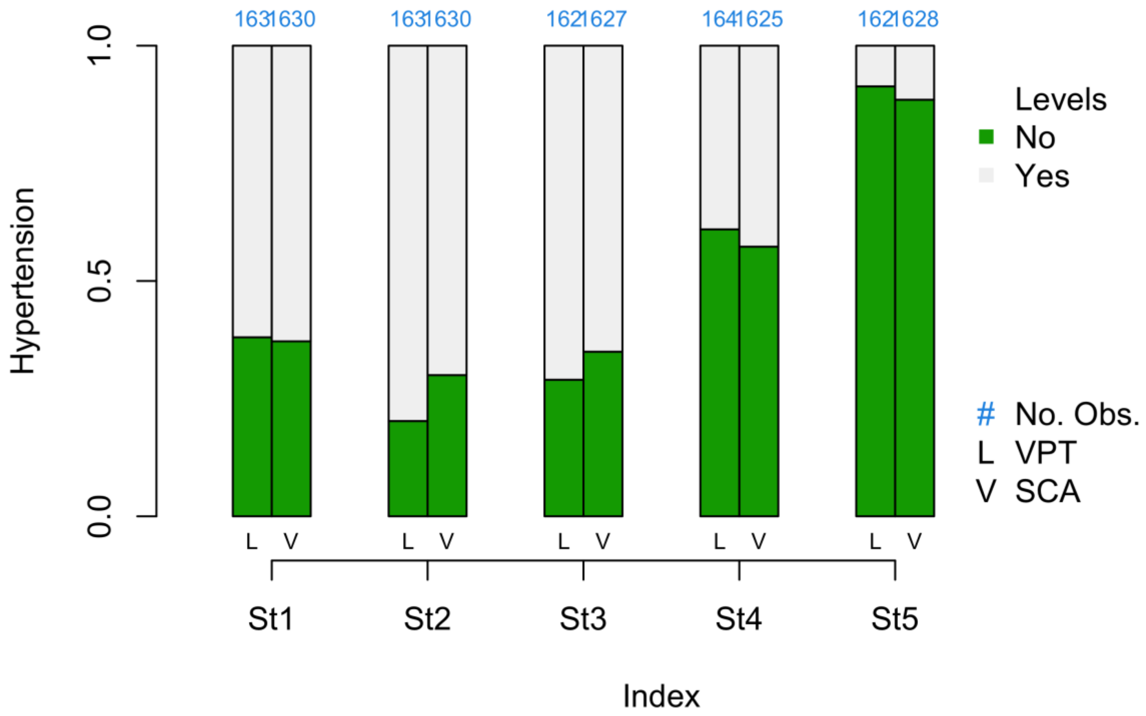

Figure 10. Diagnostic plots for propensity scores, hypertension.

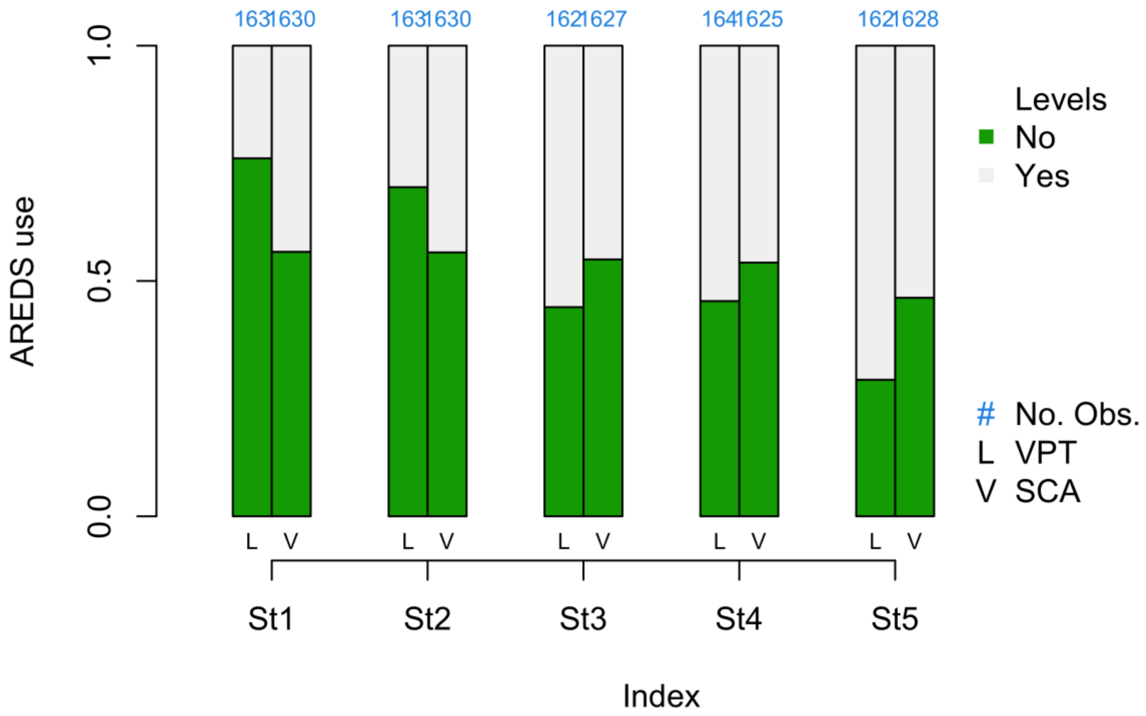

Figure 11. Diagnostic plots for propensity scores, AREDS use.

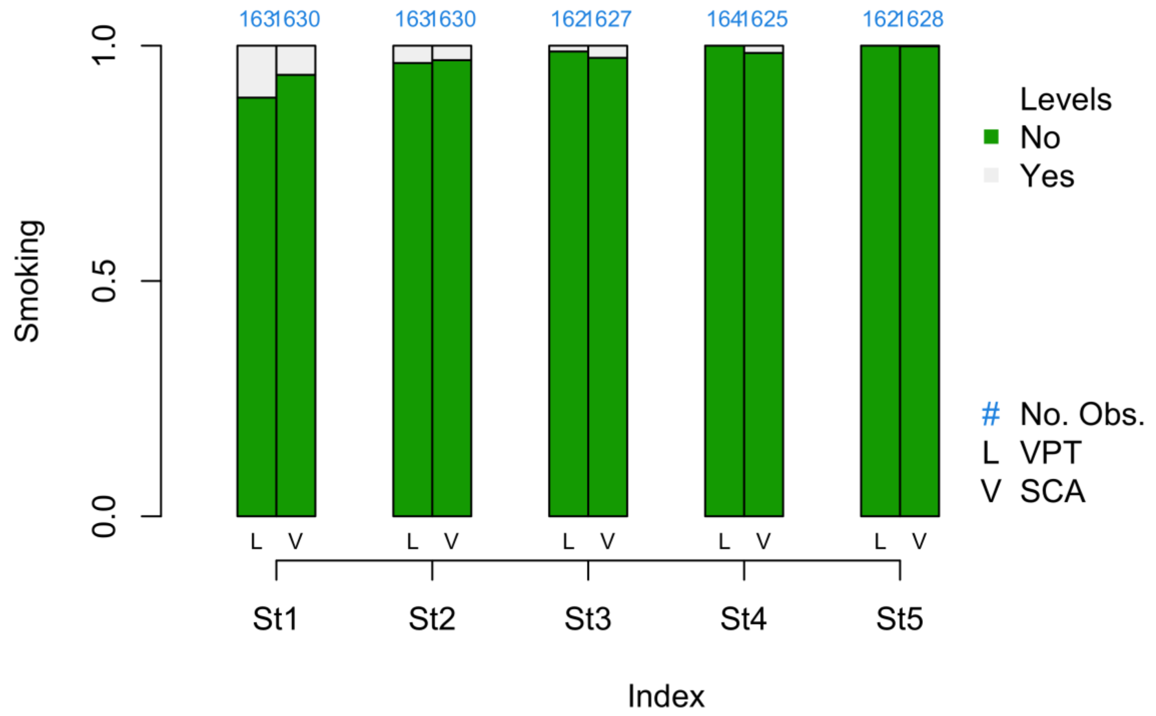

Figure 12. Diagnostic plots for propensity scores, smoking.

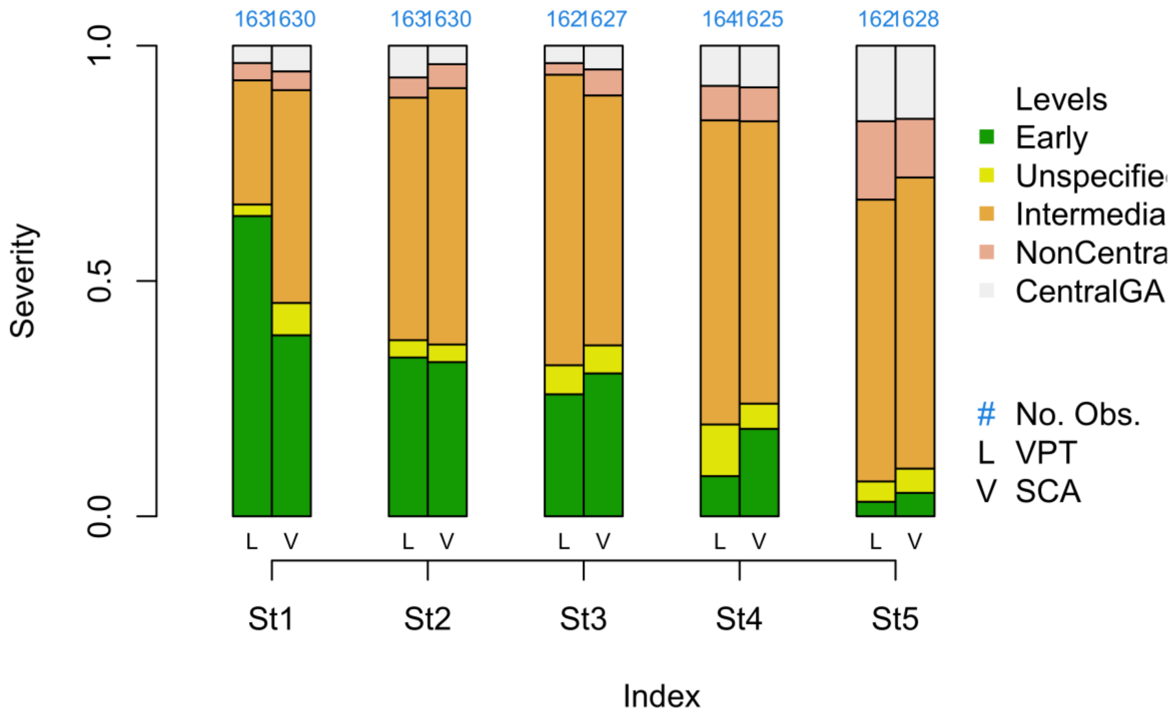

Figure 13. Diagnostic plots for propensity scores, severity

Incidence Rates via Poisson Regression

As a simpler alternative to the survival analysis, we can fit a poisson regression model. Poission regression adjusts for the follow-up on each eye individually, so corrects the bias in incidence rates somewhat. We can also easily account for the propensity score strata.

Fitting generalized (poisson/log) linear model: Converted.to.wAMD.n ~ Analysis.Group + PSStratum + offset(Follow.up.Years)

	Estimate	Std. Error	z value	Pr(>|z|)
**(Intercept)**	-7.77	0.2026	-38.36	0
**Analysis.GroupSCA**	1.922	0.18	10.68	1.319e-26
**PSStratumSt2**	1.244	0.1172	10.61	2.716e-26
**PSStratumSt3**	1.61	0.1182	13.62	2.922e-42
**PSStratumSt4**	1.812	0.1204	15.05	3.449e-51
**PSStratumSt5**	2.172	0.1274	17.05	3.799e-65

Table 13. Incidence rates from poisson regression (correcting for unequal follow-up in data, and adjusting for stratum differences.)

Analysis.Group	incidence rate	std.error	df	null	statistic	p.value
VPT	0.004505	0.0007994	Inf	1	-30.44	1.429e-203
SCA	0.03078	0.001063	Inf	1	-100.7	0

The incidence rate ratio from this fit is 6.8.

Diagnostic Plots for Cox PH Model

The following plots are diagnostics from the Cox proportional hazards fit.

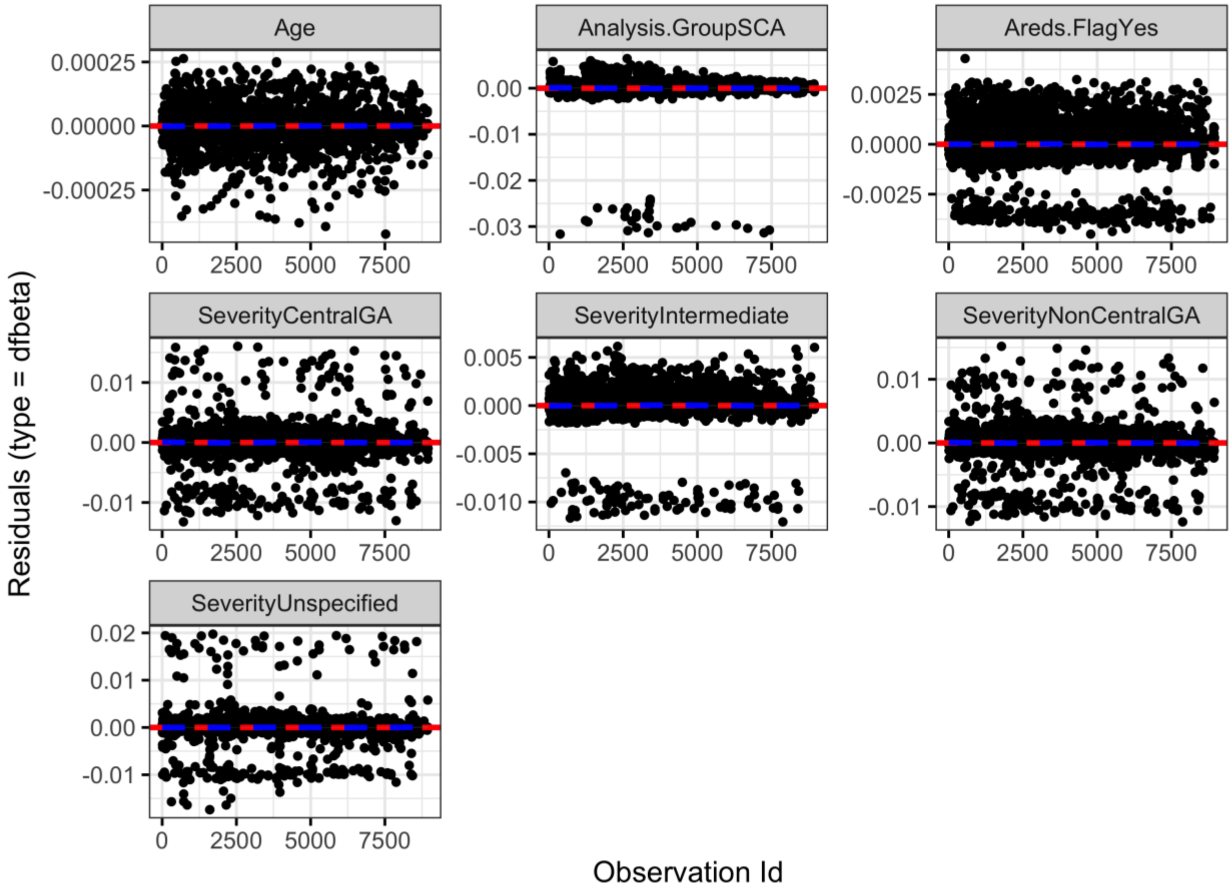

Figure 14. Dfbeta residuals from Cox PH fit.

```
## `geom_smooth()` using formula = 'y ~ x'
```

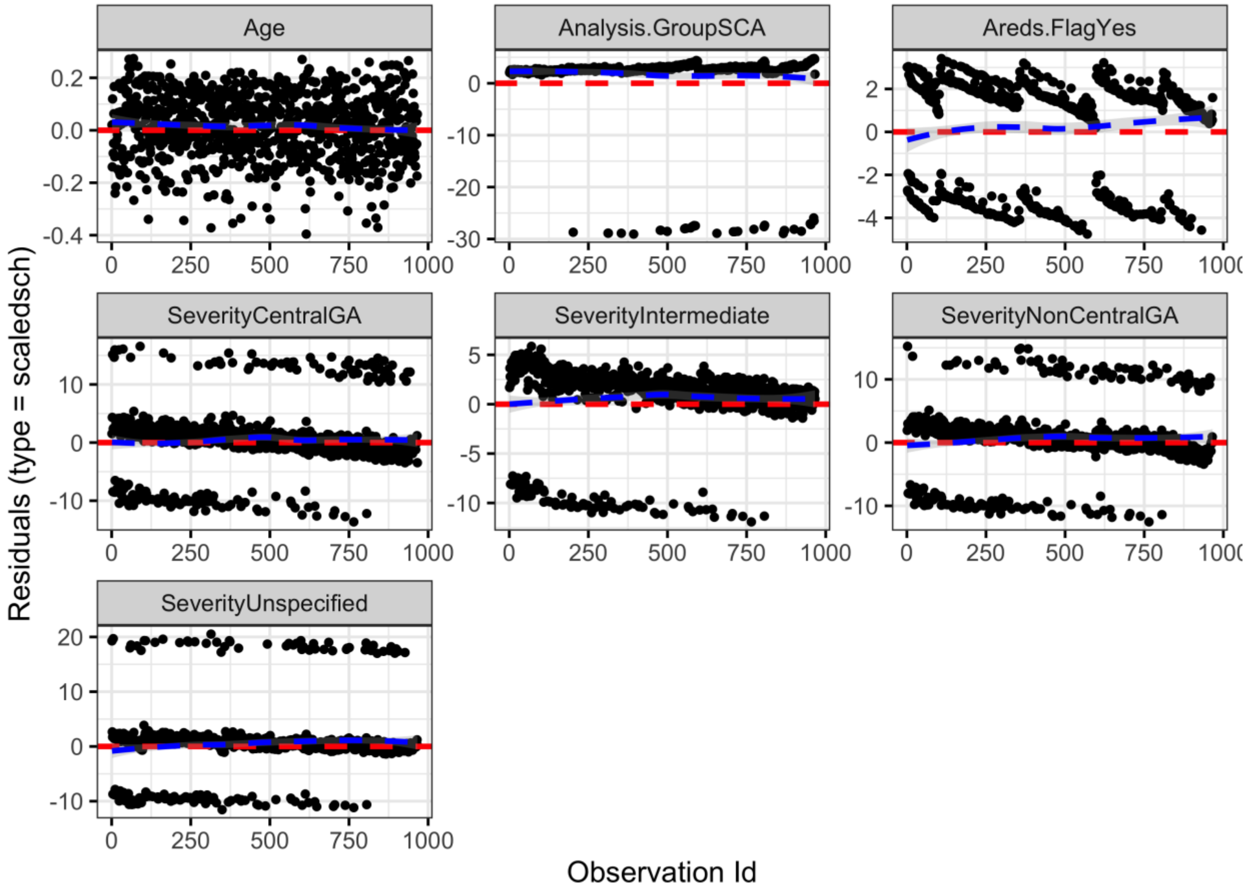

Figure 15. Scaled Schoenfeld residuals vs Time.
